# Adaptive strategies of aquatic mammals: Exploring the role of the HIF pathway and hypoxia tolerance

**DOI:** 10.1590/1678-4685-GMB-2023-0140

**Published:** 2024-01-19

**Authors:** Yuri Yépez, Mariana Marcano-Ruiz, Maria Cátira Bortolini

**Affiliations:** 1 Universidade Federal do Rio Grande do Sul, Departamento de Genética, Laboratório de Evolução Humana e Molecular, Porto Alegre, RS, Brazil.

**Keywords:** HIF pathway, HIF3A, HIF3α, aquatic mammals, hypoxia tolerance

## Abstract

Aquatic mammals (marine and freshwater species) share significant and similar adaptations, enabling them to tolerate hypoxia during regular breath-hold diving. Despite the established importance of *HIF1A*, a master regulator in the molecular mechanism of hypoxia response, and other associated genes, their role in the evolutionary adaptation of aquatic mammals is not fully understood. In this study, we investigated this topic by employing a candidate gene approach to analyze 11 critical genes involved in the *HIF1A* signaling pathway in aquatic mammals. Our gene analyses included evaluating positive and negative selection, relaxation or constriction of selection, and molecular convergence compared to other terrestrial mammals, including subterranean mammals. Evidence of selection suggested a significant role of negative selection, as well as relaxation of the selective regime in cetaceans for most of these genes. We found that the glutamine 68 variant in the HIF3α protein is unique to cetaceans and initial evaluations indicated a destabilizing effect on protein structure. However, further analyses are necessary to evaluate its functional impact and adaptive relevance in this taxon.

## Introduction

Aquatic mammals, including cetaceans, sirenians, and carnivores such as pinnipeds and some mustelids, have developed significant and similar adaptations to live in aquatic environments, despite their distinct evolutionary origins (Berta *et al.*, 2015, 2018; Fordyce, 2018). These adaptations, including anatomical, physiological, and biochemical changes, allow them to tolerate low oxygen tension or hypoxia, which is necessary for regular breath-hold diving, and permits them to withstand lower blood oxygen tension than most terrestrial mammals (Ponganis, 2011).

The physiological and biochemical mechanisms underlying the immersion behavior of aquatic mammals have been extensively explored in previous studies (Allen and Vázquez-Medina, 2019; McKnight *et al.*, 2019). Moreover, genomic investigations encompassing individual species and cross-lineage comparisons among marine mammals have unveiled various evolutionary adaptations to the aquatic environment. These adaptations encompass hypoxia tolerance, specialized energy metabolism, responses to oxidative stress, modifications in body plan, and anatomical changes (Yim *et al.*, 2014; Foote *et al.*, 2015; Zhou *et al.*, 2015; Chikina *et al.*, 2016; Fan *et al.*, 2019).

Despite these advancements, our comprehension of the genetic mechanisms governing hypoxia tolerance in aquatic mammals remains incomplete. To shed light on this aspect, it is essential to consider the role of the transcription factor HIF (Hypoxia Induced Factor), which assumes a central position in the hypoxia response across metazoans (Schofield and Ratcliffe, 2004; Kaelin and Ratcliffe, 2008). HIF consists of one of three α subunits (HIF1α, HIF2α, and HIF3α) and a common β subunit (HIF1β or ARNT), encoded by the genes *HIF1A*, *HIF2A*, *HIF3A*, and *ARNT*, respectively. HIF’s primary function is to activate the transcription of genes that enhance oxygen delivery or facilitate metabolic adaptation to hypoxic conditions.

Under normal oxygen levels, specific prolyl residues and one asparaginyl residue within HIFα undergo hydroxylation (Schofield and Ratcliffe, 2004; Kaelin and Ratcliffe, 2008). Three HIF prolyl-hydroxylases (PHDs 1-3), encoded by the genes *EGLN1*, *EGLN2*, and *EGLN3*, hydroxylate the prolyl residues, while the factor inhibiting HIF (FIH1), encoded by the *HIF1AN* gene, hydroxylates the asparaginyl residue. Hydroxylated prolyl residues serve as binding sites recognized by the von Hippel-Lindau protein (encoded by *VHL*), leading to the degradation of HIFs through the ubiquitin-proteasome pathway. The hydroxylation of the asparaginyl residue causes steric hindrance, preventing the binding of specific transcriptional co-activators. However, under hypoxic conditions, the hydroxylation of HIFα by PHDs is inhibited, resulting in the accumulation of HIF. This accumulated HIF translocates into the cell nucleus, where it forms dimers with HIF1β and binds to specific DNA motifs of target genes. This binding activates the transcription of these target genes, like *VEGFA*, which encodes a growth factor that plays a crucial role in promoting vascular endothelial cell proliferation (see Figure S1 for a visual representation of this process). 

Multiple studies in human populations, and high altitude plateau and fossorial mammals have provided robust evidence that the hypoxia signaling pathway (also HIF pathway) plays a crucial role in hypoxia tolerance in low oxygen environments (Xiao *et al.*, 2017; Witt and Huerta-Sánchez, 2019). As a result, it is not surprising that HIFs and other proteins that interact with these transcription factors are involved in mammalian adaptation to aquatic environments. Convergent evolution has been observed in three genes of HIF pathway (*HIF1A, HIF2A,* and *EGLN3*), as demonstrated in a comparison between five cetacean species and high-altitude mammals (Zhu *et al.*, 2018).

Further insight into the functional adaptations of pathway proteins in cetacean species has been gleaned from *in vitro* studies, revealing variable responses to different oxygen tensions. For example, differences in HIF1α were observed in three cetacean species (Bi *et al.*, 2015), and unique functions of VHL associated with HIF2α degradation were reported in beluga whales (Bi *et al.*, 2017). *In vivo* studies in elephant seals (*Mirounga angustirostris*) have shown HIF1α stabilization in response to fasting and sleep apneas, further supporting the significance of the HIF pathway in hypoxia tolerance (Vázquez-Medina *et al.*, 2011).

As we have access to an expanding list of aquatic mammal genomes, we can gain a more comprehensive understanding of the genes that are involved in the adaptation to hypoxia associated with an aquatic lifestyle in mammals. To this end, we propose a study that examines the molecular evolutionary patterns of the coding region of 11 genes that encode proteins of the HIF pathway, by comparing the aquatic mammal sequences (marine and freshwater species) with those of terrestrial mammals, including hypoxia tolerant fossorial species.

## Material and Methods

### Hypoxia signaling pathway genes and genomic data collection

We searched for 11 genes involved in the evolutionarily conserved cellular signaling pathway of responses to hypoxia, *ARNT* (also termed as *HIF1β*), *ARNT2* (*HIF2β*), *HIF1AN* (*FIH1*), *HIF1A* (*HIF1α*), *EPAS1* (*HIF2α*), *HIF3A (HIF3α*), *EGLN1*, *EGLN2*, *EGLN3*, *VHL*, and *VEGFA* (Table S1; Figure S1). We gathered genetic and functional information from the GeneCards Database (2023) and consulted the gene interaction prediction program STRING v11.0 (2023) to analyze gene interactions.

We obtained the orthologous coding sequences (CDS) of 11 genes from the NCBI Orthologs database. We utilized data from 21 NCBI annotated genomes and CDS from 35 terrestrial mammal species (Table S2). Additionally, we incorporated assembled genome data from 19 aquatic mammal species, which lacked annotation in the NCBI database. To enhance our dataset, we included draft genome assemblies from 11 aquatic mammal species that were publicly available, as provided by the DNA ZOO consortium (Dudchenko *et al.*, 2017).

To retrieve the orthologous CDS, we employed a BLAST-based approach. Subsequently, we utilized the Geneious software (Kearse *et al.*, 2012) to map the sequences from these genomes with the CDS references (Table S3), resulting in contiguous consensus sequences.

### Sequence alignments

We used GUIDANCE2 (Landan and Graur, 2008; Sela *et al.*, 2015) with the PRANK codon alignment algorithm to align the CDS of the genes. We eliminated any sequences or columns with uncertain confidence scores (threshold <6) from the original dataset before conducting further analyses.

### Phylogenetic trees

We reconstructed phylogenetic trees from the 11 HIF pathway gene alignments using Bayesian inference with BEAST v.1.10.4 (Suchard *et al.*, 2018) for the subsequent selection analyses.

### Selection analyses

To identify natural selection signals, we employed several statistical tests based on ω values. Site-level model analyses (M2 and M8 models) using PAML v.4.9 CODEML software (Yang, 2007) were conducted, as well as the CODEML branch-site Test 2 to detect positive selection along a specific branch of the tree. The study also used HyPhy 2.5.31 software (Kosakovsky Pond *et al.*, 2020) with the RELAX method, FUBAR, MEME, and BUSTED to identify positive selection in genes where neither the site nor the branch-site methods detected positive selection signals.

### Selection pressure analysis in cetacean clades with contrasting diving abilities

We analyzed selection pressure in cetacean clades with different diving abilities using the nested branch model approach proposed by Tian *et al.* (2016). Species of cetacean and pinnipeds were classified into deep-divers (≥300 m of common depth and ~40 minutes of average dive time) and shallow-divers (common depth around 100 m in shallow waters; Table S4).

### Molecular convergence analysis

To identify convergent amino acid substitutions in aquatic mammals, we employed the Profile Change with One Change (PCOC) method (Rey *et al.*, 2018). This method employs a criterion that characterizes convergent shifts as substitutions occurring on all branches where a change in phenotype has occurred, and these substitutions align with a modification in the preferred type of amino acid at that specific position.

### Structural analyses

We accessed predicted three-dimensional protein structures of HIF pathway proteins from the AlphaFold database (Varadi *et al.*, 2022). To visualize and map positively selected sites onto these three-dimensional protein structures, we employed the UCSF ChimeraX 1.5 software (Pettersen *et al.*, 2021). Additionally, to predict the impact of specific variants on protein stability, we conducted ΔΔG (Gibbs free energy of unfolding) analyses using the DDGun3D software (Montanucci *et al.*, 2019). 

### Additional information

For a more comprehensive explanation of these and previous analyses, and detailed information about genes and sequences, please refer to the Supplementary materials and methods section. We also utilized OpenAI’s GPT-3.5 model to proofread and enhance the English phrasing and grammar in our manuscript.

## Results

We conducted a candidate gene approach to analyze 11 HIF pathway genes CDS across over 86 mammalian species in order to improve our understanding of their evolution. Our analysis compared species with aquatic lifestyles, including 35 cetacean species, 10 pinniped species,6 Lutrinae species, and one manatee species, with those with terrestrial habits, such as subterranean species of rodents.

### Relaxation analysis

To investigate the possible changes in selective pressures that occurred in HIF pathway genes during the transition to an aquatic lifestyle, we used the HYPHY RELAX method. Initially, we performed a joint analysis of the four aquatic mammals taxa, followed by separate analyses of each taxon. Our results provide strong evidence of changes in selective regimes, including both relaxation (K < 1) and intensification (K > 1) of selection. When considering the set of aquatic lineages, we observed relaxation in four genes: *ARNT, ARNT2, EGLN2*, and *HIF1AN* (Table S5). Further analysis by taxa revealed relaxation in six genes, including the four genes mentioned above, *EPAS1* and *EGLN1*, and evidence of intensification in *VEGFA*, all in cetaceans. In the Pinnipedia clade, evidence of relaxation was detected in one gene, *HIF1AN*. The Lutrinae group, on the other hand, exhibited an intensification of the selective pressure in *EGLN3*. Interestingly, no evidence of change in selective pressures was observed in any HIF pathway genes for *Trichechus manatus*.

### Signatures of positive selection

To gain insight into the impact of selection on the evolution of HIF pathway genes in aquatic mammal lineages, we utilized a gene-wide test for episodic diversification, BUSTED, with aquatic taxa specified as foreground branches. Positive results from this test would indicate that at least one site was positively selected in at least one period of time on the specified branches. The BUSTED analysis revealed evidence of selection at low frequencies of sites (0.01% to 1.55%) in only three genes (*ARNT, ARNT2, EPAS1*) among aquatic mammals (see Table S6).

Although BUSTED is technically a Branch-site test, it is not designed to detect individual sites under selection. Therefore, we implemented different methods based on codon substitution to detect positive selection at the site level. Our analysis using CODEML models M2 and M8 showed that most genes fit better with the null models M1a and M7, respectively. However, positive selection was detected at seven sites in *HIF3A* and *VHL* by the M2 model, while the less conservative M8 model identified 21 sites with positive selection signature in *ARNT*, *HIF1AN*, *HIF1A*, and *EPAS1*. The sites under positive selection as identified by the BEB analysis are presented in Table S7.

Using MEME and FUBAR, we identified a total of 136 codons from 10 genes with evidence of positive selection (Table S8). Specifically, FUBAR identified 16 positively selected sites in 7 genes, while MEME detected 134 sites in 10 genes (except *HIF1AN*). Notably, MEME is designed to detect both episodic and pervasive selection, whereas FUBAR only identifies sites under pervasive positive selection (Berta *et al.*, 2015). Thus, the difference in the number of sites detected by each method is likely due to a large proportion of sites detected by MEME being associated with episodic selection events. We identified 24 sites from 7 genes (5 in *HIF3A*, 7 in *VHL*, 3 in *ARNT*, 1 in *HIF1A*, 3 in *EPAS1*, 3 in *EGLN2*, 2 in *VEGFA*) as robustly under positive selection, as they were identified by at least two methods at the site level (Table 1). We identified large portions of each gene under negative selection using FUBAR, refer to Table S9 for details.

**Table 1 -:** Positively selected sites of mammal HIF pathway genes.

>Gene	>Site	PAML			HYPHY		Protein domain
		Site model M8 (BEB PP > 0.90)	Site model M2 (BEB PP > 0.90)	Branch-site model A (BEB PP > 0.90)	FUBAR (PP > 0.90)	MEME (p-value < 0.1)	
*ARNT*	271	0.934			0.989	0.02	
	705				0.925	0.06	
*EGLN2*	9				0.928	0.03	
	55				0.916	0.01	
	155				0.912	0.09	
*EPAS1*	265				0.9	0.05	
	493				0.942	0.09	PAS 2
	762	0.995				0.06	
*HIF1A*	599	0.904			0.928	0.04	Inhibitory domain
*HIF3A*	68			0.955		0.00	bHLH
	364	0.995	0.997			0.05	
	427	0.917				0.08	
	437	0.963	0.963				
	598				0.987	0.00	
*VEGFA*	31				0.905	0.09	
	144				0.923	0.03	
*VHL*	8				0.956	0.04	
	23	0.995	0.972			0.00	
	28				0.984	0.08	
	68	0.996	0.996				
	125	1	1				Binding to CCT complex
	195	1	1			0.02	
	212	1	1				

The sites presented were detected by at least two codon-based methods. Description of protein domains available in Uniprot database (UniProt Consortium, 2019).

To confirm positive selection and investigate specific signals in individual sites across aquatic mammal taxa Cetacea (order Artiodactyla), Pinnipedia (order Carnivora), and Lutrinae (Mustelidae; Carnivora) compared to their terrestrial counterparts, we employed a Branch-site method known as Test 2 or the branch-site test of positive selection, implemented in CODEML. Our analysis identified only one site, at position 68, of HIF3α protein under episodic selection in the cetacean lineage (Table 1). This site was also detected by MEME, with support from a branch, and it has an exclusive arginine residue in this group. No amino acid residues unique to aquatic mammal lineages were observed at sites with evidence of positive selection found by more than one method, except for this case.

Additionally, we found two different sites, 437 and 195, in HIF3α and VHL, respectively, that are unique to cetaceans and hippopotamuses, both of which are members of the suborder Whippomorpha. These sites did not exhibit amino acid residues exclusive to subterranean Rodentia species (*e.g*., *Fukomys damarensis*, *Nannospalax galili*, and *Heterocephalus glaber*), or even of the other Artiodactyla species, such as *Vicugna pacos* (family Camilidae) adapted to live in Andean high-altitude, where the hypoxic environment is also well documented.

### Branch models and diving contrasts

To investigate whether the selective pressures acting on HIF pathway genes differed between cetaceans and pinnipeds with distinct abilities to dive and remain submerged, we employed CODEML branch models using the nested design of Tian *et al.* (2016). In cetaceans, we classified the species from the taxonomic families Balenopteridae, Balaenidae, Eschrichtiidae, Lipotidae, Phocoenidae, Iniidae, Pontoporiidae, and three species from the family Delphinidae as deep-divers, and species from the families Ziphiidae, Physeteridae, Monodontidae, and three additional species from Delphinidae as shallow-divers. In pinnipeds, we classified species from the family Phocidae as shallow-divers, and those from the Otariidae and Odobenidae families as deep-divers (Table S4).

**Figure 1 -:**
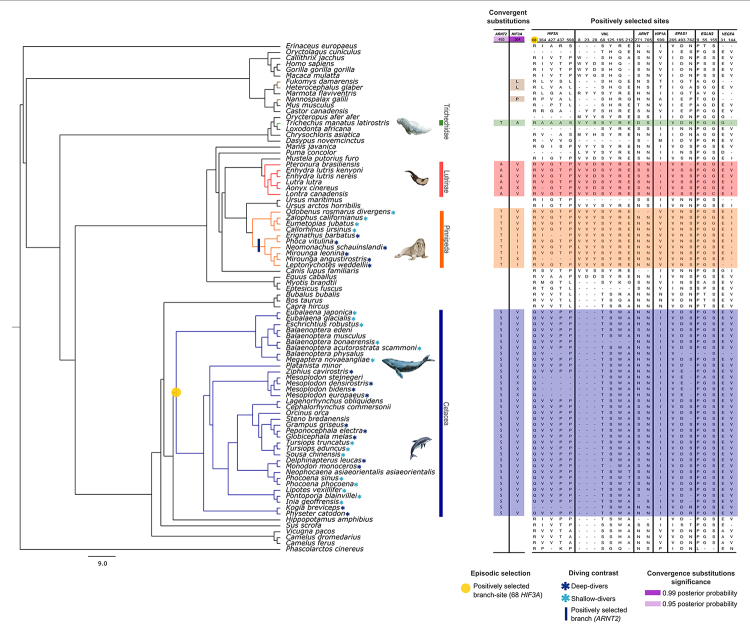
Positive selection and convergence patterns of HIF pathway genes have been analyzed across the mammalian phylogeny. Aquatic mammal clades have been highlighted. A Bayesian inferred phylogeny of concatenated loci is depicted, with changes across positively selected sites confirmed by two or more natural selection inference analyses. Corresponding amino acids for each species are listed on the right side of the figure. *ARNT2* and *HIF3A* genes have also been shown to exhibit signals of molecular convergence with the PCOC method under two convergence scenarios: considering only aquatic mammals and including aquatic mammals and subterranean rodent species. PCOC model posterior probability values of convergent amino acids are indicated. Asterisks in the species names indicate their diving capabilities classified as deep or shallow divers, for representative species and clades. The blue vertical line indicates evidence of a positively selected branch on *ARNT2* detected using the nested branch model implemented in CODEML, and the yellow circle indicates episodic selection in the cetacean lineage in *HIF3A* detected using MEME and CODEML branch-site Test 2.

The LRTs showed significant differences between the 1ω vs. 2ω models for four genes in cetaceans and eight genes in pinnipeds, indicating that these two aquatic taxa have experienced different selective pressures compared to their terrestrial counterparts. Table S10 provides the corresponding values. Notably, pinnipeds displayed significant changes in selective pressures towards higher values of ω when compared to terrestrial lineages and when comparing deep-diving and shallow-diving pinnipeds. The *ART2* analysis revealed a ω value of 0.0188 for deep-diving pinnipeds, which is considerably higher than that of shallow-diving pinnipeds (Figure 1). However, there were no significant differences when considering ancestral and descendant branches (see Table S10).

### Convergence analysis

We utilized the PCOC method to evaluate potential cases of adaptive convergent amino acid evolution across the 11 HIF pathway proteins. This method calculates the posterior probabilities of convergent evolution at each site under the “PCOC” model. We considered two scenarios: the first encompassing general adaptations to an aquatic lifestyle across all aquatic mammal groups, and the second scenario including subterranean rodent species due to their shared phenotypes of hypoxia tolerance due their respective habitats.

In the first scenario, we detected two candidate sites for convergent substitutions in two proteins: site 485 in ARNT2 and site 356 in HIF3α (position 364 in human reference sequence). Both sites had a posterior probability greater than 0.95 under the PCOC model (Figure 1). Additionally, site 364 in HIF3α showed positive selection signals detected by three methods. Most amino acids at this site had hydrophobic side chains. In the second scenario that included subterranean rodent species (*Fukomys damarensis*, *Heterocephalus glaber*, and *Nannospalax galili*) with a similar phenotype of hypoxia tolerance, we found only one significant convergent substitution, also at site 356 in HIF3α.

### Protein structures

We proceeded with the mapping of amino acid changes indicated by positive selection in the predicted 3D protein structures. When we marked putative selected sites detected by more than one codon substitution method, we found that most of these sites were located in disordered regions of the proteins. However, approximately 22% (5/23) of these sites were within or close to domains of these proteins. 

Based on the available protein structure and sequence information, we tested the effect of three amino acid changes, R68Q and T437P in HIF3α and Q195W in VHL, on protein stability (Table 2). R68Q, a change unique to cetaceans, occurs in the second alpha helix motif of the bHLH domain of HIF3α (Figure S2). This substitution replaces an arginine (R) residue, which has a cationic side chain, with a glutamine (Q) residue, which has a polar uncharged side chain, and was predicted to decrease protein stability (ΔΔG = -0.1) by both the sequence-based and structural-based methods DDGun and DDGun3D, respectively. In contrast, we did not observe any specific changes in protein stability due to the T437P substitution (Figure S2). Finally, the Q195W substitution, which changes a glutamine to a tryptophan (W) residue with a hydrophobic side chain in VHL, was predicted to stabilize the protein (ΔΔG = 0.1) by both methods (Figure S3). 

**Table 2 -:** DDGun analyses.

Protein	Variant	KD	BL	PROF	3D	RSA	Residue contacts	ΔΔG[SEQ]	ΔΔG[3D]
HIF3ɑ	R68Q	0.227	-0.136	0.379	0.151	52	R68: L64|R65|M66|H67|L69|C70|A71|A72|G73|E213|L216	R68Q: -0.1	R68Q: -0.1
	T437P	0.000	-0.036	0.008	0.004	94.000	L435|A436|R438|H439	T437P: 0.0	T437P: 0.0
VHL	Q195W	0.140	-0.763	-0.716	-0.297	45.0	P95|N193|V194|K196|D197|L198|E199	Q195W: 0.1	Q195W: 0.1

DDGun scores quantify protein stability effects of amino acid substitutions using sequence and structural information. Scores include KD (difference in hydrophobicity in Kyte-Doolittle scale), BL (difference in Blosum62 substitution matrix), PROF (difference in interaction energy with neighbors), 3D (difference in interaction energy with structural neighbors). RSA (residue solvent accessibility) modulates mutation effect with residue accessibility. DDGun combines KD, BL and PROF, while DDGun3D combines all four scores.

## Discussion

### Relaxed selection signatures

Shifts in the distribution of selection pressure are often associated with large biological transitions, such as the transition from land to water, which can lead to phenotypic plasticity and adaptive changes (Lahti *et al.*, 2009). In the case of lipid metabolism gene *CYP8B1*, for example, signs of relaxation of selection pressure were observed in the common ancestor of cetaceans (Shinde *et al.*, 2019), while the *MB* gene showed relaxation of constraints, reflected in the protein stability, in shallow-diving cetacean species (Holm *et al.*, 2016). The *RAG1* gene also showed a relaxation of selective pressure in cetaceans (Dias and Nery, 2020).

Relaxation of selection pressure can lead to gene pseudogenization by allowing the accumulation of stop codons or other changes in the nucleotide sequence that disrupt the reading frame, leading to gene loss events that may constitute an evolutionary mechanism of adaptation to different niches (Sharma *et al.*, 2018). In cetaceans, gene loss in ancestral species may indeed have contributed to adaptation to the aquatic environment (Huelsmann *et al.*, 2019; Espregueira Themudo *et al.*, 2020). Besides, relaxation of selective pressure is also a finding compatible with a scenario of conditional neutrality, a way for changes to accumulate and escape the action of purifying selection, resulting in variability (Raman *et al.*, 2016). Furthermore, there are various ways that selection can be relaxed, including the removal of the functional constraint, and reduction in efficiency of selection (*e.g*., increased relevance of genetic drift due to a reduction in effective population size; Charlesworth and Charlesworth 2010, Wertheim *et al.* 2015). 

We found that cetaceans are the representative group of aquatic mammals with the largest number of genes under a relaxation regime (6 of 11 HIF pathway genes studied). Interestingly, when considering all aquatic mammals in relation to their terrestrial relatives, fewer genes with relaxation or constriction signals were evident, and these signals were mainly observed in cetaceans. 

One of the genes that exhibits relaxed selection in pinnipeds is *HIF1AN*, also known as *FIH1*. This gene plays a crucial role in directly regulating HIFα, adding an additional layer of control to the HIF response (Baik and Jain, 2020). It complements the function of PHD proteins, which regulate HIFα levels through prolyl hydroxylation. This observation suggests that there might be a less stringent control mechanism in place for regulating HIF1α in cetaceans and pinnipeds.

In a broader context, these findings indicate that the proteins in cetaceans may have initially evolved under different functional constraints compared to those in pinnipeds and Lutrinae. This implies that relaxed selection could have played a significant role in shaping the evolutionary path of HIF pathway genes in cetaceans, particularly.

### Adaptive evolution

While several studies have explored various aspects of the evolution of marine mammals, including their genetic and physiological adaptations to aquatic life (Chikina *et al.*, 2016; Yuan *et al.*, 2021), they have typically focused on marine species, leaving freshwater species understudied. Overall, aquatic mammals have evolved a range of adaptations to suit their lifestyles in different environments (Berta *et al.*, 2015). Cetaceans, such as whales and dolphins, are fully aquatic and have streamlined bodies and flippers or flukes for propulsion. Depending on the species, they feed on a variety of organisms like fish, plankton, and other mammals. Sirenians, such as manatees, are also fully aquatic and have large, rounded bodies adapted for buoyancy. They feed on seagrasses and other aquatic plants and are found in shallow coastal waters and rivers. Pinnipeds, including seals and sea lions, are semi-aquatic and divide their time between land and sea, using their four flippers to swim and dive. Lutrinae, such as otters, are semi-aquatic and live in both freshwater and marine environments, using their streamlined bodies and webbed feet for swimming. The last two clades are carnivores and feeding on fish, crustaceans, and mollusks. 

Adaptations to their environments have shaped the different lifestyles of aquatic mammals, and identifying the common genetic elements that underlie these adaptations is a major challenge. Coping with hypoxic environments is a crucial adaptation that all aquatic mammals have evolved, enabling them to thrive in their habitats. For instance, cetaceans and sirenians experience hypoxia regularly throughout their lives, which has led many cetacean species to develop the ability to hold their breath for extended periods. This adaptation allows them to explore the depths of the ocean and remain submerged for extended periods (Ponganis, 2011). 

Similar selective pressures could potentially give rise to comparable molecular patterns at the molecular level. Therefore, the investigation of the HIF pathway genes in aquatic mammals holds significant importance in understanding their evolutionary adaptations and survival strategies in challenging environments. This is due to the pathway’s previously established significance at the cellular level. Our analysis of the natural selection regime revealed predominantly purifying selection on the coding regions that characterize these proteins, particularly as transcription factors, which correspond to highly conserved domains within these genes. Among all HIFα proteins, a common feature is the presence of an N-terminal bHLH (basic helix-loop-helix) domain located upstream of two PAS (Per-Arnt-Sim) domains. Mutations occurring in the bHLH and PAS domains are typically disruptive, leading to loss of function (Pamenter *et al.*, 2020). Thus, our findings of the pervasive effect of purifying selection on the HIF pathway genes align with prior research (Rytkönen *et al.*, 2011).

On the other hand, our analysis revealed that most of the sites detected under a positive selection regime suggest probable episodic selection events in different clades across the mammalian phylogeny. Notably, we found evidence pointing to a significant episodic event of positive selection in *HIF3A*, specifically a Glutamine residue at site 68 of HIF3*α*, which is present in all cetaceans, differing from the Arginine present in the rest of the phylogeny. While this represents a conservative replacement, whose effect is predicted to be neutral (Li *et al.*, 1984), our initial approximation approach to assess the structural effect of this variant and its potential impact on HIF3α folding and function using ΔΔG protein analysis, predicted a decrease in the stability of the HIF3α structure due to the Arg68Gln change.

HIF3α position 68 is located within the bHLH domain, composed mainly of basic amino acid residues, such as Arginine, that facilitate binding to the DNA phosphate backbone. The change to glutamine, which is a charge-neutral amino acid, may alter the electrostatic potential of the bHLH domain, likely decreasing the affinity to DNA. HIF3α is a transcription factor involved in the cellular response to hypoxia or low oxygen levels, although its precise role is not yet fully understood. Some studies have suggested that HIF3α may act as a negative regulator of the *HIF1A* pathway (Suzuki *et al.*, 2017; Cai *et al.*, 2020), which is critical for oxygen homeostasis in cells. Based on the above, we cannot predict or propose the effect of this variant on the cetacean clade. Thus, further functional studies *in silico* or *in vitro* will be necessary to evaluate in detail the structural role of this change and its adaptive effect in cetaceans to hypoxic environments.

On the other hand, our study differs from Zhu *et al.* (2018) in several aspects, such as the number and selection of specific sequences used, as well as the number of genes analyzed. Despite these discrepancies, both studies reached a similar conclusion that positively selected sites are located in the same regions within the proteins and that purifying selection had a significant role in the evolution of HIF pathway genes. We confirm that the 140S amino acid of EGLN3 is exclusive to cetaceans, although the protein stability analysis based on ΔΔG did not predict any stability changes.

### Deep-diving species

The depth and duration of immersion attained by aquatic mammals are closely linked to their diving capacity (Panneton, 2013). Previous research has shown that positive selection in certain genes is linked to diving abilities in cetaceans (Tian *et al.*, 2016; Tian *et al.*, 2021). We proposed that this hypothesis can be extended to other genes and taxonomic clades. For instance, certain representatives of Ziphiidae and Monodontidae (both cetacean families) and the genus *Mirounga* sp. (Pinnipeda) included in this study exhibit extreme deep-diving foraging behaviors, reaching depths of over 1000 m. The most extreme deep-diver is the beaked whale *Ziphius cavirostris*, which holds the record among mammals, reaching a depth of at least 2992 m and lasting 137.5 minutes (Schorr *et al.*, 2014). Yuan *et al.* (2021) found evidence of multiple genes related to HIF-α were under positive selection or had unique amino acid substitutions in cetacean deep-diving species. Refining our analysis of the HIF pathway genes, we did not find any associations of deep diving in cetaceans. However, we did observe statistically significant dN/dS values on the *ARNT2* gene in pinnipeds, particularly in the deep-diving Phocidae branch, as compared to shallow-diving species. These findings suggest that the common ancestor of Phocidae species may have already possessed this selective trait, possibly indicating their ability for deep-diving.

### Phenotypic pattern convergence

The emergence of similar phenotypes in different species may suggest shared evolutionary paths, however, these similarities may also occur in phylogenetically distant species through convergent evolution, as seen in the emergence of hypoxia tolerance in aquatic and subterranean mammals. At the molecular level, parallel molecular evolution refers to independent substitutions at a site resulting in the same derived amino acid in different species, while convergent substitutions refer to independent changes resulting in the same derived amino acid from different ancestral amino acids (Zhang and Kumar, 1997; Sackton and Clark, 2019).

There are various strategies for investigating convergent evolution, and we utilized the “Profile Change with One Change” (PCOC) method proposed by Rey *et al.* (2018). This method compares species with a convergent phenotype to closely related species with a different ancestral phenotype to identify genetic signatures responsible for convergent evolution. The method analyses changes in nucleotide or amino acid positions specifically in the branches where the convergent phenotype emerged. The same amino acid does not need to be present in species with similar adaptive traits, as the convergent evolution responsible for genetic changes may involve different amino acids that perform a similar functional role.

Using the PCOC method, we identified two convergent sites: site 485 in ARNT2 and site 364 in HIF3α. Analysis of ARNT2 amino acids in branches corresponding to Pinnipeda, *Trichechus manatus*, and Cetacea revealed the presence of threonine and serine, both having uncharged polar side chains, unlike the hydrophobic alanine present in the remaining phylogeny. Meanwhile, the side chains of amino acids at site 364 in HIF3α were classified as hydrophobic, with the valine amino acid (V) found in most aquatic mammals, isoleucine (I) in otariids (Pinnipedia), and leucine (L) in *Heterocephalus glaber*. The positive selection detected at the HIF3α 364 site by three methods further supports its possible role in convergence among aquatic mammalian lineages and this subterranean rodent species under hypoxia tolerance scenarios.

It should be noted that the inclusion of a broad range of genomes allows for a more comprehensive analysis of the HIF pathway genes involved in hypoxia tolerance. A larger number of species analyzed reduces the probability of inferring molecular convergence between species of aquatic mammals (Thomas *et al.*, 2017). This is because changes or alleles that are observed in only a few species within small datasets may not be exclusive to these groups, creating a sampling issue. Such variants may be present in a larger number of samples or species, making it, at least, uncertain to associate specific variants with adaptive phenotypes. By expanding the range of genomes analyzed, this study aimed to enhance its power to analyze and interpret data from aquatic mammals species. The inclusion of a broad range of genomes enables a more robust analysis of the HIF pathway genes underlying hypoxia tolerance. As an example, the functional characterization of *HIF1A* variants by Bi *et al.* (2015) was claimed to be exclusive to three cetacean species. However, a broader comparison of this site that includes a greater number of mammal species is enough to disprove this claim.

### Regulation mechanisms and hypoxia tolerance

The HIF pathway genes may be involved in various levels of cellular regulation related to hypoxia control. Our study focused solely on analyzing their respective coding sequences and examining how they exhibit selective regimes associated with different capacities for hypoxia tolerance. However, the cellular control of hypoxia may also involve the participation of these genes at other levels of regulation. Hindle (2020) has pointed out that gene regulatory mechanisms affecting the abundance or timing of gene expression could be responsible for physiological differences observed in marine mammal species. This is consistent with various previous studies that indicate that the *HIF* gene pathway is not “enriched” at the genome level in analyses of convergent phenotypes among marine mammals (Chikina *et al.*, 2016; Yuan *et al.*, 2021).

Therefore, it is possible that regulatory elements of gene expression at the *cis* or *trans* level, such as miRNAs, play a crucial role in the precise and fine-tune regulation of the hypoxic response in aquatic mammals. Several studies have highlighted the importance of “master controllers’’ of miRNAs in physiological regulation and adaptation to hypoxia (Hadj-Moussa *et al.*, 2022). For instance, miR-204 was found to downregulate the *VEGF* in Nile tilapia, a fish species, by directly acting on its 3′-UTR (Zhao *et al.*, 2014). Another research demonstrated that during hypoxic events, the upregulation of miR-24, which inhibits apoptosis, combined with the downregulation of miR-210, an indirect stabilizer of HIF1, could potentially promote neuronal preservation and trigger an adaptive hypoxic response in naked mole rats (Logan *et al.* 2020). In the case of aquatic mammals, Penso-Dolfin *et al.* (2020) investigated the role of miRNAs in the adaptive evolution of diving capacities in Weddell seals (*Leptonychotes weddelli*). Through differential expression analysis, they identified potential protective mechanisms in individual tissues, particularly relevant to hypoxia tolerance and anti-apoptotic pathways. Further studies are required to comprehend the role of these molecules in the overall adaptation of mammals to the aquatic environment.

## Conclusions

Our investigation of the HIF pathway genes in aquatic mammals attempted to shed light on the molecular adaptations that these animals have undergone to survive in hypoxic environments. The analysis of the natural selection regime revealed predominantly purifying selection on the coding regions that characterize these proteins, which correspond to highly conserved domains within these genes. However, we also found evidence of positive selection events in different clades across the mammalian phylogeny. We suggest that relaxed selection could have played a significant role in the evolutionary trajectory of HIF pathway genes in cetaceans. Notably, we identified an important episodic event of positive selection in *HIF3A*, which is present in all cetaceans. Further studies are needed to better understand the functional implications of these genetic changes and their role in the adaptation of aquatic mammals to their environment. We conclude that there is no unique “adaptive signature” in the evolution of diverse mammalian groups facing comparable selective pressures and that analyzing coding sequences alone may not fully elucidate the complex molecular scenario of adaptive processes, but our research adds a valuable piece to this puzzle. We can illustrate this concept, comparing different genetic elements such as coding changes, gene regulation and other factors to chess pieces. So, the infinite combinations of pieces movements on the chess board would resemble the multitude of possible combinations of the elements that can arise in the evolution of various mammalian taxa under comparable selective pressures. 

## Supplementary material

The following online material is available for this article:

Table S1 - Hypoxia signaling pathway genes summary.

Table S2 - Species names and NCBI accession numbers for each sequence.

Table S3 - Coding sequences of phylogenetic closely related species used as references to mapping genomes without annotation.

Table S4 - Aquatic mammals classification according to diving ability.

Table S5 - Presence of relaxed selection and constraint on aquatic mammals.

Table S6 - Parameter estimates of BUSTED method for HIF pathway genes.

Table S7 - Selective pressure analyses of HIF pathway genes by PAML site model method.

Table S8 - Positively selected sites from FUBAR and MEME.

Table S9 - FUBAR evidence of site purifying selection.

Table S10 - Nested Branch-level model.

Figure S1 - HIF signaling pathway (Kegg: ko04066).

Figure S2 - Molecular visualization of mutations (sites 68 and 437) in the AlphaFold three-dimensional model of HIF3α.

Figure S3 - Molecular visualization of mutation (site 195) in the AlphaFold three-dimensional model of VHL.
