# Supplementary material for: Adaptive strategies of aquatic mammals: Exploring the role of the HIF pathway and hypoxia tolerance
Source: Genet Mol Biol. 2024 Jan 19;46(3 Suppl 1):e20230140. doi: 10.1590/1678-4685-GMB-2023-0140 (PMC10802827; doi:10.1590/1678-4685-GMB-2023-0140)
Supplement: Table S1 - [file 1415-4757-GMB-46-03-s1-e20230140-s1.pdf]

Genetics and Molecular Biology
https://doi.org/10.1590/1678-4685-GMB-2023-0140

Supplementary Material to "Adaptive strategies of aquatic mammals: Exploring the role of the HIF pathway and hypoxia tolerance"

Table S1 - Hypoxia signaling pathway genes summary.

Gene	Name	GeneCards Identifier	NCBI Entrez Gene Identifier	Corresponding Uniprot Protein Identifier	NCBI Entrez Gene Summary
ARNT	Aryl Hydrocarbon Receptor Nuclear Translocator	GC01M150809	405	P27540	This gene encodes a protein containing a basic helix-loop-helix domain and two characteristic PAS domains along with a PAC domain. The encoded protein binds to ligand-bound aryl hydrocarbon receptor and aids in the movement of this complex to the nucleus, where it promotes the expression of genes involved in xenobiotic metabolism. This protein is also a co-factor for transcriptional regulation by hypoxia-inducible factor 1. Chromosomal translocation of this locus with the ETV6 (ets variant 6) gene on chromosome 12 have been described in leukemias. Alternative splicing results in multiple transcript variants.
ARNT2	Aryl Hydrocarbon Receptor Nuclear Translocator 2	GC15P080404	9915	Q9HBZ2	This gene encodes a member of the basic-helix-loop-helix-Per-Arnt-Sim (bHLH-PAS) superfamily of transcription factors. The encoded protein acts as a partner for several sensor proteins of the bHLH-PAS family, forming heterodimers with the sensor proteins that bind regulatory DNA sequences in genes responsive to developmental and environmental stimuli. Under hypoxic conditions, the encoded protein complexes with hypoxia-inducible factor 1alpha in the nucleus and this complex binds to hypoxia-responsive elements in enhancers and promoters of oxygen-responsive genes. A highly similar protein in mouse forms functional complexes with both aryl hydrocarbon receptors and Single-minded proteins, suggesting additional roles for the encoded protein in the metabolism of xenobiotic compounds and the regulation of neurogenesis, respectively.
EGLN1	Egl-9 Family Hypoxia Inducible Factor 1	GC01M231363	54583	Q91YE3	The protein encoded by this gene catalyzes the post-translational formation of 4-hydroxyproline in hypoxia-inducible factor (HIF) alpha proteins. HIF is a transcriptional complex that plays a central role in mammalian oxygen homeostasis. This protein functions as a cellular oxygen sensor, and under normal oxygen concentration, modification by prolyl hydroxylation is a key regulatory event that targets HIF subunits for proteasomal destruction via the von Hippel-Lindau ubiquitylation complex. Mutations in this gene are associated with erythrocytosis familial type 3 (ECYT3).

Gene	Name	GeneCards Identifier	NCBI Entrez Gene Identifier	Corresponding Uniprot Protein Identifier	NCBI Entrez Gene Summary
EGLN2	Egl-9 Family Hypoxia Inducible Factor 2	GC19P076239	112398	Q96KS0	The hypoxia inducible factor (HIF) is a transcriptional complex that is involved in oxygen homeostasis. At normal oxygen levels, the alpha subunit of HIF is targeted for degration by prolyl hydroxylation. This gene encodes an enzyme responsible for this post-translational modification. Alternative splicing results in multiple transcript variants. Read-through transcription also exists between this gene and the upstream RAB4B (RAB4B, member RAS oncogene family) gene.
EGLN3	Egl-9 Family Hypoxia Inducible Factor 3	GC14M033924	112399	Q9H6Z9	Enables peptidyl-proline 4-dioxygenase activity. Involved in several processes, including activation of cysteine-type endopeptidase activity involved in apoptotic process; peptidyl-proline hydroxylation to 4-hydroxy-L-proline; and response to hypoxia. Located in cytosol and nucleus. Implicated in renal cell carcinoma. Biomarker of clear cell renal cell carcinoma.
EPAS1	Endothelial PAS Domain Protein 1	GC02P046293	2034	Q99814	This gene encodes a transcription factor involved in the induction of genes regulated by oxygen, which is induced as oxygen levels fall. The encoded protein contains a basic-helix-loop-helix domain protein dimerization domain as well as a domain found in proteins in signal transduction pathways which respond to oxygen levels. Mutations in this gene are associated with erythrocytosis familial type 4.
HIF1AN	Hypoxia Inducible Factor 1 Subunit Alpha Inhibitor	GC10P100529	55662	Q9NWT6	Enables several functions, including 2-oxoglutarate-dependent dioxygenase activity; NF-kappaB binding activity; and transition metal ion binding activity. Involved in several processes, including negative regulation of Notch signaling pathway; negative regulation of transcription from RNA polymerase II promoter in response to hypoxia; and protein hydroxylation. Located in cytosol; nucleoplasm; and perinuclear region of cytoplasm. Colocalizes with nucleus.
HIF1A	Hypoxia Inducible Factor 1 Subunit Alpha	GC14P061695	3091	Q16665	This gene encodes the alpha subunit of transcription factor hypoxia-inducible factor-1 (HIF-1), which is a heterodimer composed of an alpha and a beta subunit. HIF-1 functions as a master regulator of cellular and systemic homeostatic response to hypoxia by activating transcription of many genes, including those involved in energy metabolism, angiogenesis, apoptosis, and other genes whose protein products increase oxygen delivery or facilitate metabolic adaptation to hypoxia. HIF-1 thus plays an essential role in embryonic vascularization, tumor angiogenesis and pathophysiology of ischemic disease. Alternatively spliced transcript variants encoding different isoforms have been identified for this gene.
HIF3α	Hypoxia Inducible Factor 3 Subunit Alpha	GC19P046297	64344	Q9Y2N7	The protein encoded by this gene is the alpha-3 subunit of one of several alpha/beta-subunit heterodimeric transcription factors that regulate many adaptive responses to low oxygen tension (hypoxia). The alpha-3 subunit lacks the transactivation domain found in factors containing either the alpha-1 or alpha-2 subunits. It is thought that factors containing the alpha-3 subunit are negative regulators of hypoxia-inducible gene expression. Multiple alternatively spliced transcript variants have been found for this gene.

Gene	Name	GeneCards Identifier	NCBI Entrez Gene Identifier	Corresponding Uniprot Protein Identifier	NCBI Entrez Gene Summary
VEGFA	Vascular Endothelial Growth Factor A	GC06P043770	7422	P15692	This gene is a member of the PDGF/VEGF growth factor family. It encodes a heparin-binding protein, which exists as a disulfide-linked homodimer. This growth factor induces proliferation and migration of vascular endothelial cells, and is essential for both physiological and pathological angiogenesis. Disruption of this gene in mice resulted in abnormal embryonic blood vessel formation. This gene is upregulated in many known tumors and its expression is correlated with tumor stage and progression. Elevated levels of this protein are found in patients with POEMS syndrome, also known as Crow-Fukase syndrome. Allelic variants of this gene have been associated with microvascular complications of diabetes 1 (MVCD1) and atherosclerosis. Alternatively spliced transcript variants encoding different isoforms have been described. There is also evidence for alternative translation initiation from upstream non-AUG (CUG) codons resulting in additional isoforms. A recent study showed that a C-terminally extended isoform is produced by use of an alternative in-frame translation termination codon via a stop codon readthrough mechanism, and that this isoform is antiangiogenic. Expression of some isoforms derived from the AUG start codon is regulated by a small upstream open reading frame, which is located within an internal ribosome entry site. The levels of VEGF are increased during infection with severe acute respiratory syndrome coronavirus 2 (SARS-CoV-2), thus promoting inflammation by facilitating recruitment of inflammatory cells, and by increasing the level of angiopoietin II (Ang II), one of two products of the SARS-CoV-2 binding target, angiotensin-converting enzyme 2 (ACE2). In turn, Ang II facilitates the elevation of VEGF, thus forming a vicious cycle in the release of inflammatory cytokines.
VHL	Von Hippel-Lindau Tumor Suppressor	GC03P013997	7428	P40337	This gene encodes a component of an ubiquitination complex. The encoded protein is involved in the ubiquitination and degradation of hypoxia-inducible-factor (HIF), which is a transcription factor that plays a central role in the regulation of gene expression by oxygen. In addition to oxygen-related gene expression, this protein plays a role in many other cellular processes including cilia formation, cytokine signaling, regulation of senescence, and formation of the extracellular matrix. Variants of this gene are associated with von Hippel-Lindau syndrome, pheochromocytoma, erythrocytosis, renal cell carcinoma, and cerebellar hemangioblastoma.
