# Supplementary material for: Adaptive strategies of aquatic mammals: Exploring the role of the HIF pathway and hypoxia tolerance
Source: Genet Mol Biol. 2024 Jan 19;46(3 Suppl 1):e20230140. doi: 10.1590/1678-4685-GMB-2023-0140 (PMC10802827; doi:10.1590/1678-4685-GMB-2023-0140)
Supplement: Table S2 - [file 1415-4757-GMB-46-03-s1-e20230140-s2.pdf]

Genetics and Molecular Biology
https://doi.org/10.1590/1678-4685-GMB-2023-0140

Supplementary Material to "Adaptive strategies of aquatic mammals: Exploring the role of the HIF pathway and hypoxia tolerance"

Table S2 - Species names and NCBI accession numbers for each sequence.

Gene ID	Gene symbol	Scientific name	Common name	RefSeq Transcript accessions	RefSeq Protein accessions
			VEGFA		
101279969	*VEGFA*	*Orcinus orca*	Killer whale	XM_004267535.1	XP_004267583.1
101325765	*VEGFA*	*Tursiops truncatus*	Common bottlenose dolphin	XM_033864164.1	XP_033720055.1
102981739	*VEGFA*	*Physeter catodon*	Sperm whale	XM_024121779.2	XP_023977547.1
103008131	*VEGFA*	*Balaenoptera acutorostrata scammoni*	Minke whale	XM_028161780.1	XP_028017581.1
103089095	*VEGFA*	*Lipotes vexillifer*	Yangtze River dolphin	XM_007458277.1	XP_007458339.1
111178866	*VEGFA*	*Delphinapterus leucas*	Beluga whale	XM_022582006.2	XP_022437714.1
112413893	*VEGFA*	*Neophocaena asiaeorientalis asiaeorientalis*	Yangtze finless porpoise	XM_024766066.1	XP_024621834.1
113631159	*VEGFA*	*Lagenorhynchus obliquidens*	Pacific white-sided dolphin	XM_027127376.1	XP_026983177.1
114907021	*VEGFA*	*Monodon monoceros*	Narwhal	XM_029242118.1	XP_029097951.1

Gene ID	Gene symbol	Scientific name	Common name	RefSeq Transcript accessions	RefSeq Protein accessions
115860649	*VEGFA*	*Globicephala melas*	Long-finned pilot whale	XM_030870595.1	XP_030726455.1
116762165	*VEGFA*	*Phocoena sinus*	Vaquita	XM_032648915.1	XP_032504806.1
118903198	*VEGFA*	*Balaenoptera musculus*	Blue whale	XM_036868005.1	XP_036723900.1
101363442	*VEGFA*	*Odobenus rosmarus divergens*	Pacific walrus	XM_012565115.1	XP_012420569.1
110581991	*VEGFA*	*Neomonachus schauinslandi*	Hawaiian monk seal	XM_021691925.1	XP_021547600.1
111147726	*LOC111147726*	*Enhydra lutris kenyoni*	Sea otter	XM_022503948.1	XP_022359656.1
112837827	*VEGFA*	*Callorhinus ursinus*	Northern fur seal	XM_025890397.1	XP_025746182.1
113927959	*VEGFA*	*Zalophus californianus*	California sea lion	XM_027604069.1	XP_027459870.1
114204633	*VEGFA*	*Eumetopias jubatus*	Steller sea lion	XM_028097850.1	XP_027953651.1
116635796	*VEGFA*	*Phoca vitulina*	Harbor seal	XM_032410104.1	XP_032265995.1
116870341	*VEGFA*	*Lontra canadensis*	Northern American river otter	XM_032861948.1	XP_032717839.1
118001628	*VEGFA*	*Mirounga leonina*	Southern elephant seal	XM_034991882.1	XP_034847773.1
101341920	*LOC101341920*	*Trichechus manatus latirostris*	Florida manatee	XM_023734117.1	XP_023589885.1
7422	*VEGFA*	*Homo sapiens*	Human	NM_001171623.2	NP_001165094.1
403802	*VEGFA*	*Canis lupus familiaris*	Dog	NM_001003175.2	NP_001003175.2
101675945	*VEGFA*	*Mustela putorius furo*	Domestic ferret	XM_004740321.2	XP_004740378.1
113261040	*VEGFA*	*Ursus arctos horribilis*	Grizzly bear	XM_026506965.1	XP_026362750.1
112862794	*VEGFA*	*Puma concolor*	Puma	XM_025926021.1	XP_025781806.1
22339	*Vegfa*	*Mus musculus*	House mouse	NM_001287056.1	NP_001273985.1

Gene ID	Gene symbol	Scientific name	Common name	RefSeq Transcript accessions	RefSeq Protein accessions
281572	*VEGFA*	*Bos taurus*	Cattle	NM_001316956.1	NP_001303885.1
397157	*VEGFA*	*Sus scrofa*	Pig	NM_214084.1	NP_999249.1
574209	*VEGFA*	*Macaca mulatta*	Rhesus monkey	NM_001278384.1	NP_001265313.1
100008899	*VEGFA*	*Oryctolagus cuniculus*	Rabbit	XM_017345155.1	XP_017200644.1
100033839	*VEGFA*	*Equus caballus*	Horse	XM_023624005.1	XP_023479773.1
100389629	*VEGFA*	*Callithrix jacchus*	White-tufted-ear marmoset	XM_003732674.3	XP_003732722.1
100661392	*VEGFA*	*Loxodonta africana*	African savanna elephant	XM_010587424.2	XP_010585726.1
100860957	*VEGFA*	*Capra hircus*	Goat	NM_001287034.1	NP_001273963.1
101131949	*VEGFA*	*Gorilla gorilla*	Western gorilla	XM_004044089.3	XP_004044137.1
101418722	*VEGFA*	*Dasypus novemcinctus*	Nine-banded armadillo	XM_004473109.3	XP_004473166.2
101702403	*Vegfa*	*Heterocephalus glaber*	Naked mole-rat	XM_013072755.2	XP_012928209.1
102254357	*VEGFA*	*Myotis brandtii*	Brandt's bat	XM_014535375.1	XP_014390861.1
102396324	*VEGFA*	*Bubalus bubalis*	Water buffalo	XM_025263754.1	XP_025119539.1
102514843	*VEGFA*	*Camelus ferus*	Wild Bactrian camel	XM_032463522.1	XP_032319413.1
102536403	*VEGFA*	*Vicugna pacos*	Alpaca	XM_031688589.1	XP_031544449.1
102829023	*VEGFA*	*Chrysochloris asiatica*	Cape golden mole	XM_006860503.1	XP_006860565.1
103193092	*VEGFA*	*Orycteropus afer afer*	Aardvark	XM_007936195.1	XP_007934386.1
103296626	*VEGFA*	*Eptesicus fuscus*	Big brown bat	XM_008153240.2	XP_008151462.1
105088633	*VEGFA*	*Camelus dromedarius*	Arabian camel	XM_010979532.2	XP_010977834.2

Gene ID	Gene symbol	Scientific name	Common name	RefSeq Transcript accessions	RefSeq Protein accessions
108407135	*VEGFA*	*Manis javanica*	Malayan pangolin	XM_017676262.2	XP_017531751.1
109682127	*Vegfa*	*Castor canadensis*	American beaver	XM_020157195.1	XP_020012784.1
110201281	*VEGFA*	*Phascolarctos cinereus*	Koala	XM_020976813.1	XP_020832472.1
			EGLN3		
101284894	*EGLN3*	*Orcinus orca*	Killer whale	XM_004285704.2	XP_004285752.1
101332738	*EGLN3*	*Tursiops truncatus*	Common bottlenose dolphin	XM_019924248.1	XP_019779807.1
102988280	*EGLN3*	*Physeter catodon*	Sperm whale	XM_007117882.3	XP_007117944.1
102997608	*EGLN3*	*Balaenoptera acutorostrata scammoni*	Minke whale	XM_007180697.1	XP_007180759.1
103088451	*EGLN3*	*Lipotes vexillifer*	Yangtze River dolphin	XM_007453028.1	XP_007453090.1
111173240	*EGLN3*	*Delphinapterus leucas*	Beluga whale	XM_022570737.2	XP_022426445.1
112397732	*EGLN3*	*Neophocaena asiaeorientalis asiaeorientalis*	Yangtze finless porpoise	XM_024741994.1	XP_024597762.1
113622658	*EGLN3*	*Lagenorhynchus obliquidens*	Pacific white-sided dolphin	XM_027111038.1	XP_026966839.1
114904900	*EGLN3*	*Monodon monoceros*	Narwhal	XM_029239297.1	XP_029095130.1
115852202	*EGLN3*	*Globicephala melas*	Long-finned pilot whale	XM_030854768.1	XP_030710628.1
101367839	*EGLN3*	*Odobenus rosmarus divergens*	Pacific walrus	XM_004399963.2	XP_004400020.1
102748129	*EGLN3*	*Leptonychotes weddellii*	Weddell seal	XM_006748618.2	XP_006748681.1
110585605	*EGLN3*	*Neomonachus schauinslandi*	Hawaiian monk seal	XM_021695781.1	XP_021551456.1
112822490	*EGLN3*	*Callorhinus ursinus*	Northern fur seal	XM_025870188.1	XP_025725973.1
113909953	*EGLN3*	*Zalophus californianus*	California sea lion	XM_027571653.1	XP_027427454.1

Gene ID	Gene symbol	Scientific name	Common name	RefSeq Transcript accessions	RefSeq Protein accessions
114217792	*EGLN3*	*Eumetopias jubatus*	Steller sea lion	XM_028114736.1	XP_027970537.1
116632890	*EGLN3*	*Phoca vitulina*	Harbor seal	XM_032404930.1	XP_032260821.1
111160932	*LOC111160932*	*Enhydra lutris kenyoni*	Sea otter	XM_022524334.1	XP_022380042.1
101359457	*LOC101359457*	*Trichechus manatus latirostris*	Florida manatee	XM_004376525.2	XP_004376582.1
112399	*EGLN3*	*Homo sapiens*	Human	NM_022073.4	NP_071356.1
103107985	*EGLN3*	*Erinaceus europaeus*	Western European hedgehog	XM_007516824.2	XP_007516886.1
112856377	*EGLN3*	*Puma concolor*	Puma	XM_025919953.1	XP_025775738.1
112407	*Egln3*	*Mus musculus*	House mouse	NM_028133.2	NP_082409.2
480286	*EGLN3*	*Canis lupus familiaris*	Dog	XM_537408.5	XP_537408.3
535578	*EGLN3*	*Bos taurus*	Cattle	NM_001101164.2	NP_001094634.1
717342	*EGLN3*	*Macaca mulatta*	Rhesus monkey	NM_001194425.2	NP_001181354.1
100056635	*EGLN3*	*Equus caballus*	Horse	XM_003363671.4	XP_003363719.1
100152368	*EGLN3*	*Sus scrofa*	Pig	XM_001928507.6	XP_001928542.3
100354210	*EGLN3*	*Oryctolagus cuniculus*	Rabbit	XM_017348255.1	XP_017203744.1
100389821	*EGLN3*	*Callithrix jacchus*	White-tufted-ear marmoset	XM_002753799.4	XP_002753845.1
100674039	*EGLN3*	*Loxodonta africana*	African savanna elephant	XM_003408771.3	XP_003408819.1
101150306	*EGLN3*	*Gorilla gorilla*	Western gorilla	XM_004055066.3	XP_004055114.1
101447775	*EGLN3*	*Dasypus novemcinctus*	Nine-banded armadillo	XM_004468980.2	XP_004469037.1
101675598	*EGLN3*	*Mustela putorius furo*	Domestic ferret	XM_004775747.2	XP_004775804.1

Gene ID	Gene symbol	Scientific name	Common name	RefSeq Transcript accessions	RefSeq Protein accessions
102179524	*EGLN3*	*Capra hircus*	Goat	XM_005695224.3	XP_005695281.1
102261310	*EGLN3*	*Myotis brandtii*	Brandt's bat	XM_005874570.2	XP_005874632.1
102402360	*EGLN3*	*Bubalus bubalis*	Water buffalo	XM_006056954.2	XP_006057016.1
102516321	*EGLN3*	*Camelus ferus*	Wild Bactrian camel	XM_014564812.2	XP_014420298.1
102537733	*EGLN3*	*Vicugna pacos*	Alpaca	XM_006207565.3	XP_006207627.1
102816417	*EGLN3*	*Chrysochloris asiatica*	Cape golden mole	XM_006835359.1	XP_006835422.1
103195079	*EGLN3*	*Orycteropus afer afer*	Aardvark	XM_007938537.1	XP_007936728.1
103680905	*EGLN3*	*Ursus maritimus*	Polar bear	XM_008710423.1	XP_008708645.1
105094177	*EGLN3*	*Camelus dromedarius*	Arabian camel	XM_010986147.2	XP_010984449.1
108387980	*EGLN3*	*Manis javanica*	Malayan pangolin	XM_017646358.1	XP_017501847.1
110223697	*EGLN3*	*Phascolarctos cinereus*	Koala	XM_021009363.1	XP_020865022.1
113266114	*EGLN3*	*Ursus arctos horribilis*	Grizzly bear	XM_026513575.1	XP_026369360.1
114088222	*Egln3*	*Marmota flaviventris*	Yellow-bellied marmot	XM_027929811.1	XP_027785612.1
103300303	*EGLN3*	*Eptesicus fuscus*	Big brown bat	XM_008157186.2	XP_008155408.1
101718787	*Egln3*	*Heterocephalus glaber*	Naked mole-rat	XM_004867577.3	XP_004867634.1
104868693	*Egln3*	*Fukomys damarensis*	Damara mole-rat	XM_010633595.1	XP_010631897.1
103738632	*Egln3*	*Nannospalax galili*	Upper Galilee mountains blind mole-rat	XM_010633595.1	XP_010631897.1
116749704	*EGLN3*	*Phocoena sinus*	Vaquita	XM_032624365.1	XP_032480256.1
109697177	*Egln3*	*Castor canadensis*	American beaver	XM_020180612.1	XP_020036201.1

Gene ID	Gene symbol	Scientific name	Common name	RefSeq Transcript accessions	RefSeq Protein accessions
			EPAS1		
101276152	*EPAS1*	*Orcinus orca*	Killer whale	XM_004264992.1	XP_004265040.1
101324698	*EPAS1*	*Tursiops truncatus*	Common bottlenose dolphin	XM_019942458.1	XP_019798017.1
102989059	*EPAS1*	*Physeter catodon*	Sperm whale	XM_007105118.3	XP_007105180.2
103005217	*EPAS1*	*Balaenoptera acutorostrata scammoni*	Minke whale	XM_007190076.2	XP_007190138.1
103084980	*EPAS1*	*Lipotes vexillifer*	Yangtze River dolphin	XM_007459966.1	XP_007460028.1
111174799	*EPAS1*	*Delphinapterus leucas*	Beluga whale	XM_022573921.2	XP_022429629.1
112405266	*EPAS1*	*Neophocaena asiaeorientalis asiaeorientalis*	Yangtze finless porpoise	XM_024753625.1	XP_024609393.1
113623755	*EPAS1*	*Lagenorhynchus obliquidens*	Pacific white-sided dolphin	XM_027113201.1	XP_026969002.1
114900369	*EPAS1*	*Monodon monoceros*	Narwhal	XM_029231443.1	XP_029087276.1
115854047	*EPAS1*	*Globicephala melas*	Long-finned pilot whale	XM_030857763.1	XP_030713623.1
101362839	*EPAS1*	*Odobenus rosmarus divergens*	Pacific walrus	XM_004403370.2	XP_004403427.1
102748984	*EPAS1*	*Leptonychotes weddellii*	Weddell seal	XM_006740602.2	XP_006740665.1
110590488	*EPAS1*	*Neomonachus schauinslandi*	Hawaiian monk seal	XM_021701296.1	XP_021556971.1
112821614	*EPAS1*	*Callorhinus ursinus*	Northern fur seal	XM_025868895.1	XP_025724680.1
113937131	*EPAS1*	*Zalophus californianus*	California sea lion	XM_027620870.1	XP_027476671.1
114209788	*EPAS1*	*Eumetopias jubatus*	Steller sea lion	XM_028103997.1	XP_027959798.1
116636329	*EPAS1*	*Phoca vitulina*	Harbor seal	XM_032411052.1	XP_032266943.1
111155870	*LOC111155870*	*Enhydra lutris kenyoni*	Sea otter	XM_022516312.1	XP_022372020.1

Gene ID	Gene symbol	Scientific name	Common name	RefSeq Transcript accessions	RefSeq Protein accessions
101356007	*LOC101356007*	*Trichechus manatus latirostris*	Florida manatee	XM_023734703.1	XP_023590471.1
2034	*EPAS1*	*Homo sapiens*	Human	NM_001430.5	NP_001421.2
714641	*EPAS1*	*Macaca mulatta*	Rhesus monkey	XM_015112282.2	XP_014967768.1
100068622	*EPAS1*	*Equus caballus*	Horse	XM_005600005.3	XP_005600062.1
100655103	*EPAS1*	*Loxodonta africana*	African savanna elephant	XM_010597282.2	XP_010595584.1
13819	*Epas1*	*Mus musculus*	House mouse	NM_010137.3	NP_034267.3
474578	*EPAS1*	*Canis lupus familiaris*	Dog	XM_005626080.3	XP_005626137.1
282711	*EPAS1*	*Bos taurus*	Cattle	NM_174725.2	NP_777150.1
100037272	*EPAS1*	*Sus scrofa*	Pig	NM_001097420.1	NP_001090889.1
100348809	*EPAS1*	*Oryctolagus cuniculus*	Rabbit	XM_008254453.2	XP_008252675.1
100396711	*EPAS1*	*Callithrix jacchus*	White-tufted-ear marmoset	XM_002757798.3	XP_002757844.1
101411190	*EPAS1*	*Dasypus novemcinctus*	Nine-banded armadillo	XM_004447332.1	XP_004447389.1
101679355	*EPAS1*	*Mustela putorius furo*	Domestic ferret	XM_004752997.2	XP_004753054.1
102180230	*EPAS1*	*Capra hircus*	Goat	XM_018055188.1	XP_017910677.1
102257697	*EPAS1*	*Myotis brandtii*	Brandt's bat	XM_014538497.1	XP_014393983.1
102405150	*EPAS1*	*Bubalus bubalis*	Water buffalo	XM_006054785.2	XP_006054847.1
102517299	*EPAS1*	*Camelus ferus*	Wild Bactrian camel	XM_032497440.1	XP_032353331.1
102533714	*EPAS1*	*Vicugna pacos*	Alpaca	XM_031672613.1	XP_031528473.1
102820466	*EPAS1*	*Chrysochloris asiatica*	Cape golden mole	XM_006839419.1	XP_006839482.1

Gene ID	Gene symbol	Scientific name	Common name	RefSeq Transcript accessions	RefSeq Protein accessions
103113794	*EPAS1*	*Erinaceus europaeus*	Western European hedgehog	XM_016188251.1	XP_016043737.1
103200007	*EPAS1*	*Orycteropus afer afer*	Aardvark	XM_007944420.1	XP_007942611.1
103671227	*EPAS1*	*Ursus maritimus*	Polar bear	XM_008699825.1	XP_008698047.1
105103582	*EPAS1*	*Camelus dromedarius*	Arabian camel	XM_031466944.1	XP_031322804.1
108406403	*EPAS1*	*Manis javanica*	Malayan pangolin	XM_017675100.1	XP_017530589.1
109025170	*EPAS1*	*Gorilla gorilla*	Western gorilla	XM_031006928.1	XP_030862788.1
110218486	*EPAS1*	*Phascolarctos cinereus*	Koala	XM_021001228.1	XP_020856887.1
113246141	*EPAS1*	*Ursus arctos horribilis*	Grizzly bear	XM_026486635.1	XP_026342420.1
114092033	*Epas1*	*Marmota flaviventris*	Yellow-bellied marmot	XM_027934586.1	XP_027790387.1
118008175	*EPAS1*	*Mirounga leonina*	Southern elephant seal	XM_035001638.1	XP_034857529.1
116764537	*EPAS1*	*Phocoena sinus*	Vaquita	XM_032653240.1	XP_032509131.1
116856808	*EPAS1*	*Lontra canadensis*	Northern American river otter	XM_032840429.1	XP_032696320.1
103305653	*EPAS1*	*Eptesicus fuscus*	Big brown bat	XM_028139985.1	XP_027995786.1
101701434	*Epas1*	*Heterocephalus glaber*	Naked mole-rat	XM_004839394.2	XP_004839451.1
104856395	*Epas1*	*Fukomys damarensis*	Damara mole-rat	XM_010615775.3	XP_010614077.1
103725756	*Epas1*	*Nannospalax galili*	Upper Galilee mountains blind mole-rat	XM_008823068.3	XP_008821290.1
109698638	*EPAS1*	*Castor canadensis*	American beaver	XM_020182983.1	XP_020038572.1
118905932	*EPAS1*	*Balaenoptera musculus*	Blue whale	XM_036872950.1	XP_036728845.1
			HIF1A		

Gene ID	Gene symbol	Scientific name	Common name	RefSeq Transcript accessions	RefSeq Protein accessions
101271390	*HIF1A*	*Orcinus orca*	Killer whale	XM_004262104.1	XP_004262152.1
101324632	*HIF1A*	*Tursiops truncatus*	Common bottlenose dolphin	XM_019923093.1	XP_019778652.1
102978574	*HIF1A*	*Physeter catodon*	Sperm whale	XM_007109893.3	XP_007109955.2
103008124	*HIF1A*	*Balaenoptera acutorostrata scammoni*	Minke whale	XM_007192958.2	XP_007193020.1
103073387	*HIF1A*	*Lipotes vexillifer*	Yangtze River dolphin	XM_007471700.1	XP_007471762.1
111173087	*HIF1A*	*Delphinapterus leucas*	Beluga whale	XM_022570428.2	XP_022426136.1
112401826	*HIF1A*	*Neophocaena asiaeorientalis asiaeorientalis*	Yangtze finless porpoise	XM_024748345.1	XP_024604113.1
113622835	*HIF1A*	*Lagenorhynchus obliquidens*	Pacific white-sided dolphin	XM_027111435.1	XP_026967236.1
114904307	*HIF1A*	*Monodon monoceros*	Narwhal	XM_029238066.1	XP_029093899.1
115852370	*HIF1A*	*Globicephala melas*	Long-finned pilot whale	XM_030855168.1	XP_030711028.1
101374637	*HIF1A*	*Odobenus rosmarus divergens*	Pacific walrus	XM_004411877.2	XP_004411934.1
102738300	*HIF1A*	*Leptonychotes weddellii*	Weddell seal	XM_006738543.2	XP_006738606.1
110590734	*HIF1A*	*Neomonachus schauinslandi*	Hawaiian monk seal	XM_021701545.1	XP_021557220.1
112822321	*HIF1A*	*Callorhinus ursinus*	Northern fur seal	XM_025869920.1	XP_025725705.1
113908556	*HIF1A*	*Zalophus californianus*	California sea lion	XM_027568783.1	XP_027424584.1
114217524	*HIF1A*	*Eumetopias jubatus*	Steller sea lion	XM_028114429.1	XP_027970230.1
116633035	*HIF1A*	*Phoca vitulina*	Harbor seal	XM_032405250.1	XP_032261141.1
111159390	*HIF1A*	*Enhydra lutris kenyoni*	Sea otter	XM_022521812.1	XP_022377520.1
101347136	*LOC101347136*	*Trichechus manatus latirostris*	Florida manatee	XM_004370926.1	XP_004370983.1

Gene ID	Gene symbol	Scientific name	Common name	RefSeq Transcript accessions	RefSeq Protein accessions
118002352	*HIF1A*	*Mirounga leonina*	Southern elephant seal	XM_034992741.1	XP_034848632.1
116748866	*HIF1A*	*Phocoena sinus*	Vaquita	XM_032622389.1	XP_032478280.1
396696	*HIF1A*	*Sus scrofa*	Pig	NM_001123124.1	NP_001116596.1
704678	*HIF1A*	*Macaca mulatta*	Rhesus monkey	XM_015143834.2	XP_014999320.1
100061166	*HIF1A*	*Equus caballus*	Horse	XM_023627857.1	XP_023483625.1
100659617	*HIF1A*	*Loxodonta africana*	African savanna elephant	XM_010588680.2	XP_010586982.1
100861391	*HIF1A*	*Capra hircus*	Goat	NM_001285728.1	NP_001272657.1
101443806	*HIF1A*	*Dasypus novemcinctus*	Nine-banded armadillo	XM_004459739.3	XP_004459796.1
102245337	*HIF1A*	*Myotis brandtii*	Brandt's bat	XM_014545934.1	XP_014401420.1
102514077	*HIF1A*	*Camelus ferus*	Wild Bactrian camel	XM_032482145.1	XP_032338036.1
102532981	*HIF1A*	*Vicugna pacos*	Alpaca	XM_015249776.2	XP_015105262.1
102814449	*HIF1A*	*Chrysochloris asiatica*	Cape golden mole	XM_006864576.1	XP_006864638.1
103110113	*HIF1A*	*Erinaceus europaeus*	Western European hedgehog	XM_007519273.2	XP_007519335.1
103203990	*HIF1A*	*Orycteropus afer afer*	Aardvark	XM_007949088.1	XP_007947279.1
108404522	*HIF1A*	*Manis javanica*	Malayan pangolin	XM_017672141.1	XP_017527630.1
110208444	*HIF1A*	*Phascolarctos cinereus*	Koala	XM_020986497.1	XP_020842156.1
112855387	*HIF1A*	*Puma concolor*	Puma	XM_025919032.1	XP_025774817.1
114087954	*Hif1a*	*Marmota flaviventris*	Yellow-bellied marmot	XM_027929462.1	XP_027785263.1
103672464	*HIF1A*	*Ursus maritimus*	Polar bear	XM_008701170.1	XP_008699392.1

Gene ID	Gene symbol	Scientific name	Common name	RefSeq Transcript accessions	RefSeq Protein accessions
113242413	*HIF1A*	*Ursus arctos horribilis*	Grizzly bear	XM_026480436.1	XP_026336221.1
100009579	*HIF1A*	*Oryctolagus cuniculus*	Rabbit	NM_001082782.1	NP_001076251.1
15251	*Hif1a*	*Mus musculus*	House mouse	NM_001313919.1	NP_001300848.1
281814	*HIF1A*	*Bos taurus*	Cattle	XM_024997269.1	XP_024853037.1
100411678	*HIF1A*	*Callithrix jacchus*	White-tufted-ear marmoset	XM_035260663.1	XP_035116554.1
101136350	*HIF1A*	*Gorilla gorilla*	Western gorilla	XM_019009770.1	XP_018865315.1
480348	*HIF1A*	*Canis lupus familiaris*	Dog	NM_001287163.1	NP_001274092.1
102392717	*HIF1A*	*Bubalus bubalis*	Water buffalo	XM_025295405.1	XP_025151190.1
105086214	*HIF1A*	*Camelus dromedarius*	Arabian camel	XM_031453821.1	XP_031309681.1
101692645	*HIF1A*	*Mustela putorius furo*	Domestic ferret	XM_004738887.2	XP_004738944.1
116878068	*HIF1A*	*Lontra canadensis*	Northern American river otter	XM_032875250.1	XP_032731141.1
103283673	*HIF1A*	*Eptesicus fuscus*	Big brown bat	XM_028141623.1	XP_027997424.1
101715845	*Hif1a*	*Heterocephalus glaber*	Naked mole-rat	XM_004837433.3	XP_004837490.1
104849351	*Hif1a*	*Fukomys damarensis*	Damara mole-rat	XM_010605745.3	XP_010604047.2
103747967	*Hif1a*	*Nannospalax galili*	Upper Galilee mountains blind mole-rat	XM_029554756.1	XP_029410616.1
109682911	*HIF1A*	*Castor canadensis*	American beaver	XM_020158487.1	XP_020014076.1
			EGLN2		
101288571	*EGLN2*	*Orcinus orca*	Killer whale	XM_004271260.2	XP_004271308.1
102973467	*EGLN2*	*Physeter catodon*	Sperm whale	XM_024132665.2	XP_023988433.1

Gene ID	Gene symbol	Scientific name	Common name	RefSeq Transcript accessions	RefSeq Protein accessions
103012144	*EGLN2*	*Balaenoptera acutorostrata scammoni*	Minke whale	XM_007198492.2	XP_007198554.1
103078947	*EGLN2*	*Lipotes vexillifer*	Yangtze River dolphin	XM_007458630.1	XP_007458692.1
111180833	*EGLN2*	*Delphinapterus leucas*	Beluga whale	XM_022585601.2	XP_022441309.1
112415250	*EGLN2*	*Neophocaena asiaeorientalis asiaeorientalis*	Yangtze finless porpoise	XM_024768126.1	XP_024623894.1
113605897	*EGLN2*	*Lagenorhynchus obliquidens*	Pacific white-sided dolphin	XM_027079598.1	XP_026935399.1
114903924	*EGLN2*	*Monodon monoceros*	Narwhal	XM_029237154.1	XP_029092987.1
115862412	*EGLN2*	*Globicephala melas*	Long-finned pilot whale	XM_030874204.1	XP_030730064.1
101368021	*EGLN2*	*Odobenus rosmarus divergens*	Pacific walrus	XM_004396336.1	XP_004396393.1
102748492	*EGLN2*	*Leptonychotes weddellii*	Weddell seal	XM_006745454.2	XP_006745517.1
110590276	*EGLN2*	*Neomonachus schauinslandi*	Hawaiian monk seal	XM_021701043.1	XP_021556718.1
112810620	*EGLN2*	*Callorhinus ursinus*	Northern fur seal	XM_025854333.1	XP_025710118.1
113935977	*EGLN2*	*Zalophus californianus*	California sea lion	XM_027618721.1	XP_027474522.1
114202458	*EGLN2*	*Eumetopias jubatus*	Steller sea lion	XM_028095641.1	XP_027951442.1
116623371	*EGLN2*	*Phoca vitulina*	Harbor seal	XM_032390505.1	XP_032246396.1
111161431	*LOC111161431*	*Enhydra lutris kenyoni*	Sea otter	XM_022525065.1	XP_022380773.1
101351991	*LOC101351991*	*Trichechus manatus latirostris*	Florida manatee	XM_004390844.2	XP_004390901.1
112398	*EGLN2*	*Homo sapiens*	Human	NM_080732.4	NP_542770.2
100064859	*EGLN2*	*Equus caballus*	Horse	XM_023649776.1	XP_023505544.1
102191264	*EGLN2*	*Capra hircus*	Goat	XM_018062448.1	XP_017917937.1

Gene ID	Gene symbol	Scientific name	Common name	RefSeq Transcript accessions	RefSeq Protein accessions
101417560	*EGLN2*	*Dasypus novemcinctus*	Nine-banded armadillo	XM_004458965.3	XP_004459022.1
102248142	*EGLN2*	*Myotis brandtii*	Brandt's bat	XM_005868782.2	XP_005868844.1
102507989	*EGLN2*	*Camelus ferus*	Wild Bactrian camel	XM_032485997.1	XP_032341888.1
102541517	*EGLN2*	*Vicugna pacos*	Alpaca	XM_006214998.3	XP_006215060.1
110202901	*EGLN2*	*Phascolarctos cinereus*	Koala	XM_020979287.1	XP_020834946.1
112406	*Egln2*	*Mus musculus*	House mouse	NM_053208.4	NP_444438.2
536763	*EGLN2*	*Bos taurus*	Cattle	XM_024978408.1	XP_024834176.1
102409464	*EGLN2*	*Bubalus bubalis*	Water buffalo	XM_006052049.2	XP_006052111.1
484495	*EGLN2*	*Canis lupus familiaris*	Dog	XM_541609.6	XP_541609.1
100337938	*EGLN2*	*Oryctolagus cuniculus*	Rabbit	XM_002722315.3	XP_002722361.1
100397980	*EGLN2*	*Callithrix jacchus*	White-tufted-ear marmoset	XM_008988050.2	XP_008986298.1
100523673	*EGLN2*	*Sus scrofa*	Pig	XM_013988588.2	XP_013844042.2
100675805	*EGLN2*	*Loxodonta africana*	African savanna elephant	XM_010600351.2	XP_010598653.2
101145572	*EGLN2*	*Gorilla gorilla*	Western gorilla	XM_031004531.1	XP_030860391.1
101676101	*EGLN2*	*Mustela putorius furo*	Domestic ferret	XM_004776095.2	XP_004776152.1
102834615	*EGLN2*	*Chrysochloris asiatica*	Cape golden mole	XM_006871405.1	XP_006871467.1
103126975	*EGLN2*	*Erinaceus europaeus*	Western European hedgehog	XM_007537906.2	XP_007537968.1
103198763	*EGLN2*	*Orycteropus afer afer*	Aardvark	XM_007942970.1	XP_007941161.1
103661051	*EGLN2*	*Ursus maritimus*	Polar bear	XM_008688727.1	XP_008686949.1

Gene ID	Gene symbol	Scientific name	Common name	RefSeq Transcript accessions	RefSeq Protein accessions
105093358	*EGLN2*	*Camelus dromedarius*	Arabian camel	XM_031458744.1	XP_031314604.1
108397735	*EGLN2*	*Manis javanica*	Malayan pangolin	XM_017661282.1	XP_017516771.1
112851320	*EGLN2*	*Puma concolor*	Puma	XM_025914847.1	XP_025770632.1
113243552	*EGLN2*	*Ursus arctos horribilis*	Grizzly bear	XM_026482248.1	XP_026338033.1
114674212	*EGLN2*	*Macaca mulatta*	Rhesus monkey	XM_028840215.1	XP_028696048.1
118022639	*EGLN2*	*Mirounga leonina*	Southern elephant seal	XM_035023300.1	XP_034879191.1
116743605	*EGLN2*	*Phocoena sinus*	Vaquita	XM_032612242.1	XP_032468133.1
116857542	*EGLN2*	*Lontra canadensis*	Northern American river otter	XM_032841738.1	XP_032697629.1
103284295	*EGLN2*	*Eptesicus fuscus*	Big brown bat	XM_008139572.2	XP_008137794.1
101704717	*Egln2*	*Heterocephalus glaber*	Naked mole-rat	XM_004874230.3	XP_004874287.1
104855580	*Egln2*	*Fukomys damarensis*	Damara mole-rat	XM_010614452.3	XP_010612754.1
103750775	*Egln2*	*Nannospalax galili*	Upper Galilee mountains blind mole-rat	XM_008854018.3	XP_008852240.1
109697661	*EGLN2*	*Castor canadensis*	American beaver	XM_020181394.1	XP_020036983.1
		VHL			
101272871	*VHL*	*Orcinus orca*	Killer whale	XM_012534819.1	XP_012390273.1
101328054	*VHL*	*Tursiops truncatus*	Common bottlenose dolphin	XM_019944676.1	XP_019800235.1
102997043	*VHL*	*Physeter catodon*	Sperm whale	XM_007113722.3	XP_007113784.2
103007825	*VHL*	*Balaenoptera acutorostrata scammoni*	Minke whale	XM_028169104.1	XP_028024905.1
103080565	*VHL*	*Lipotes vexillifer*	Yangtze River dolphin	XM_007472386.1	XP_007472448.1

Gene ID	Gene symbol	Scientific name	Common name	RefSeq Transcript accessions	RefSeq Protein accessions
112412936	*VHL*	*Neophocaena asiaeorientalis asiaeorientalis*	Yangtze finless porpoise	XM_024764731. 1	XP_024620499. 1
113613236	*VHL*	*Lagenorhynchus obliquidens*	Pacific white-sided dolphin	XM_027093809. 1	XP_026949610. 1
114907217	*VHL*	*Monodon monoceros*	Narwhal	XM_029242627. 1	XP_029098460. 1
115844066	*VHL*	*Globicephala melas*	Long-finned pilot whale	XM_030840751. 1	XP_030696611. 1
101370163	*VHL*	*Odobenus rosmarus divergens*	Pacific walrus	XM_004392685. 1	XP_004392742. 1
102732787	*VHL*	*Leptonychotes weddellii*	Weddell seal	XM_006738990. 2	XP_006739053. 1
110584820	*VHL*	*Neomonachus schauinslandi*	Hawaiian monk seal	XM_021694988. 1	XP_021550663. 1
112833954	*VHL*	*Callorhinus ursinus*	Northern fur seal	XM_025885390. 1	XP_025741175. 1
113918488	*VHL*	*Zalophus californianus*	California sea lion	XM_027586962. 1	XP_027442763. 1
114207747	*VHL*	*Eumetopias jubatus*	Steller sea lion	XM_028101766. 1	XP_027957567. 1
116636786	*VHL*	*Phoca vitulina*	Harbor seal	XM_032411918. 1	XP_032267809. 1
111144039	*LOC111144039*	*Enhydra lutris kenyoni*	Sea otter	XM_022498110. 1	XP_022353818. 1
101352062	*LOC101352062*	*Trichechus manatus latirostris*	Florida manatee	XM_022498111. 1	XP_022353819. 1
7428	*VHL*	*Homo sapiens*	Human	NM_000551.4	NP_000542.1
100058139	*VHL*	*Equus caballus*	Horse	XM_023619976. 1	XP_023475744. 1
100739306	*VHL*	*Sus scrofa*	Pig	XM_003483214. 4	XP_003483262. 1
106996285	*VHL*	*Macaca mulatta*	Rhesus monkey	XM_028844440. 1	XP_028700273. 1
111750613	*VHL*	*Loxodonta africana*	African savanna elephant	XM_023548070. 1	XP_023403838. 1
102255400	*VHL*	*Myotis brandtii*	Brandt's bat	XM_014542692. 1	XP_014398178. 1

Gene ID	Gene symbol	Scientific name	Common name	RefSeq Transcript accessions	RefSeq Protein accessions
108408138	LOC108408138	*Manis javanica*	Malayan pangolin	XM_017677917.1	XP_017533406.1
101679898	*VHL*	*Mustela putorius furo*	Domestic ferret	XM_004738515.2	XP_004738572.1
22346	*Vhl*	*Mus musculus*	House mouse	NM_009507.4	NP_033533.1
494000	*VHL*	*Canis lupus familiaris*	Dog	NM_001008552.1	NP_001008552.1
540957	*VHL*	*Bos taurus*	Cattle	NM_001110019.1	NP_001103489.1
100350053	*VHL*	*Oryctolagus cuniculus*	Rabbit	XM_008249986.1	XP_008248208.1
101149351	*VHL*	*Gorilla gorilla*	Western gorilla	XM_004033589.3	XP_004033637.2
102182921	*VHL*	*Capra hircus*	Goat	XM_018038280.1	XP_017893769.1
102411667	*VHL*	*Bubalus bubalis*	Water buffalo	XM_006070695.2	XP_006070757.1
102543226	*VHL*	*Vicugna pacos*	Alpaca	XM_031686239.1	XP_031542099.1
102820834	*VHL*	*Chrysochloris asiatica*	Cape golden mole	XM_006872188.1	XP_006872250.1
103108250	*VHL*	*Erinaceus europaeus*	Western European hedgehog	XM_016185538.1	XP_016041024.1
103201218	*VHL*	*Orycteropus afer afer*	Aardvark	XM_007945859.1	XP_007944050.1
103788323	*VHL*	*Callithrix jacchus*	White-tufted-ear marmoset	XM_008982140.3	XP_008980388.1
105093118	*VHL*	*Camelus dromedarius*	Arabian camel	XM_031470872.1	XP_031326732.1
110204865	*VHL*	*Phascolarctos cinereus*	Koala	XM_020981009.1	XP_020836668.1
112858302	*VHL*	*Puma concolor*	Puma	XM_025921684.1	XP_025777469.1
113257162	*VHL*	*Ursus arctos horribilis*	Grizzly bear	XM_026501353.1	XP_026357138.1
114089813	*Vhl*	*Marmota flaviventris*	Yellow-bellied marmot	XM_027931755.1	XP_027787556.1

Gene ID	Gene symbol	Scientific name	Common name	RefSeq Transcript accessions	RefSeq Protein accessions
116657219	*VHL*	*Camelus ferus*	Wild Bactrian camel	XM_032458944.1	XP_032314835.1
116857445	*VHL*	*Lontra canadensis*	Northern American river otter	XM_032841506.1	XP_032697397.1
118005246	*VHL*	*Mirounga leonina*	Southern elephant seal	XM_034997081.1	XP_034852972.1
116762025	*VHL*	*Phocoena sinus*	Vaquita	XM_032648675.1	XP_032504566.1
109688948	*Vhl*	*Castor canadensis*	American beaver	XM_020167565.1	XP_020023154.1
			EGLN1		
101288091	*EGLN1*	*Orcinus orca*	Killer whale	XM_004281591.1	XP_004281639.1
101320491	*EGLN1*	*Tursiops truncatus*	Common bottlenose dolphin	XM_004310255.2	XP_004310303.2
102990364	*EGLN1*	*Physeter catodon*	Sperm whale	XM_007127611.3	XP_007127673.2
103012953	*EGLN1*	*Balaenoptera acutorostrata scammoni*	Minke whale	XM_028164414.1	XP_028020215.1
103086133	*EGLN1*	*Lipotes vexillifer*	Yangtze River dolphin	XM_007466989.1	XP_007467051.1
111163986	*EGLN1*	*Delphinapterus leucas*	Beluga whale	XM_022553237.2	XP_022408945.1
112413597	*EGLN1*	*Neophocaena asiaeorientalis asiaeorientalis*	Yangtze finless porpoise	XM_024765599.1	XP_024621367.1
113613377	*EGLN1*	*Lagenorhynchus obliquidens*	Pacific white-sided dolphin	XM_027094049.1	XP_026949850.1
114909037	*EGLN1*	*Monodon monoceros*	Narwhal	XM_029200849.1	XP_029056682.1
115843507	*EGLN1*	*Globicephala melas*	Long-finned pilot whale	XM_030839555.1	XP_030695415.1
101377787	*EGLN1*	*Odobenus rosmarus divergens*	Pacific walrus	XM_004404870.1	XP_004404927.1
102730055	*EGLN1*	*Leptonychotes weddellii*	Weddell seal	XM_006741755.2	XP_006741818.1
110586002	*EGLN1*	*Neomonachus schauinslandi*	Hawaiian monk seal	XM_021696149.1	XP_021551824.1

Gene ID	Gene symbol	Scientific name	Common name	RefSeq Transcript accessions	RefSeq Protein accessions
113919390	*EGLN1*	*Zalophus californianus*	California sea lion	XM_027588372.1	XP_027444173.1
114210271	*EGLN1*	*Eumetopias jubatus*	Steller sea lion	XM_028104518.1	XP_027960319.1
116648336	*EGLN1*	*Phoca vitulina*	Harbor seal	XM_032430269.1	XP_032286160.1
111160510	*LOC111160510*	*Enhydra lutris kenyoni*	Sea otter	XM_022523657.1	XP_022379365.1
101361248	*LOC101361248*	*Trichechus manatus latirostris*	Florida manatee	XM_004372812.3	XP_004372869.1
54583	*EGLN1*	*Homo sapiens*	Human	NM_022051.3	NP_071334.1
102505367	*EGLN1*	*Camelus ferus*	Wild Bactrian camel	XM_014558394.2	XP_014413880.2
103127458	*EGLN1*	*Erinaceus europaeus*	Western European hedgehog	XM_007538363.2	XP_007538425.2
113259033	*EGLN1*	*Ursus arctos horribilis*	Grizzly bear	XM_026504178.1	XP_026359963.1
112405	*Egln1*	*Mus musculus*	House mouse	NM_053207.2	NP_444437.2
488971	*EGLN1*	*Canis lupus familiaris*	Dog	XM_546089.5	XP_546089.3
534075	*EGLN1*	*Bos taurus*	Cattle	NM_001206046.2	NP_001192975.2
713410	*EGLN1*	*Macaca mulatta*	Rhesus monkey	XM_001104870.4	XP_001104870.1
100060551	*EGLN1*	*Equus caballus*	Horse	XM_014733482.2	XP_014588968.2
100153461	*EGLN1*	*Sus scrofa*	Pig	XM_021073580.1	XP_020929239.1
100357337	*EGLN1*	*Oryctolagus cuniculus*	Rabbit	XM_002717333.3	XP_002717379.1
100413526	*EGLN1*	*Callithrix jacchus*	White-tufted-ear marmoset	XM_003735291.4	XP_003735339.2
100672145	*EGLN1*	*Loxodonta africana*	African savanna elephant	XM_010591115.2	XP_010589417.2
101147171	*EGLN1*	*Gorilla gorilla*	Western gorilla	XM_004028581.3	XP_004028630.1

Gene ID	Gene symbol	Scientific name	Common name	RefSeq Transcript accessions	RefSeq Protein accessions
101684127	*EGLN1*	*Mustela putorius furo*	Domestic ferret	XM_004776033.1	XP_004776090.1
102189459	*EGLN1*	*Capra hircus*	Goat	XM_005698973.3	XP_005699030.3
102261609	*EGLN1*	*Myotis brandtii*	Brandt's bat	XM_005856381.2	XP_005856443.1
102400098	*EGLN1*	*Bubalus bubalis*	Water buffalo	XM_025284498.1	XP_025140283.1
102542335	*EGLN1*	*Vicugna pacos*	Alpaca	XM_015246203.2	XP_015101689.1
102821372	*EGLN1*	*Chrysochloris asiatica*	Cape golden mole	XM_006874166.1	XP_006874228.1
103195027	*EGLN1*	*Orycteropus afer afer*	Aardvark	XM_007938485.1	XP_007936676.1
105102153	*EGLN1*	*Camelus dromedarius*	Arabian camel	XM_031460867.1	XP_031316727.1
108394887	*EGLN1*	*Manis javanica*	Malayan pangolin	XM_017656729.1	XP_017512218.1
110217829	*EGLN1*	*Phascolarctos cinereus*	Koala	XM_021000417.1	XP_020856076.1
112851497	*EGLN1*	*Puma concolor*	Puma	XM_025915006.1	XP_025770791.1
114102517	*Egln1*	*Marmota flaviventris*	Yellow-bellied marmot	XM_027947985.1	XP_027803786.1
118012905	*EGLN1*	*Mirounga leonina*	Southern elephant seal	XM_035008458.1	XP_034864349.1
116857219	*EGLN1*	*Lontra canadensis*	Northern American river otter	XM_032841138.1	XP_032697029.1
103293430	*EGLN1*	*Eptesicus fuscus*	Big brown bat	XM_008149774.2	XP_008147996.2
116741497	*EGLN1*	*Phocoena sinus*	Vaquita	XM_032608131.1	XP_032464022.1
109694610	*Egln1*	*Castor canadensis*	American beaver	XM_020176652.1	XP_020032241.1
101709882	*Egln1*	*Heterocephalus glaber*	Naked mole-rat	XM_004854652.3	XP_004854709.1

Gene ID	Gene symbol	Scientific name	Common name	RefSeq Transcript accessions	RefSeq Protein accessions
104860710	*Egln1*	*Fukomys damarensis*	Damara mole-rat	XM_010621945.1	XP_010620247.1
103744145	*Egln1*	*Nannospalax galili*	Upper Galilee mountains blind mole-rat	XM_017801262.2	XP_017656751.1
118883033	*EGLN1*	*Balaenoptera musculus*	Blue whale	XM_036829570.1	XP_036685465.1
		HIF3A			
101281082	*HIF3A*	*Orcinus orca*	Killer whale	XM_004271142.1	XP_004271190.1
101323023	*HIF3A*	*Tursiops truncatus*	Common bottlenose dolphin	XM_019934990.1	XP_019790549.1
102987456	*HIF3A*	*Physeter catodon*	Sperm whale	XM_007102722.2	XP_007102784.1
103019266	*HIF3A*	*Balaenoptera acutorostrata scammoni*	Minke whale	XM_007168169.1	XP_007168231.1
103069286	*HIF3A*	*Lipotes vexillifer*	Yangtze River dolphin	XM_007458501.1	XP_007458563.1
111180713	*HIF3A*	*Delphinapterus leucas*	Beluga whale	XM_022585307.1	XP_022441015.1
112415619	*HIF3A*	*Neophocaena asiaeorientalis asiaeorientalis*	Yangtze finless porpoise	XM_024768729.1	XP_024624497.1
113606055	*HIF3A*	*Lagenorhynchus obliquidens*	Pacific white-sided dolphin	XM_027079925.1	XP_026935726.1
114888178	*HIF3A*	*Monodon monoceros*	Narwhal	XM_029210163.1	XP_029065996.1
115862265	*HIF3A*	*Globicephala melas*	Long-finned pilot whale	XM_030873839.1	XP_030729699.1
101372866	*HIF3A*	*Odobenus rosmarus divergens*	Pacific walrus	XM_004400951.2	XP_004401008.1
102732318	*HIF3A*	*Leptonychotes weddellii*	Weddell seal	XM_031040910.1	XP_030896770.1
110572790	*HIF3A*	*Neomonachus schauinslandi*	Hawaiian monk seal	XM_021681175.1	XP_021536850.1
112807076	*HIF3A*	*Callorhinus ursinus*	Northern fur seal	XM_025849723.1	XP_025705508.1
113936042	*HIF3A*	*Zalophus californianus*	California sea lion	XM_027618883.1	XP_027474684.1

Gene ID	Gene symbol	Scientific name	Common name	RefSeq Transcript accessions	RefSeq Protein accessions
114197103	*HIF3A*	*Eumetopias jubatus*	Steller sea lion	XM_028087978.1	XP_027943779.1
116622080	*HIF3A*	*Phoca vitulina*	Harbor seal	XM_032388253.1	XP_032244144.1
111160743	*HIF3A*	*Enhydra lutris kenyoni*	Sea otter	XM_022524071.1	XP_022379779.1
101359903	*LOC101359903*	*Trichechus manatus latirostris*	Florida manatee	XM_004381596.1	XP_004381653.1
64344	*HIF3A*	*Homo sapiens*	Human	NM_152795.4	NP_690008.2
100621574	*HIF3A*	*Sus scrofa*	Pig	XM_021094603.1	XP_020950262.1
717188	*HIF3A*	*Macaca mulatta*	Rhesus monkey	XM_001108464.4	XP_001108464.3
100071095	*HIF3A*	*Equus caballus*	Horse	XM_023649958.1	XP_023505726.1
102179524	*HIF3A*	*Capra hircus*	Goat	XM_018062641.1	XP_017918130.1
101425320	*HIF3A*	*Dasypus novemcinctus*	Nine-banded armadillo	XM_012524220.1	XP_012379674.1
102250475	*HIF3A*	*Myotis brandtii*	Brandt's bat	XM_005857848.2	XP_005857910.1
102511997	*HIF3A*	*Camelus ferus*	Wild Bactrian camel	XM_006191156.3	XP_006191218.2
102534517	*HIF3A*	*Vicugna pacos*	Alpaca	XM_006208535.3	XP_006208597.1
102820539	*HIF3A*	*Chrysochloris asiatica*	Cape golden mole	XM_006868442.1	XP_006868504.1
103121683	*HIF3A*	*Erinaceus europaeus*	Western European hedgehog	XM_007532212.2	XP_007532274.1
108398976	*HIF3A*	*Manis javanica*	Malayan pangolin	XM_017663430.1	XP_017518919.1
113243399	*HIF3A*	*Ursus arctos horribilis*	Grizzly bear	XM_026482004.1	XP_026337789.1
507699	*HIF3A*	*Bos taurus*	Cattle	NM_001105342.1	NP_001098812.1
101146641	*HIF3A*	*Gorilla gorilla*	Western gorilla	XM_004061014.3	XP_004061062.2

Gene ID	Gene symbol	Scientific name	Common name	RefSeq Transcript accessions	RefSeq Protein accessions
105104022	*HIF3A*	*Camelus dromedarius*	Arabian camel	XM_031458923.1	XP_031314783.1
476429	*HIF3A*	*Canis lupus familiaris*	Dog	XM_533636.6	XP_533636.3
100413902	*HIF3A*	*Callithrix jacchus*	White-tufted-ear marmoset	XM_035284829.1	XP_035140720.1
101676659	*HIF3A*	*Mustela putorius furo*	Domestic ferret	XM_004767532.2	XP_004767589.1
103657202	*HIF3A*	*Ursus maritimus*	Polar bear	XM_008684626.1	XP_008682848.1
110202372	*HIF3A*	*Phascolarctos cinereus*	Koala	XM_020978436.1	XP_020834095.1
114100950	*Hif3a*	*Marmota flaviventris*	Yellow-bellied marmot	XM_027945791.1	XP_027801592.1
53417	*Hif3a*	*Mus musculus*	House mouse	NM_001162950.1	NP_001156422.1
117998171	*HIF3A*	*Mirounga leonina*	Southern elephant seal	XM_034986813.1	XP_034842704.1
116855992	*HIF3A*	*Lontra canadensis*	Northern American river otter	XM_032839118.1	XP_032695009.1
103297805	*HIF3A*	*Eptesicus fuscus*	Big brown bat	XM_028129484.1	XP_027985285.1
116744669	*HIF3A*	*Phocoena sinus*	Vaquita	XM_032614715.1	XP_032470606.1
109691109	*Hif3a*	*Castor canadensis*	American beaver	XM_020170895.1	XP_020026484.1
101697834	*Hif3a*	*Heterocephalus glaber*	Naked mole-rat	XM_004867112.2	XP_004867169.1
104873912	*Hif3a*	*Fukomys damarensis*	Damara mole-rat	XM_010640856.2	XP_010639158.1
103724787	*Hif3a*	*Nannospalax galili*	Upper Galilee mountains blind mole-rat	XM_008821929.3	XP_008820151.1
118885190	*HIF3A*	*Balaenoptera musculus*	Blue whale	XM_036833639.1	XP_036689534.1
			HIF1AN		
101282716	*HIF1AN*	*Orcinus orca*	Killer whale	XM_004268458.2	XP_004268506.1

Gene ID	Gene symbol	Scientific name	Common name	RefSeq Transcript accessions	RefSeq Protein accessions
101326093	*HIF1AN*	*Tursiops truncatus*	Common bottlenose dolphin	XM_004310557.2	XP_004310605.2
102994002	*HIF1AN*	*Physeter catodon*	Sperm whale	XM_007101554.3	XP_007101616.2
103010817	*HIF1AN*	*Balaenoptera acutorostrata scammoni*	Minke whale	XM_007198676.2	XP_007198738.1
103087616	*HIF1AN*	*Lipotes vexillifer*	Yangtze River dolphin	XM_007470345.1	XP_007470407.1
111172388	*HIF1AN*	*Delphinapterus leucas*	Beluga whale	XM_022569048.1	XP_022424756.1
112392955	*HIF1AN*	*Neophocaena asiaeorientalis asiaeorientalis*	Yangtze finless porpoise	XM_024734863.1	XP_024590631.1
113623319	*HIF1AN*	*Lagenorhynchus obliquidens*	Pacific white-sided dolphin	XM_027112320.1	XP_026968121.1
114883458	*HIF1AN*	*Monodon monoceros*	Narwhal	XM_029201278.1	XP_029057111.1
115857902	*HIF1AN*	*Globicephala melas*	Long-finned pilot whale	XM_030865352.1	XP_030721212.1
101367069	*HIF1AN*	*Odobenus rosmarus divergens*	Pacific walrus	XM_004401921.2	XP_004401978.1
102738370	*HIF1AN*	*Leptonychotes weddellii*	Weddell seal	XM_006751050.2	XP_006751113.1
110589203	*HIF1AN*	*Neomonachus schauinslandi*	Hawaiian monk seal	XM_021699691.1	XP_021555366.1
112830766	*HIF1AN*	*Callorhinus ursinus*	Northern fur seal	XM_025880857.1	XP_025736642.1
113922416	*HIF1AN*	*Zalophus californianus*	California sea lion	XM_027594326.1	XP_027450127.1
114205221	*HIF1AN*	*Eumetopias jubatus*	Steller sea lion	XM_028098548.1	XP_027954349.1
116649608	*HIF1AN*	*Phoca vitulina*	Harbor seal	XM_032432593.1	XP_032288484.1
111145240	*HIF1AN*	*Enhydra lutris kenyoni*	Sea otter	XM_022500019.1	XP_022355727.1
101343053	*LOC101343053*	*Trichechus manatus latirostris*	Florida manatee	XM_004370036.3	XP_004370093.1
55662	*HIF1AN*	*Homo sapiens*	Human	NM_017902.3	NP_060372.2

Gene ID	Gene symbol	Scientific name	Common name	RefSeq Transcript accessions	RefSeq Protein accessions
319594	*Hif1an*	*Mus musculus*	House mouse	NM_176958.3	NP_795932.2
520864	*HIF1AN*	*Bos taurus*	Cattle	NM_001083443.2	NP_001076912.1
609221	*HIF1AN*	*Canis lupus familiaris*	Dog	XM_014108892.2	XP_013964367.1
710425	*HIF1AN*	*Macaca mulatta*	Rhesus monkey	XM_015147954.2	XP_015003440.1
100070337	*HIF1AN*	*Equus caballus*	Horse	XM_001500266.5	XP_001500316.1
100155854	*HIF1AN*	*Sus scrofa*	Pig	XM_003125588.4	XP_003125636.1
100347080	*HIF1AN*	*Oryctolagus cuniculus*	Rabbit	XM_002718653.3	XP_002718699.1
100415279	*HIF1AN*	*Callithrix jacchus*	White-tufted-ear marmoset	XM_009010157.3	XP_009008405.1
100665502	*HIF1AN*	*Loxodonta africana*	African savanna elephant	XM_003408975.3	XP_003409023.1
101140559	*HIF1AN*	*Gorilla gorilla*	Western gorilla	XM_004049950.3	XP_004049998.1
101426098	*HIF1AN*	*Dasypus novemcinctus*	Nine-banded armadillo	XM_004459179.3	XP_004459236.1
101670598	*HIF1AN*	*Mustela putorius furo*	Domestic ferret	XM_004749746.2	XP_004749803.1
102182662	*HIF1AN*	*Capra hircus*	Goat	XM_005698330.3	XP_005698387.1
102258997	*HIF1AN*	*Myotis brandtii*	Brandt's bat	XM_014532665.1	XP_014388151.1
102412343	*HIF1AN*	*Bubalus bubalis*	Water buffalo	XM_006053196.2	XP_006053258.1
102504253	*HIF1AN*	*Camelus ferus*	Wild Bactrian camel	XM_032491090.1	XP_032346981.1
102532041	*HIF1AN*	*Vicugna pacos*	Alpaca	XM_006210514.3	XP_006210576.1
102818291	*HIF1AN*	*Chrysochloris asiatica*	Cape golden mole	XM_006831248.1	XP_006831311.1
103119937	*HIF1AN*	*Erinaceus europaeus*	Western European hedgehog	XM_007530274.1	XP_007530336.1

Gene ID	Gene symbol	Scientific name	Common name	RefSeq Transcript accessions	RefSeq Protein accessions
103196256	*HIF1AN*	*Orycteropus afer afer*	Aardvark	XM_007939954.1	XP_007938145.1
103677605	*HIF1AN*	*Ursus maritimus*	Polar bear	XM_008706886.1	XP_008705108.1
105084325	*HIF1AN*	*Camelus dromedarius*	Arabian camel	XM_010974441.2	XP_010972743.1
108409589	*HIF1AN*	*Manis javanica*	Malayan pangolin	XM_017680255.1	XP_017535744.1
110194380	*HIF1AN*	*Phascolarctos cinereus*	Koala	XM_020966673.1	XP_020822332.1
112854606	*HIF1AN*	*Puma concolor*	Puma	XM_025918141.1	XP_025773926.1
113251834	*HIF1AN*	*Ursus arctos horribilis*	Grizzly bear	XM_026493891.1	XP_026349676.1
114088494	*Hif1an*	*Marmota flaviventris*	Yellow-bellied marmot	XM_027930145.1	XP_027785946.1
118025021	*HIF1AN*	*Mirounga leonina*	Southern elephant seal	XM_035027359.1	XP_034883250.1
116879831	*HIF1AN*	*Lontra canadensis*	Northern American river otter	XM_032878351.1	XP_032734242.1
103288480	*HIF1AN*	*Eptesicus fuscus*	Big brown bat	XM_008144189.2	XP_008142411.1
116741871	*HIF1AN*	*Phocoena sinus*	Vaquita	XM_032609033.1	XP_032464924.1
109685931	*Hif1an*	*Castor canadensis*	American beaver	XM_020163051.1	XP_020018640.1
101715790	*Hif1an*	*Heterocephalus glaber*	Naked mole-rat	XM_004866310.2	XP_004866367.1
104863760	*Hif1an*	*Fukomys damarensis*	Damara mole-rat	XM_010626366.3	XP_010624668.1
103743986	*Hif1an*	*Nannospalax galili*	Upper Galilee mountains blind mole-rat	XM_008845557.2	XP_008843779.1
118882527	*HIF1AN*	*Balaenoptera musculus*	Blue whale	XM_036828344.1	XP_036684239.1
ARNT					
101270436	*ARNT*	*Orcinus orca*	Killer whale	XM_004285163.2	XP_004285211.1

Gene ID	Gene symbol	Scientific name	Common name	RefSeq Transcript accessions	RefSeq Protein accessions
101336343	*ARNT*	*Tursiops truncatus*	Common bottlenose dolphin	XM_004318637.3	XP_019775169.1
102980846	*ARNT*	*Physeter catodon*	Sperm whale	XM_007130429.3	XP_007130491.1
103008733	*ARNT*	*Balaenoptera acutorostrata scammoni*	Minke whale	XM_007178005.2	XP_007178067.1
103075373	*ARNT*	*Lipotes vexillifer*	Yangtze River dolphin	XM_007460233.1	XP_007460295.1
111169763	*ARNT*	*Delphinapterus leucas*	Beluga whale	XM_022564215.2	XP_022419923.1
112411615	*ARNT*	*Neophocaena asiaeorientalis asiaeorientalis*	Yangtze finless porpoise	XM_024762834.1	XP_024618602.1
113615400	*ARNT*	*Lagenorhynchus obliquidens*	Pacific white-sided dolphin	XM_027097763.1	XP_026953564.1
114898784	*ARNT*	*Monodon monoceros*	Narwhal	XM_029228854.1	XP_029084687.1
115846986	*ARNT*	*Globicephala melas*	Long-finned pilot whale	XM_030846399.1	XP_030702259.1
101371497	*ARNT*	*Odobenus rosmarus divergens*	Pacific walrus	XM_012563694.1	XP_012419148.1
102731426	*ARNT*	*Leptonychotes weddellii*	Weddell seal	XM_006728336.2	XP_006728399.1
110578596	*ARNT*	*Neomonachus schauinslandi*	Hawaiian monk seal	XM_021688127.1	XP_021543802.1
114197246	*ARNT*	*Eumetopias jubatus*	Steller sea lion	XM_028088210.1	XP_027944011.1
112813248	*ARNT*	*Callorhinus ursinus*	Northern fur seal	XM_025858280.1	XP_025714065.1
113915092	*ARNT*	*Zalophus californianus*	California sea lion	XM_027581010.1	XP_027436811.1
116627371	*ARNT*	*Phoca vitulina*	Harbor seal	XM_032397141.1	XP_032253032.1
111161943	*LOC111161943*	*Enhydra lutris kenyoni*	Sea otter	XM_022526013.1	XP_022381721.1
101340519	*LOC101340519*	*Trichechus manatus latirostris*	Florida manatee	XM_023724606.1	XP_023580374.1
405	*ARNT*	*Homo sapiens*	Human	NM_001668.4	NP_001659.1

Gene ID	Gene symbol	Scientific name	Common name	RefSeq Transcript accessions	RefSeq Protein accessions
396653	*ARNT*	*Sus scrofa*	Pig	XM_013997187.2	XP_013852641.1
715971	*ARNT*	*Macaca mulatta*	Rhesus monkey	XM_015151767.2	XP_015007253.2
100059178	*ARNT*	*Equus caballus*	Horse	XM_023641234.1	XP_023497002.1
100673667	*ARNT*	*Loxodonta africana*	African savanna elephant	XM_003409485.3	XP_003409533.1
102183027	*ARNT*	*Capra hircus*	Goat	XM_018046044.1	XP_017901533.1
101428022	*ARNT*	*Dasypus novemcinctus*	Nine-banded armadillo	XM_023589718.1	XP_023445486.1
102258023	*ARNT*	*Myotis brandtii*	Brandt's bat	XM_014540670.1	XP_014396156.1
102511573	*ARNT*	*Camelus ferus*	Wild Bactrian camel	XM_032464342.1	XP_032320233.1
102545427	*ARNT*	*Vicugna pacos*	Alpaca	XM_006216953.3	XP_006217015.1
102835418	*ARNT*	*Chrysochloris asiatica*	Cape golden mole	XM_006861381.1	XP_006861443.1
103121049	*ARNT*	*Erinaceus europaeus*	Western European hedgehog	XM_007531542.2	XP_007531604.2
103205567	*ARNT*	*Orycteropus afer afer*	Aardvark	XM_007950924.1	XP_007949115.1
108408262	*ARNT*	*Manis javanica*	Malayan pangolin	XM_017678167.1	XP_017533656.1
110220577	*ARNT*	*Phascolarctos cinereus*	Koala	XM_021004640.1	XP_020860299.1
112872033	*ARNT*	*Puma concolor*	Puma	XM_025935165.1	XP_025790950.1
114106744	*Arnt*	*Marmota flaviventris*	Yellow-bellied marmot	XM_027953885.1	XP_027809686.1
103667537	*ARNT*	*Ursus maritimus*	Polar bear	XM_008695694.1	XP_008693916.1
113246207	*ARNT*	*Ursus arctos horribilis*	Grizzly bear	XM_026486719.1	XP_026342504.1
100008996	*ARNT*	*Oryctolagus cuniculus*	Rabbit	NM_001082206.1	NP_001075675.1

Gene ID	Gene symbol	Scientific name	Common name	RefSeq Transcript accessions	RefSeq Protein accessions
11863	*Arnt*	*Mus musculus*	House mouse	XM_006500930.4	XP_006500993.1
281010	*ARNT*	*Bos taurus*	Cattle	NM_173993.1	NP_776418.1
100410534	*ARNT*	*Callithrix jacchus*	White-tufted-ear marmoset	XM_002759890.3	XP_002759936.1
101144887	*ARNT*	*Gorilla gorilla*	Western gorilla	XM_004026601.3	XP_004026650.1
102407029	*ARNT*	*Bubalus bubalis*	Water buffalo	XM_006058124.2	XP_006058186.1
105103194	*ARNT*	*Camelus dromedarius*	Arabian camel	XM_010996740.2	XP_010995042.1
101677663	*ARNT*	*Mustela putorius furo*	Domestic ferret	XM_013047961.1	XP_012903415.1
116858720	*ARNT*	*Lontra canadensis*	Northern American river otter	XM_032843587.1	XP_032699478.1
103299034	*ARNT*	*Eptesicus fuscus*	Big brown bat	XM_028131649.1	XP_027987450.1
116750338	*ARNT*	*Phocoena sinus*	Vaquita	XM_032624992.1	XP_032480883.1
109681203	*Arnt*	*Castor canadensis*	American beaver	XM_020155811.1	XP_020011400.1
101717560	*Arnt*	*Heterocephalus glaber*	Naked mole-rat	XM_004854059.3	XP_004854116.1
104856876	*Arnt*	*Fukomys damarensis*	Damara mole-rat	XM_010616426.2	XP_010614728.1
103729101	*Arnt*	*Nannospalax galili*	Upper Galilee mountains blind mole-rat	XM_008827075.3	XP_008825297.2
118899159	*ARNT*	*Balaenoptera musculus*	Blue whale	XM_036860391.1	XP_036716286.1
118000312	*ARNT*	*Mirounga leonina*	Southern elephant seal	XM_034989956.1	XP_034845847.1
			ARNT2		
101284104	*ARNT2*	*Orcinus orca*	Killer whale	XM_004278326.1	XP_004278374.1
101319718	*ARNT2*	*Tursiops truncatus*	Common bottlenose dolphin	XM_033852233.1	XP_033708124.1

Gene ID	Gene symbol	Scientific name	Common name	RefSeq Transcript accessions	RefSeq Protein accessions
102985701	*ARNT2*	*Physeter catodon*	Sperm whale	XM_024129735.2	XP_023985503.2
103001180	*ARNT2*	*Balaenoptera acutorostrata scammoni*	Minke whale	XM_007194237.2	XP_007194299.1
103072248	*ARNT2*	*Lipotes vexillifer*	Yangtze River dolphin	XM_007447062.1	XP_007194300.1
111164743	*ARNT2*	*Delphinapterus leucas*	Beluga whale	XM_022554592.2	XP_022410300.1
112398307	*ARNT2*	*Neophocaena asiaeorientalis asiaeorientalis*	Yangtze finless porpoise	XM_024742786.1	XP_024598554.1
113610769	*ARNT2*	*Lagenorhynchus obliquidens*	Pacific white-sided dolphin	XM_027089166.1	XP_026944967.1
114903682	*ARNT2*	*Monodon monoceros*	Narwhal	XM_029236794.1	XP_029092627.1
115864592	*ARNT2*	*Globicephala melas*	Long-finned pilot whale	XM_030878463.1	XP_030734323.1
101382914	*ARNT2*	*Odobenus rosmarus divergens*	Pacific walrus	XM_004403573.1	XP_004403630.1
102727726	*ARNT2*	*Leptonychotes weddellii*	Weddell seal	XM_027089167.1	XP_026944968.1
110572400	*ARNT2*	*Neomonachus schauinslandi*	Hawaiian monk seal	XM_021680694.1	XP_021536369.1
112839957	*ARNT2*	*Callorhinus ursinus*	Northern fur seal	XM_025893128.1	XP_025748913.1
113909401	*ARNT2*	*Zalophus californianus*	California sea lion	XM_027570451.1	XP_027426252.1
114198119	*ARNT2*	*Eumetopias jubatus*	Steller sea lion	XM_028089417.1	XP_027945218.1
116624276	*ARNT2*	*Phoca vitulina*	Harbor seal	XM_032392058.1	XP_032247949.1
111152532	*LOC111152532*	*Enhydra lutris kenyoni*	Sea otter	XM_022511018.1	XP_022366726.1
101343137	*LOC101343137*	*Trichechus manatus latirostris*	Florida manatee	XM_004387497.1	XP_004387554.1
9915	*ARNT2*	*Homo sapiens*	Human	NM_014862.4	NP_055677.3

Gene ID	Gene symbol	Scientific name	Common name	RefSeq Transcript accessions	RefSeq Protein accessions
712786	*ARNT2*	*Macaca mulatta*	Rhesus monkey	XM_028851222.1	XP_028707055.1
608366	*ARNT2*	*Canis lupus familiaris*	Dog	XM_022417006.1	XP_022272714.1
101678509	*ARNT2*	*Mustela putorius furo*	Domestic ferret	XM_004763631.2	XP_004763688.1
100157219	*ARNT2*	*Sus scrofa*	Pig	XM_013989002.2	XP_013844456.1
100407922	*ARNT2*	*Callithrix jacchus*	White-tufted-ear marmoset	XM_008998512.3	XP_008996760.2
102544421	*ARNT2*	*Vicugna pacos*	Alpaca	XM_031691774.1	XP_031547634.1
110193839	*ARNT2*	*Phascolarctos cinereus*	Koala	XM_020965854.1	XP_020821513.1
11864	*Arnt2*	*Mus musculus*	House mouse	NM_007488.3	NP_031514.3
533445	*ARNT2*	*Bos taurus*	Cattle	NM_001206705.1	NP_001193634.1
712786	*ARNT2*	*Macaca mulatta*	Rhesus monkey	XM_028851222.1	XP_028707055.1
100067423	*ARNT2*	*Equus caballus*	Horse	XM_023649326.1	XP_023505094.1
100357873	*ARNT2*	*Oryctolagus cuniculus*	Rabbit	XM_008275295.2	XP_008273517.2
100677652	*ARNT2*	*Loxodonta africana*	African savanna elephant	XM_023552835.1	XP_023408603.1
101128323	*ARNT2*	*Gorilla gorilla*	Western gorilla	XM_004056650.3	XP_004056698.1
101432141	*ARNT2*	*Dasypus novemcinctus*	Nine-banded armadillo	XM_004480675.3	XP_004480732.1
102174701	*ARNT2*	*Capra hircus*	Goat	XM_018066308.1	XP_017921797.1
102398885	*ARNT2*	*Bubalus bubalis*	Water buffalo	XM_025271545.1	XP_025127330.1
102521434	*ARNT2*	*Camelus ferus*	Wild Bactrian camel	XM_032469156.1	XP_032325047.1
102820560	*ARNT2*	*Chrysochloris asiatica*	Cape golden mole	XM_006870911.1	XP_006870973.1

Gene ID	Gene symbol	Scientific name	Common name	RefSeq Transcript accessions	RefSeq Protein accessions
103116507	*ARNT2*	*Erinaceus europaeus*	Western European hedgehog	XM_007529935.2	XP_007529997.1
103200568	*ARNT2*	*Orycteropus afer afer*	Aardvark	XM_007945085.1	XP_007943276.1
103660293	*ARNT2*	*Ursus maritimus*	Polar bear	XM_008687882.1	XP_008686104.1
105094743	*ARNT2*	*Camelus dromedarius*	Arabian camel	XM_031440697.1	XP_031296557.1
108388523	*ARNT2*	*Manis javanica*	Malayan pangolin	XM_017647016.1	XP_017502505.1
113263574	*ARNT2*	*Ursus arctos horribilis*	Grizzly bear	XM_026510273.1	XP_026366058.1
114079355	*Arnt2*	*Marmota flaviventris*	Yellow-bellied marmot	XM_027920595.1	XP_027776396.1
116854696	*ARNT2*	*Lontra canadensis*	Northern American river otter	XM_032836798.1	XP_032692689.1
103304289	*ARNT2*	*Eptesicus fuscus*	Big brown bat	XM_028138549.1	XP_027994350.1
116748842	*ARNT2*	*Phocoena sinus*	Vaquita	XM_032622326.1	XP_032478217.1
109675119	*Arnt2*	*Castor canadensis*	American beaver	XM_020151912.1	XP_020007501.1
101715821	*Arnt2*	*Heterocephalus glaber*	Naked mole-rat	XM_013075071.1	XP_012930525.1
104860129	*Arnt2*	*Fukomys damarensis*	Damara mole-rat	XM_010621194.2_	XP_010619496.1
103750348	*Arnt2*	*Nannospalax galili*	Upper Galilee mountains blind mole-rat	XM_008853403.2	XP_008851625.1
118890805	*ARNT2*	*Balaenoptera musculus*	Blue whale	XM_036844102.1	XP_036699997.1
118023124	*ARNT2*	*Mirounga leonina*	Southern elephant seal	XM_035024268.1	XP_034880159.1
