# Supplementary material for: Adaptive strategies of aquatic mammals: Exploring the role of the HIF pathway and hypoxia tolerance
Source: Genet Mol Biol. 2024 Jan 19;46(3 Suppl 1):e20230140. doi: 10.1590/1678-4685-GMB-2023-0140 (PMC10802827; doi:10.1590/1678-4685-GMB-2023-0140)
Supplement: Table S3 - [file 1415-4757-GMB-46-03-s1-e20230140-s3.pdf]

Genetics and Molecular Biology
https://doi.org/10.1590/1678-4685-GMB-2023-0140

Supplementary Material to "Adaptive strategies of aquatic mammals: Exploring the role of the HIF pathway and hypoxia tolerance"

Table S3 - Coding sequences of phylogenetic closely related species used as references to mapping genomes without annotation.

Mapped Genome Species	Reference Genome Species	Gene Name	NCBI Reference Sequence
	Tursiops truncatus	*ARNT*	XM_004318637.3
	Tursiops truncatus	*ARNT2*	XM_033852233.1
	Tursiops truncatus	*EGLN1*	XM_004310255.2
	Tursiops truncatus	*EGLN2*	XM_033844313.1
	Tursiops truncatus	*EGLN3*	XM_019924248.1
Mesoplodon densirostris	*Tursiops truncatus*	*EPAS1*	XM_019942458.1
	Tursiops truncatus	*HIF1A*	XM_019923093.1
	Tursiops truncatus	*HIF1AN*	XM_004310557.2
	Tursiops truncatus	*HIF3A*	XM_019934990.1
	Tursiops truncatus	*VEGFA*	XM_033864164.1
	Tursiops truncatus	*VHL*	XM_019944676.1
	Tursiops truncatus	*ARNT*	XM_004318637.3
Mesoplodon europaeus	*Tursiops truncatus*	*ARNT2*	XM_033852233.1
	Tursiops truncatus	*EGLN1*	XM_004310255.2
	Tursiops truncatus	*EGLN2*	XM_033844313.1

**Mapped Genomes Species**	**Reference Genome Species**	**Gene Name**	**NCBI Reference Sequence**
	Tursiops truncatus	*EGLN3*	XM_019924248.1
	Tursiops truncatus	*EPAS1*	XM_019942458.1
	Tursiops truncatus	*HIF1A*	XM_019923093.1
	Tursiops truncatus	*HIF1AN*	XM_004310557.2
	Tursiops truncatus	*HIF3A*	XM_019934990.1
	Tursiops truncatus	*VEGFA*	XM_033864164.1
	Tursiops truncatus	*VHL*	XM_019944676.1
	Tursiops truncatus	*ARNT*	XM_004318637.3
	Tursiops truncatus	*ARNT2*	XM_033852233.1
	Tursiops truncatus	*EGLN1*	XM_004310255.2
	Tursiops truncatus	*EGLN2*	XM_033844313.1
	Tursiops truncatus	*EGLN3*	XM_019924248.1
Mesoplodon stejnegeri	*Tursiops truncatus*	*EPAS1*	XM_019942458.1
	Tursiops truncatus	*HIF1A*	XM_019923093.1
	Tursiops truncatus	*HIF1AN*	XM_004310557.2
	Tursiops truncatus	*HIF3A*	XM_019934990.1
	Tursiops truncatus	*VEGFA*	XM_033864164.1
	Tursiops truncatus	*VHL*	XM_019944676.1
	Tursiops truncatus	*ARNT*	XM_004318637.3
	Tursiops truncatus	*ARNT2*	XM_033852233.1
	Tursiops truncatus	*EGLN1*	XM_004310255.2
Cephalorhynchus commersonii	*Tursiops truncatus*	*EGLN2*	XM_033844313.1
	Tursiops truncatus	*EGLN3*	XM_019924248.1
	Tursiops truncatus	*EPAS1*	XM_019942458.1
	Tursiops truncatus	*HIF1A*	XM_019923093.1

Mapped Genomes Species	Reference Genome Species	Gene Name	NCBI Reference Sequence
	Tursiops truncatus	*HIF1AN*	XM_004310557.2
	Tursiops truncatus	*HIF3A*	XM_019934990.1
	Tursiops truncatus	*VEGFA*	XM_033864164.1
	Tursiops truncatus	*VHL*	XM_019944676.1
Peponocephala electra	*Tursiops truncatus*	*ARNT*	XM_004318637.3
	Tursiops truncatus	*ARNT2*	XM_033852233.1
	Tursiops truncatus	*EGLN1*	XM_004310255.2
	Tursiops truncatus	*EGLN2*	XM_033844313.1
	Tursiops truncatus	*EGLN3*	XM_019924248.1
	Tursiops truncatus	*EPAS1*	XM_019942458.1
	Tursiops truncatus	*HIF1A*	XM_019923093.1
	Tursiops truncatus	*HIF1AN*	XM_004310557.2
	Tursiops truncatus	*HIF3A*	XM_019934990.1
	Tursiops truncatus	*VEGFA*	XM_033864164.1
	Tursiops truncatus	*VHL*	XM_019944676.1
Steno bredanensis	*Tursiops truncatus*	*ARNT*	XM_004318637.3
	Tursiops truncatus	*ARNT2*	XM_033852233.1
	Tursiops truncatus	*EGLN1*	XM_004310255.2
	Tursiops truncatus	*EGLN2*	XM_033844313.1
	Tursiops truncatus	*EGLN3*	XM_019924248.1
	Tursiops truncatus	*EPAS1*	XM_019942458.1
	Tursiops truncatus	*HIF1A*	XM_019923093.1
	Tursiops truncatus	*HIF1AN*	XM_004310557.2
	Tursiops truncatus	*HIF3A*	XM_019934990.1
	Tursiops truncatus	*VEGFA*	XM_033864164.1

Mapped Genomes Species	Reference Genome Species	Gene Name	NCBI Reference Sequence
	Tursiops truncatus	*VHL*	XM_019944676.1
Grampus griseus	*Tursiops truncatus*	*ARNT*	XM_004318637.3
	Tursiops truncatus	*ARNT2*	XM_033852233.1
	Tursiops truncatus	*EGLN1*	XM_004310255.2
	Tursiops truncatus	*EGLN2*	XM_033844313.1
	Tursiops truncatus	*EGLN3*	XM_019924248.1
	Tursiops truncatus	*EPAS1*	XM_019942458.1
	Tursiops truncatus	*HIF1A*	XM_019923093.1
	Tursiops truncatus	*HIF1AN*	XM_004310557.2
	Tursiops truncatus	*HIF3A*	XM_019934990.1
	Tursiops truncatus	*VEGFA*	XM_033864164.1
	Tursiops truncatus	*VHL*	XM_019944676.1
Balaenoptera edeni	*Balaenoptera acutorostrata scammoni*	*ARNT*	XM_007178005.2
	Balaenoptera acutorostrata scammoni	*ARNT2*	XM_007194237.2
	Balaenoptera acutorostrata scammoni	*EGLN1*	XM_028164414.1
	Balaenoptera acutorostrata scammoni	*EGLN2*	XM_007198492.2
	Balaenoptera acutorostrata scammoni	*EGLN3*	XM_007180697.1
	Balaenoptera acutorostrata scammoni	*EPAS1*	XM_007190076.2
	Balaenoptera acutorostrata scammoni	*HIF1A*	XM_007192958.2
	Balaenoptera musculus	*HIF1AN*	XM_036828344.1
	Balaenoptera acutorostrata scammoni	*HIF3A*	XM_007168169.1
	Balaenoptera musculus	*VEGFA*	XM_036868005.1
	Balaenoptera acutorostrata scammoni	*VHL*	XM_028169104.1
Eubalaena glacialis	*Balaenoptera acutorostrata scammoni*	*ARNT*	XM_007178005.2
	Balaenoptera acutorostrata scammoni	*ARNT2*	XM_007194237.2

Mapped Genomes Species	Reference Genome Species	Gene Name	NCBI Reference Sequence
	Balaenoptera acutorostrata scammoni	*EGLN1*	XM_028164414.1
	Balaenoptera acutorostrata scammoni	*EGLN2*	XM_007198492.2
	Balaenoptera acutorostrata scammoni	*EGLN3*	XM_007180697.1
	Balaenoptera acutorostrata scammoni	*EPAS1*	XM_007190076.2
	Balaenoptera acutorostrata scammoni	*HIF1A*	XM_007192958.2
	Balaenoptera musculus	*HIF1AN*	XM_036828344.1
	Balaenoptera acutorostrata scammoni	*HIF3A*	XM_007168169.1
	Balaenoptera musculus	*VEGFA*	XM_036868005.1
	Balaenoptera acutorostrata scammoni	*VHL*	XM_028169104.1
	Mirounga leonina	*ARNT*	XM_034989956.1
	Mirounga leonina	*ARNT2*	XM_035024268.1
	Mirounga leonina	*EGLN1*	XM_035008458.1
	Mirounga leonina	*EGLN2*	XM_035023299.1
	Mirounga leonina	*EGLN3*	XM_006748618.2
Erignathus barbatus	*Mirounga leonina*	*EPAS1*	XM_035001638.1
	Mirounga leonina	*HIF1A*	XM_034992741.1
	Mirounga leonina	*HIF1AN*	XM_035027359.1
	Mirounga leonina	*HIF3A*	XM_034986813.1
	Mirounga leonina	*VEGFA*	XM_034991882.1
	Mirounga leonina	*VHL*	XM_034997081.1
	Enhydra lutris kenyoni	*ARNT*	XM_022526013.1
Aonyx cinereus	*Enhydra lutris kenyoni*	*ARNT2*	XM_022511018.1
	Enhydra lutris kenyoni	*EGLN1*	XM_022523657.1
	Enhydra lutris kenyoni	*EGLN2*	XM_022525065.1

Mapped Genomes Species	Reference Genome Species	Gene Name	NCBI Reference Sequence
	Enhydra lutris kenyoni	*EGLN3*	XM_022524334.1
	Enhydra lutris kenyoni	*EPAS1*	XM_022516312.1
	Enhydra lutris kenyoni	*HIF1A*	XM_022521812.1
	Enhydra lutris kenyoni	*HIF1AN*	XM_022500019.1
	Enhydra lutris kenyoni	*HIF3A*	XM_022524071.1
	Enhydra lutris kenyoni	*VEGFA*	XM_022503948.1
	Enhydra lutris kenyoni	*VHL*	XM_022498111.1
Balaenoptera bonaerensis	*Balaenoptera acutorostrata scammoni*	*ARNT*	XM_007178005.2
	Balaenoptera acutorostrata scammoni	*ARNT2*	XM_007194237.2
	Balaenoptera acutorostrata scammoni	*EGLN1*	XM_028164414.1
	Balaenoptera acutorostrata scammoni	*EGLN2*	XM_007198492.2
	Balaenoptera acutorostrata scammoni	*EGLN3*	XM_007180697.1
	Balaenoptera acutorostrata scammoni	*EPAS1*	XM_007190076.2
	Balaenoptera acutorostrata scammoni	*HIF1A*	XM_007192958.2
	Balaenoptera musculus	*HIF1AN*	XM_036828344.1
	Balaenoptera acutorostrata scammoni	*HIF3A*	XM_007168169.1
	Balaenoptera musculus	*VEGFA*	XM_036868005.1
	Balaenoptera acutorostrata scammoni	*VHL*	XM_028169104.1
Balaenoptera physalus	*Balaenoptera acutorostrata scammoni*	*ARNT*	XM_007178005.2
	Balaenoptera acutorostrata scammoni	*ARNT2*	XM_007194237.2
	Balaenoptera acutorostrata scammoni	*EGLN1*	XM_028164414.1
	Balaenoptera acutorostrata scammoni	*EGLN2*	XM_007198492.2
	Balaenoptera acutorostrata scammoni	*EGLN3*	XM_007180697.1
	Balaenoptera acutorostrata scammoni	*EPAS1*	XM_007190076.2

Mapped Genomes Species	Reference Genome Species	Gene Name	NCBI Reference Sequence
	Balaenoptera acutorostrata scammoni	*HIF1A*	XM_007192958.2
	Balaenoptera musculus	*HIF1AN*	XM_036828344.1
	Balaenoptera acutorostrata scammoni	*HIF3A*	XM_007168169.1
	Balaenoptera musculus	*VEGFA*	XM_036868005.1
	Balaenoptera acutorostrata scammoni	*VHL*	XM_028169104.1
Enhydra lutris nereis	*Enhydra lutris kenyoni*	*ARNT*	XM_022526013.1
	Enhydra lutris kenyoni	*ARNT2*	XM_022511018.1
	Enhydra lutris kenyoni	*EGLN1*	XM_022523657.1
	Enhydra lutris kenyoni	*EGLN2*	XM_022525065.1
	Enhydra lutris kenyoni	*EGLN3*	XM_022524334.1
	Enhydra lutris kenyoni	*EPAS1*	XM_022516312.1
	Enhydra lutris kenyoni	*HIF1A*	XM_022521812.1
	Enhydra lutris kenyoni	*HIF1AN*	XM_022500019.1
	Enhydra lutris kenyoni	*HIF3A*	XM_022524071.1
	Enhydra lutris kenyoni	*VEGFA*	XM_022503948.1
	Enhydra lutris kenyoni	*VHL*	XM_022498111.1
Eschrichtius robustus	*Balaenoptera acutorostrata scammoni*	*ARNT*	XM_007178005.2
	Balaenoptera acutorostrata scammoni	*ARNT2*	XM_007194237.2
	Balaenoptera acutorostrata scammoni	*EGLN1*	XM_028164414.1
	Balaenoptera acutorostrata scammoni	*EGLN2*	XM_007198492.2
	Balaenoptera acutorostrata scammoni	*EGLN3*	XM_007180697.1
	Balaenoptera acutorostrata scammoni	*EPAS1*	XM_007190076.2
	Balaenoptera acutorostrata scammoni	*HIF1A*	XM_007192958.2
	Balaenoptera musculus	*HIF1AN*	XM_036828344.1
	Balaenoptera acutorostrata scammoni	*HIF3A*	XM_007168169.1

Mapped Genomes Species	Reference Genome Species	Gene Name	NCBI Reference Sequence
	Balaenoptera musculus	*VEGFA*	XM_036868005.1
	Balaenoptera acutorostrata scammoni	*VHL*	XM_028169104.1
Eubalaena japonica	*Balaenoptera acutorostrata scammoni*	*ARNT*	XM_007178005.2
	Balaenoptera acutorostrata scammoni	*ARNT2*	XM_007194237.2
	Balaenoptera acutorostrata scammoni	*EGLN1*	XM_028164414.1
	Balaenoptera acutorostrata scammoni	*EGLN2*	XM_007198492.2
	Balaenoptera acutorostrata scammoni	*EGLN3*	XM_007180697.1
	Balaenoptera acutorostrata scammoni	*EPAS1*	XM_007190076.2
	Balaenoptera acutorostrata scammoni	*HIF1A*	XM_007192958.2
	Balaenoptera musculus	*HIF1AN*	XM_036828344.1
	Balaenoptera acutorostrata scammoni	*HIF3A*	XM_007168169.1
	Balaenoptera musculus	*VEGFA*	XM_036868005.1
	Balaenoptera acutorostrata scammoni	*VHL*	XM_028169104.1
Hippopotamus amphibius	*Tursiops truncatus*	*ARNT*	XM_004318637.3
	Tursiops truncatus	*ARNT2*	XM_033852233.1
	Tursiops truncatus	*EGLN1*	XM_004310255.2
	Tursiops truncatus	*EGLN2*	XM_033844313.1
	Tursiops truncatus	*EGLN3*	XM_019924248.1
	Tursiops truncatus	*EPAS1*	XM_019942458.1
	Tursiops truncatus	*HIF1A*	XM_019923093.1
	Tursiops truncatus	*HIF1AN*	XM_004310557.2
	Tursiops truncatus	*HIF3A*	XM_019934990.1
	Tursiops truncatus	*VEGFA*	XM_033864164.1
	Tursiops truncatus	*VHL*	XM_019944676.1
Inia geoffrensis	*Tursiops truncatus*	*ARNT*	XM_004318637.3

Mapped Genomes Species	Reference Genome Species	Gene Name	NCBI Reference Sequence
	Tursiops truncatus	*ARNT2*	XM_033852233.1
	Tursiops truncatus	*EGLN1*	XM_004310255.2
	Tursiops truncatus	*EGLN2*	XM_033844313.1
	Tursiops truncatus	*EGLN3*	XM_019924248.1
	Tursiops truncatus	*EPAS1*	XM_019942458.1
	Tursiops truncatus	*HIF1A*	XM_019923093.1
	Tursiops truncatus	*HIF1AN*	XM_004310557.2
	Tursiops truncatus	*HIF3A*	XM_019934990.1
	Tursiops truncatus	*VEGFA*	XM_033864164.1
	Tursiops truncatus	*VHL*	XM_019944676.1
Lutra lutra	*Enhydra lutris kenyoni*	*ARNT*	XM_022526013.1
	Enhydra lutris kenyoni	*ARNT2*	XM_022511018.1
	Enhydra lutris kenyoni	*EGLN1*	XM_022523657.1
	Enhydra lutris kenyoni	*EGLN2*	XM_022525065.1
	Enhydra lutris kenyoni	*EGLN3*	XM_022524334.1
	Enhydra lutris kenyoni	*EPAS1*	XM_022516312.1
	Enhydra lutris kenyoni	*HIF1A*	XM_022521812.1
	Enhydra lutris kenyoni	*HIF1AN*	XM_022500019.1
	Enhydra lutris kenyoni	*HIF3A*	XM_022524071.1
	Enhydra lutris kenyoni	*VEGFA*	XM_022503948.1
	Enhydra lutris kenyoni	*VHL*	XM_022498111.1
Megaptera novaeangliae	*Balaenoptera acutorostrata scammoni*	*ARNT*	XM_007178005.2
	Balaenoptera acutorostrata scammoni	*ARNT2*	XM_007194237.2
	Balaenoptera acutorostrata scammoni	*EGLN1*	XM_028164414.1
	Balaenoptera acutorostrata scammoni	*EGLN2*	XM_007198492.2

Mapped Genomes Species	Reference Genome Species	Gene Name	NCBI Reference Sequence
	Balaenoptera acutorostrata scammoni	*EGLN3*	XM_007180697.1
	Balaenoptera acutorostrata scammoni	*EPAS1*	XM_007190076.2
	Balaenoptera acutorostrata scammoni	*HIF1A*	XM_007192958.2
	Balaenoptera musculus	*HIF1AN*	XM_036828344.1
	Balaenoptera acutorostrata scammoni	*HIF3A*	XM_007168169.1
	Balaenoptera musculus	*VEGFA*	XM_036868005.1
	Balaenoptera acutorostrata scammoni	*VHL*	XM_028169104.1
	Mirounga leonina	*ARNT*	XM_034989956.1
	Mirounga leonina	*ARNT2*	XM_035024268.1
	Mirounga leonina	*EGLN1*	XM_035008458.1
	Mirounga leonina	*EGLN2*	XM_035023299.1
	Mirounga leonina	*EGLN3*	XM_006748618.2
Mirounga angustirostris	*Mirounga leonina*	*EPAS1*	XM_035001638.1
	Mirounga leonina	*HIF1A*	XM_034992741.1
	Mirounga leonina	*HIF1AN*	XM_035027359.1
	Mirounga leonina	*HIF3A*	XM_034986813.1
	Mirounga leonina	*VEGFA*	XM_034991882.1
	Mirounga leonina	*VHL*	XM_034997081.1
	Tursiops truncatus	*ARNT*	XM_004318637.3
	Tursiops truncatus	*ARNT2*	XM_033852233.1
	Tursiops truncatus	*EGLN1*	XM_004310255.2
Phocoena phocoena	*Tursiops truncatus*	*EGLN2*	XM_033844313.1
	Tursiops truncatus	*EGLN3*	XM_019924248.1
	Tursiops truncatus	*EPAS1*	XM_019942458.1
	Tursiops truncatus	*HIF1A*	XM_019923093.1

Mapped Genomes Species	Reference Genome Species	Gene Name	NCBI Reference Sequence
	Tursiops truncatus	*HIF1AN*	XM_004310557.2
	Tursiops truncatus	*HIF3A*	XM_019934990.1
	Tursiops truncatus	*VEGFA*	XM_033864164.1
	Tursiops truncatus	*VHL*	XM_019944676.1
Platanista minor	*Tursiops truncatus*	*ARNT*	XM_004318637.3
	Tursiops truncatus	*ARNT2*	XM_033852233.1
	Tursiops truncatus	*EGLN1*	XM_004310255.2
	Tursiops truncatus	*EGLN2*	XM_033844313.1
	Tursiops truncatus	*EGLN3*	XM_019924248.1
	Tursiops truncatus	*EPAS1*	XM_019942458.1
	Tursiops truncatus	*HIF1A*	XM_019923093.1
	Tursiops truncatus	*HIF1AN*	XM_004310557.2
	Tursiops truncatus	*HIF3A*	XM_019934990.1
	Tursiops truncatus	*VEGFA*	XM_033864164.1
	Tursiops truncatus	*VHL*	XM_019944676.1
Pontoporia blainvillei	*Tursiops truncatus*	*ARNT*	XM_004318637.3
	Tursiops truncatus	*ARNT2*	XM_033852233.1
	Tursiops truncatus	*EGLN1*	XM_004310255.2
	Tursiops truncatus	*EGLN2*	XM_033844313.1
	Tursiops truncatus	*EGLN3*	XM_019924248.1
	Tursiops truncatus	*EPAS1*	XM_019942458.1
	Tursiops truncatus	*HIF1A*	XM_019923093.1
	Tursiops truncatus	*HIF1AN*	XM_004310557.2
	Tursiops truncatus	*HIF3A*	XM_019934990.1
	Tursiops truncatus	*VEGFA*	XM_033864164.1

Mapped Genomes Species	Reference Genome Species	Gene Name	NCBI Reference Sequence
	Tursiops truncatus	*VHL*	XM_019944676.1
Pteronura brasiliensis	*Enhydra lutris kenyoni*	*ARNT*	XM_022526013.1
	Enhydra lutris kenyoni	*ARNT2*	XM_022511018.1
	Enhydra lutris kenyoni	*EGLN1*	XM_022523657.1
	Enhydra lutris kenyoni	*EGLN2*	XM_022525065.1
	Enhydra lutris kenyoni	*EGLN3*	XM_022524334.1
	Enhydra lutris kenyoni	*EPAS1*	XM_022516312.1
	Enhydra lutris kenyoni	*HIF1A*	XM_022521812.1
	Enhydra lutris kenyoni	*HIF1AN*	XM_022500019.1
	Enhydra lutris kenyoni	*HIF3A*	XM_022524071.1
	Enhydra lutris kenyoni	*VEGFA*	XM_022503948.1
	Enhydra lutris kenyoni	*VHL*	XM_022498111.1
Sousa chinensis	*Tursiops truncatus*	*ARNT*	XM_004318637.3
	Tursiops truncatus	*ARNT2*	XM_033852233.1
	Tursiops truncatus	*EGLN1*	XM_004310255.2
	Tursiops truncatus	*EGLN2*	XM_033844313.1
	Tursiops truncatus	*EGLN3*	XM_019924248.1
	Tursiops truncatus	*EPAS1*	XM_019942458.1
	Tursiops truncatus	*HIF1A*	XM_019923093.1
	Tursiops truncatus	*HIF1AN*	XM_004310557.2
	Tursiops truncatus	*HIF3A*	XM_019934990.1
	Tursiops truncatus	*VEGFA*	XM_033864164.1
	Tursiops truncatus	*VHL*	XM_019944676.1
Tursiops aduncus	*Tursiops truncatus*	*ARNT*	XM_004318637.3
	Tursiops truncatus	*ARNT2*	XM_033852233.1

**Mapped Genomes Species**	**Reference Genome Species**	**Gene Name**	**NCBI Reference Sequence**
	Tursiops truncatus	*EGLN1*	XM_004310255.2
	Tursiops truncatus	*EGLN2*	XM_033844313.1
	Tursiops truncatus	*EGLN3*	XM_019924248.1
	Tursiops truncatus	*EPAS1*	XM_019942458.1
	Tursiops truncatus	*HIF1A*	XM_019923093.1
	Tursiops truncatus	*HIF1AN*	XM_004310557.2
	Tursiops truncatus	*HIF3A*	XM_019934990.1
	Tursiops truncatus	*VEGFA*	XM_033864164.1
	Tursiops truncatus	*VHL*	XM_019944676.1
Ziphius cavirostris	*Tursiops truncatus*	*ARNT*	XM_004318637.3
	Tursiops truncatus	*ARNT2*	XM_033852233.1
	Tursiops truncatus	*EGLN1*	XM_004310255.2
	Tursiops truncatus	*EGLN2*	XM_033844313.1
	Tursiops truncatus	*EGLN3*	XM_019924248.1
	Tursiops truncatus	*EPAS1*	XM_019942458.1
	Tursiops truncatus	*HIF1A*	XM_019923093.1
	Tursiops truncatus	*HIF1AN*	XM_004310557.2
	Tursiops truncatus	*HIF3A*	XM_019934990.1
	Tursiops truncatus	*VEGFA*	XM_033864164.1
	Tursiops truncatus	*VHL*	XM_019944676.1
