# Supplementary material for: Adaptive strategies of aquatic mammals: Exploring the role of the HIF pathway and hypoxia tolerance
Source: Genet Mol Biol. 2024 Jan 19;46(3 Suppl 1):e20230140. doi: 10.1590/1678-4685-GMB-2023-0140 (PMC10802827; doi:10.1590/1678-4685-GMB-2023-0140)
Supplement: Table S4 - [file 1415-4757-GMB-46-03-s1-e20230140-s4.pdf]

Genetics and Molecular Biology
https://doi.org/10.1590/1678-4685-GMB-2023-0140

Supplementary Material to "Adaptive strategies of aquatic mammals: Exploring the role of the HIF pathway and hypoxia tolerance"

Table S4 - Aquatic mammals classification according to diving ability.

Scientific name	Order	Suborder	Family	Common duration (min)	Max. duration (min)	Common deep (m)	Max. deep (m)	Diving ability	References
Balaenoptera acutorostrata	Cetacea	Misticeti	Balenopteridae	1.6	~ 3.33			Shallow-divers	Stockin et al., 2001
*Balaenoptera musculus**	Cetacea	Misticeti	Balenopteridae	4.2-7.2	15.9	16-32.6	41-310	Shallow-divers	Hamilton et al., 2018; Bird et al., 2020
*Balaenoptera physalus**	Cetacea	Misticeti	Balenopteridae	3-5	20	180-200	474	Shallow-divers	Ponganis, 2011; Perrin and Wursig, 2009; Croll et al., 2001
Balaenoptera bonaerensis	Cetacea	Misticeti	Balenopteridae	3.2	7.2-12.2	18	57-105	Shallow-divers	Ishii et al., 2017; Friedlaender et al., 2014
*Balaenoptera edeni**	Cetacea	Misticeti	Balenopteridae	3-5	17	-	300	Shallow-divers	Kato et al., 2018; Steiner et al., 2008; Gonçalves et al., 2015
Megaptera novaeangliae	Cetacea	Misticeti	Balenopteridae	1-4	3	23-118	160	Shallow-divers	Ponganis, 2011; Perrin and Wursig, 2009
Eubalaena glacialis	Cetacea	Misticeti	Balaenidae	10-20	-	0-200	-	Shallow-divers	Jacobsen et al., 2004; Baumgartner et al., 2003; Kenney, 2009; Gregr and Coyle, 2009

Scientific name	Order	Suborder	Family	Common duration (min)	Max. duration (min)	Common deep (m)	Max deep (m)	Diving ability	References
Eubalaena japonica	Cetacea	Misticeti	Balaenidae	10-20	-	0-200	-	Shallow-divers	Kenney,2009; Gregr and Coyle, 2009
Eschrichtius robustus	Cetacea	Misticeti	Eschrichtiidae	2.24	28	4-10	27.4	Shallow-divers	Stelle et al., 2008; Urbán et al., 2003; Perrin and Wursig, 2009
Physeter catodon	Cetacea	Odontoceti	Physeteridae	40-60	138	400-900	2250	Deep-divers	Ponganis, 2011
Kogia breviceps	Cetacea	Odontoceti	Physeteridae	11	>45	400–1000	3500	Deep-divers	Bloodworth and Odell, 2008
Mesoplodon densirostris	Cetacea	Odontoceti	Ziphiidae	47-55	84	835-1099	1599	Deep-divers	Ponganis, 2011
Mesoplodon europaeus	Cetacea	Odontoceti	Ziphiidae	-	88	870	870 +/- 151	Deep-divers	Gillespie et al., 2009; DeAngelis et al., 2017
*Mesoplodon stejnegeri**	Cetacea	Odontoceti	Ziphiidae	-	-	-	-	Undefined	-
Mesoplodon bidens	Cetacea	Odontoceti	Ziphiidae	12-28	-	-	-	Deep-divers	Hooker and Baird, 1999
Ziphius cavirostris	Cetacea	Odontoceti	Ziphiidae	58-70	137.5	1070-1334	2992	Deep-divers	Ponganis, 2011; Schorr et al., 2014
Peponocephala electra	Cetacea	Odontoceti	Delphinidae	-	-	0-100	400	Deep-divers	Perryman et al., 2018
Grampus griseus	Cetacea	Odontoceti	Delphinidae	2-4	9-10	50	400–500	Deep-divers	Wells et al., 2009
Globicephala melas	Cetacea	Odontoceti	Delphinidae	5-15	21	100-800	1019	Deep-divers	Ponganis, 2011
Monodon monoceros	Cetacea	Odontoceti	Monodontidae	<5	20	8-52	>1000	Deep-divers	Perrin and Wursig, 2009
Delphinapterus leucas	Cetacea	Odontoceti	Monodontidae	13	23	400-700	872	Deep-divers	Perrin and Wursig, 2009
*Orcinus orca**	Cetacea	Odontoceti	Delphinidae	1-10	15.9	12-96	767.5	Shallow-divers	Heyning et al., 1988. Perrin and Wursig, 2009. Ropert-Coudert et al., 2018
Tursiops truncatus	Cetacea	Odontoceti	Delphinidae	1	8	20	390	Shallow-divers	Ponganis, 2011
Tursiops aduncus	Cetacea	Odontoceti	Delphinidae	-	-	14		Shallow-divers	Stensland et al., 2006

Scientific name	Order	Suborder	Family	Common duration (min)	Max. duration (min)	Common deep (m)	Max deep (m)	Diving ability	References
*Lagenorhynchus obliquidens**	Cetacea	Odontoceti	Delphinidae	0.4	6.2	0–200	>200	Shallow-divers	Black, 1994
*Steno bredanensis**	Cetacea	Odontoceti	Delphinidae	-	15	-	70	Shallow-divers	West et al., 2011
Sousa chinensis	Cetacea	Odontoceti	Delphinidae	-	-	11	25	Shallow-divers	Wang et al., 2007
*Cephalorhynchus commersonii**	Cetacea	Odontoceti	Delphinidae	2	7	-	-	Shallow-divers	Coscarella et al., 2010
Lipotes vexillifer	Cetacea	Odontoceti	Lipotidae	0.33	2.25	-	-	Shallow-divers	Ropert-Coudert et al., 2018; Renjun et al., 1994
Phocoena sinus	Cetacea	Odontoceti	Phocoenidae	1.38	2.45	-	-	Shallow-divers	Silber et al., 1988
*Neophocaena asiaeorientalis**	Cetacea	Odontoceti	Phocoenidae	0.29	1.08	-	-	Shallow-divers	Zhou et al., 1980
Phocoena phocoena	Cetacea	Odontoceti	Phocoenidae	1	5	14-40	226	Shallow-divers	Ponganis, 2011
Inia geoffrensis	Cetacea	Odontoceti	Iniidae	1-1.6	2.5			Shallow-divers	Renjun et al., 1994
Pontoporia blainvillei	Cetacea	Odontoceti	Pontoporiidae	-	-	0-30	30	Shallow-divers	Crespo and De Cidre, 2005
*Platanista minor**	Cetacea	Odontoceti	Platanistidae	0.73	3	2.4-5.2	-	Shallow-divers	Ropert-Coudert et al., 2018; Akbar et al., 2004
Callorhinus ursinus	Carnivora	Pinnipedia/Caniformia	Otariidae	2	8	65	256	Shallow-divers	Ponganis, 2011
Zalophus californianus	Carnivora	Pinnipedia/Caniformia	Otariidae	2	10	62	274	Shallow-divers	Ponganis, 2011
Eumetopias jubatus	Carnivora	Pinnipedia/Caniformia	Otariidae	<2	8	9-24	250	Shallow-divers	Ponganis, 2011
Odobenus rosmarus	Carnivora	Pinnipedia/Caniformia	Odobenidae	0.5	15.2	10-35	80	Shallow-divers	Noren et al., 2015. Jay et al., 2006
Neomonachus schauinslandi	Carnivora	Pinnipedia/Caniformia	Phocidae	3–6	25	4.4–32	80-300	Deep-divers	Wilson et al., 2017. Norris et al., 2017
Leptonychotes weddellii	Carnivora	Pinnipedia/Caniformia	Phocidae	13-24	27.5	163.21	455-904	Deep-divers	Noren et al., 2015; Jay et al., 2006

Scientific name	Order	Suborder	Family	Common duration (min)	Max. duration (min)	Common deep (m)	Max deep (m)	Diving ability	References
Phoca vitulina	Carnivora	Pinnipedia/Caniformia	Phocidae	1-2	35	5-100	446	Deep-divers	Ponganis, 2011; Perrin and Wursig, 2009
Mirounga leonina	Carnivora	Pinnipedia/Caniformia	Phocidae	20-29	120	269-552	1256	Deep-divers	Ponganis, 2011
Mirounga angustirostris	Carnivora	Pinnipedia/Caniformia	Phocidae	23	119	437	1581	Deep-divers	Ponganis, 2011
Erignathus barbatus	Carnivora	Pinnipedia/Caniformia	Phocidae	5.6–7.7	24	20–29	472	Deep-divers	Hamilton et al., 2018. Bird et al., 2020
*Enhydra lutris kenyoni**	Carnivora	Caniformia	Lutrinae/Musteloidea	~1	2.43	2-30	49	Shallow-divers	Bodkin et al., 2004
*Enhydra lutris nereis**	Carnivora	Caniformia	Lutrinae/Musteloidea	~1	2.43	2-30	49	Shallow-divers	Bodkin et al., 2004
*Lontra canadensis**	Carnivora	Caniformia	Lutrinae/Musteloidea	0.5-0.66	0.83-1	3	14	Shallow-divers	Reed-Smith et al., 2012
*Aonyx cinereus**	Carnivora	Caniformia	Lutrinae/Musteloidea	-	-	-	-	Undefined	-
*Lutra lutra**	Carnivora	Caniformia	Lutrinae/Musteloidea	0.3-0.1	1	0-3	8	Shallow-divers	Hung et al., 2016; Kruuk et al., 1990; Pfeier et al., 1998
*Pteronura brasiliensis**	Carnivora	Caniformia	Lutrinae/Musteloidea	0.2	1.2	<3	-	Shallow-divers	Duplaix, 1980
*Trichechus manatus latirostris**	Sirenia	-	Trichechidae	0.67 - 2.41	15.16	1	16	Shallow-divers	Edwards et al., 2016; Morales et al., 2012; Ropert-Coudert et al., 2018

Shallow-divers: ~100m in shallow waters. Deep-divers: ≥300m of depth, ~40 minutes. Species that were excluded from the nested branch analysis: *
