# Supplementary material for: Adaptive strategies of aquatic mammals: Exploring the role of the HIF pathway and hypoxia tolerance
Source: Genet Mol Biol. 2024 Jan 19;46(3 Suppl 1):e20230140. doi: 10.1590/1678-4685-GMB-2023-0140 (PMC10802827; doi:10.1590/1678-4685-GMB-2023-0140)
Supplement: Table S5 - [file 1415-4757-GMB-46-03-s1-e20230140-s5.pdf]

Genetics and Molecular Biology
https://doi.org/10.1590/1678-4685-GMB-2023-0140

Supplementary Material to "Adaptive strategies of aquatic mammals: Exploring the role of the HIF pathway and hypoxia tolerance"

Table S5 - Presence of relaxed selection and constraint on aquatic mammals.

Gene	log L		LRT	p-value	K
	Null model (all k=1)	Alternative model			
		All aquatic mammals			
ARNT	-16924.8	-16915.6	18.44	0.000	0.44
ARNT2	-18255.0	-18252.5	5	0.025	0.86
EGLN2	-11126.6	-11123.9	5.42	0.020	0.58
HIF1AN	-6980.7	-6973.4	14.53	0.000	0.6
		Cetaceans			
ARNT	-16915.4	-16909.6		0.001	0.66
ARNT2	-18241.2	-18238.5	5.34	0.021	0.77
EGLN1	-8435.0	-8426.7	16.42	0.000	0
EGLN2	-11126.3	-11120.5	11.54	0.001	0.39
HIF1AN	-6978.5	-6970.9	15.24	0.000	0.56
EPAS1	-28432.5	-28427.7	9.71	0.002	0.76
VEGFA	-3644.7	-3642.5	4.33	0.037	30.99
		Pinnipeda			
HIF1AN	-6972.2	-6974.3	4.19	0.041	0.46
		Lutrinae			
EGLN3	-4812.8	-4810.0	5.56	0.018	43.09
