# Supplementary material for: Adaptive strategies of aquatic mammals: Exploring the role of the HIF pathway and hypoxia tolerance
Source: Genet Mol Biol. 2024 Jan 19;46(3 Suppl 1):e20230140. doi: 10.1590/1678-4685-GMB-2023-0140 (PMC10802827; doi:10.1590/1678-4685-GMB-2023-0140)
Supplement: Table S6 - [file 1415-4757-GMB-46-03-s1-e20230140-s6.pdf]

*Genetics and Molecular Biology*
https://doi.org/10.1590/1678-4685-GMB-2023-0140

# Supplementary Material to “Adaptive strategies of aquatic mammals: Exploring the role of the HIF pathway and hypoxia tolerance”

**Table S6 -** Parameter estimates of BUSTED method for HIF pathway genes.

| Gene | Model | log L | p-value (LRT) | ω1 | ω2 | ω3 |
|---|---|---|---|---|---|---|
| *ARNT* | Unconstrained | -16741.2 | 0.005 | 0.06 (56.70%) | 0.13 (41.75%) | 8.22 (1.55%) |
| | Constrained | -16745.9 | | 0.01 (40.68%) | 0.01 (42.03%) | 1.00 (17.29%) |
| *ARNT2* | Unconstrained | -17916.0 | 0.010 | 0.04 (97.38%) | 0.06 (2.62%) | 1701.29 (0.01%) |
| | Constrained | -17919.9 | | 0.02 (97.32%) | 1.00 (2.08%) | 1.00 (0.60%) |
| *EPAS1* | Unconstrained | -27848.9 | 0.000 | 0.16 (99.83%) | 1.00 (0.16%) | 456.22 (0.01%) |
| | Constrained | -27858.5 | | 0.04 (86.68%) | 1.00 (12.51%) | 1.00 (0.82%) |
