# Supplementary material for: Adaptive strategies of aquatic mammals: Exploring the role of the HIF pathway and hypoxia tolerance
Source: Genet Mol Biol. 2024 Jan 19;46(3 Suppl 1):e20230140. doi: 10.1590/1678-4685-GMB-2023-0140 (PMC10802827; doi:10.1590/1678-4685-GMB-2023-0140)
Supplement: Table S7 - [file 1415-4757-GMB-46-03-s1-e20230140-s7.pdf]

Genetics and Molecular Biology
https://doi.org/10.1590/1678-4685-GMB-2023-0140

Supplementary Material to "Adaptive strategies of aquatic mammals: Exploring the role of the HIF pathway and hypoxia tolerance"

Table S7 - Selective pressure analyses of HIF pathway genes by PAML site model method.

Gene	Likelihood model	lnL	p-value	Parameter estimate	Positively selected sites (BEB PP > 0.90)
ARNT	M7	-16747.64602	0.003	*p0 = 0.22538, q = 1.40267*	271
	M8	-16741.80322		*p0 = 0.99364 (p1 = 0.00636), p = 0.24829, q = 1.71056, ω = 1.75965*	
EPAS1	M7	-27959.49006		*p0 = 0.26941, q = 1.62358*	664, 732, 762
	M8	-27946.66864	< 0.001	*p0 = 0.96783 (p1 = 0.03217), p = 0.30514, q = 2.36078, ω = 1.00011*	
HIF1A	M7	-16184.30675		*p0 = 0.16971, q = 1.21721*	593, 599, 612, 613, 615, 617, 658
	M8	-16168.63128	< 0.001	*p0 = 0.94807 (p1 = 0.05193), p = 0.28265, q = 3.76257, ω = 1.00000*	
HIF1AN	M7	-6872.116259		*p0 = 0.07459, q = 0.81896*	23
	M8	-6868.847185	0.038	*p0 = 0.97057 (p1 = 0.02943), p = 0.09674, q = 1.82198, ω = 1.00487*	
VHL	M1	-10091.25679		*p0 = 0.85060 (p1 = 0.14940)*	23, 68, 125, 195, 212
	M2	-10049.58266	< 0.001	*p0 = 0.84158, p1 = 0.13574 (p2 = 0.02268), ω2 = 3.16109*	
	M7	-10004.75568		*p0 = 0.14665, q = 0.63434*	23, 68, 125, 195, 212
	M8	-9958.401152	< 0.001	*p0 = 0.97699 (p1 = 0.02301), p = 0.18513, q = 1.20751, ω = 2.53410*	
HIF3A	M1	-18249.32976		*p0 = 0.80159 (p1 = 0.19841)*	364, 437

Gene	Likelihood model	lnL	p-value	Parameter estimate	Positively selected sites (BEB PP > 0.90)
	M2	-18241.65187	< 0.001	*p0 = 0.80055, p1 = 0.19526 (p2 = 0.00419), ω2 = 3.95739*	
	M7	-18126.17059		*p0 = 0.20578, q = 0.89272*	364, 427, 437
	M8	-18113.67842	< 0.001	*p0 = 0.99222 (p1 = 0.00778), p = 0.21934, q = 1.04415, ω = 2.80021*	
