# Supplementary material for: Adaptive strategies of aquatic mammals: Exploring the role of the HIF pathway and hypoxia tolerance
Source: Genet Mol Biol. 2024 Jan 19;46(3 Suppl 1):e20230140. doi: 10.1590/1678-4685-GMB-2023-0140 (PMC10802827; doi:10.1590/1678-4685-GMB-2023-0140)
Supplement: Table S8 - [file 1415-4757-GMB-46-03-s1-e20230140-s8.pdf]

Genetics and Molecular Biology
https://doi.org/10.1590/1678-4685-GMB-2023-0140

Supplementary Material to "Adaptive strategies of aquatic mammals: Exploring the role of the HIF pathway and hypoxia tolerance"

Table S8 - Positively selected sites from FUBAR and MEME.

Gene	FUBAR		MEME	
	**Site**	**Posterior probability**	**Site**	**p-value**
HIF3A	590	0.987	23	0.04
	626	0.936	60	0.00
			63	0.07
			74	0.02
			140	0.08
			145	0.00
			148	0.07
			182	0.09
			230	0.07
			327	0.09
			356	0.05
			368	0.07
			371	0.07
			373	0.00
			419	0.08
			497	0.08
			575	0.09
			590	0.00
			598	0.03
			612	0.08
			619	0.08
			634	0.10
			652	0.00
			661	0.08

Gene	FUBAR		MEME	
	Site	Posterior probability	Site	p-value
VHL	8	0.956	8	0.04
	28	0.984	23	0.00
			28	0.08
			53	0.08
			79	0.07
			108	0.00
			195	0.02
ARNT	60	0.992	2	0.00
	271	0.989	3	0.00
	704	0.925	6	0.01
			7	0.01
			8	0.01
			20	0.01
			57	0.05
			58	0.04
			60	0.00
			200	0.09
			271	0.02
			280	0.03
			329	0.08
			340	0.02
			341	0.05
			342	0.01
			423	0.06
			523	0.03
			572	0.05
			575	0.04
			594	0.07
			636	0.07
			704	0.06
HIF1A	596	0.928	4	0.09
			5	0.03

Gene	FUBAR		MEME	
	**Site**	**Posterior probability**	**Site**	**p-value**
			7	0.02
			236	0.03
			237	0.06
			367	0.04
			596	0.04
			624	0.00
			650	0.09
			687	0.05
			698	0.03
			781	0.08
			787	0.01
EPAS1	265	0.900	2	0.07
	496	0.942	3	0.02
			4	0.06
			5	0.03
			8	0.00
			11	0.06
			45	0.10
			51	0.00
			55	0.02
			71	0.07
			72	0.00
			223	0.09
			262	0.06
			265	0.05
			304	0.03
			479	0.08
			483	0.00
			484	0.00
			496	0.09
			577	0.01
			638	0.04

Gene	FUBAR		MEME	
	**Site**	**Posterior probability**	**Site**	**p-value**
			698	0.08
			721	0.10
			765	0.00
			766	0.06
			777	0.01
			798	0.05
			799	0.04
EGLN2	9	0.928	9	0.03
	53	0.916	21	0.07
	149	0.912	53	0.01
			66	0.10
			149	0.09
			302	0.03
VEGFA	31	0.905	31	0.09
	34	0.961	110	0.05
	143	0.923	140	0.09
			143	0.03
EGLN1			14	0.00
			15	0.03
			25	0.02
			29	0.01
			30	0.00
			31	0.02
			42	0.09
			47	0.00
			72	0.05
			87	0.03
			98	0.08
			137	0.05
			145	0.08
			147	0.01
			149	0.03

Gene	FUBAR		MEME	
	Site	Posterior probability	Site	p-value
			155	0.03
			167	0.01
			169	0.07
			230	0.08
			232	0.01
EGLN3			4	0.02
			60	0.07
ARNT2			18	0.03
			32	0.01
			38	0.04
			243	0.04
			265	0.00
			477	0.00
			673	0.01
			676	0.03
