# Supplementary material for: Adaptive strategies of aquatic mammals: Exploring the role of the HIF pathway and hypoxia tolerance
Source: Genet Mol Biol. 2024 Jan 19;46(3 Suppl 1):e20230140. doi: 10.1590/1678-4685-GMB-2023-0140 (PMC10802827; doi:10.1590/1678-4685-GMB-2023-0140)
Supplement: Table S9 - [file 1415-4757-GMB-46-03-s1-e20230140-s9.pdf]

Genetics and Molecular Biology
https://doi.org/10.1590/1678-4685-GMB-2023-0140

Supplementary Material to "Adaptive strategies of aquatic mammals: Exploring the role of the HIF pathway and hypoxia tolerance"

Table S9 - FUBAR evidence of site purifying selection.

Site	Prob[$\alpha > \beta$]	Site	Prob[$\alpha > \beta$]	Site	Prob[$\alpha > \beta$]	Site	Prob[$\alpha > \beta$]	Site	Prob[$\alpha > \beta$]
ARNT		*ARNT2*		*EGLN1*		*HIF1AN*		*HIF1A*	
434	0.902	13	0.901	158	1.000	10	0.900	602	0.900
655	0.902	244	0.913	177	1.000	334	0.903	737	0.900
480	0.904	572	0.917	188	1.000	281	0.906	704	0.902
406	0.905	518	0.920	202	1.000	218	0.911	353	0.903
668	0.909	56	0.931	206	1.000	254	0.913	69	0.904
684	0.909	65	0.932	260	1.000	227	0.914	422	0.907
341	0.910	2	0.933	286	1.000	246	0.914	625	0.907
729	0.911	477	0.933	291	1.000	269	0.914	3	0.909
701	0.912	539	0.934	292	1.000	27	0.915	566	0.909
774	0.912	330	0.936	294	1.000	76	0.915	408	0.910
15	0.915	503	0.936	296	1.000	167	0.915	64	0.913
512	0.922	637	0.940	298	1.000	169	0.915	771	0.913
408	0.923	195	0.946	299	1.000	179	0.915	287	0.918
194	0.924	394	0.947	313	1.000	200	0.915	484	0.921
597	0.926	171	0.948	358	1.000	277	0.915	719	0.921
22	0.927	699	0.948	365	1.000	278	0.915	330	0.922

Site	Prob[$\alpha > \beta$]	Site	Prob[$\alpha > \beta$]	Site	Prob[$\alpha > \beta$]	Site	Prob[$\alpha > \beta$]	Site	Prob[$\alpha > \beta$]
451	0.927	628	0.952	368	1.000	296	0.915	183	0.924
600	0.930	104	0.953	385	1.000	260	0.917	454	0.925
687	0.930	434	0.953	406	1.000	326	0.917	823	0.925
707	0.930	436	0.953	116	0.999	12	0.918	88	0.926
789	0.930	226	0.954	152	0.999	188	0.918	117	0.926
122	0.931	320	0.954	165	0.999	266	0.918	206	0.927
661	0.931	107	0.955	173	0.999	268	0.918	349	0.927
603	0.932	621	0.955	196	0.999	150	0.919	438	0.927
176	0.934	91	0.956	207	0.999	155	0.920	528	0.927
121	0.935	26	0.958	220	0.999	317	0.921	694	0.927
215	0.935	141	0.958	226	0.999	170	0.923	713	0.927
640	0.935	60	0.959	240	0.999	49	0.925	558	0.929
727	0.935	18	0.961	250	0.999	243	0.925	43	0.930
730	0.935	37	0.962	273	0.999	290	0.925	573	0.930
172	0.936	144	0.962	301	0.999	245	0.926	711	0.930
317	0.936	596	0.962	302	0.999	1	0.927	1	0.932
323	0.936	1	0.964	359	0.999	161	0.927	150	0.932
459	0.937	279	0.964	390	0.999	168	0.927	189	0.932
493	0.937	541	0.964	404	0.999	341	0.927	316	0.932
558	0.937	683	0.964	28	0.998	349	0.927	749	0.932
586	0.937	163	0.965	33	0.998	327	0.928	317	0.933
204	0.938	192	0.965	57	0.998	309	0.930	116	0.935
315	0.938	350	0.965	252	0.998	315	0.930	791	0.935
436	0.938	574	0.965	283	0.998	311	0.931	204	0.936
438	0.938	20	0.966	300	0.998	72	0.932	366	0.936
613	0.938	178	0.966	308	0.998	118	0.932	498	0.936
130	0.940	216	0.966	412	0.998	303	0.932	590	0.937
132	0.940	289	0.966	175	0.997	250	0.933	236	0.939
301	0.940	410	0.966	255	0.997	41	0.934	407	0.939
77	0.941	657	0.966	257	0.997	130	0.934	801	0.940
242	0.941	660	0.966	281	0.997	108	0.938	166	0.942

Site	Prob[$\alpha > \beta$]	Site	Prob[$\alpha > \beta$]	Site	Prob[$\alpha > \beta$]	Site	Prob[$\alpha > \beta$]	Site	Prob[$\alpha > \beta$]
425	0.941	550	0.967	297	0.997	114	0.938	208	0.942
542	0.941	472	0.968	329	0.997	165	0.938	571	0.943
604	0.941	547	0.968	364	0.997	191	0.938	742	0.943
611	0.941	389	0.969	182	0.996	275	0.938	220	0.944
24	0.942	293	0.970	235	0.996	319	0.938	448	0.944
110	0.942	645	0.971	338	0.996	325	0.938	616	0.944
251	0.942	30	0.972	362	0.996	343	0.938	531	0.945
424	0.942	64	0.972	374	0.996	248	0.941	721	0.945
582	0.942	252	0.972	334	0.995	20	0.943	237	0.946
699	0.942	43	0.974	335	0.995	23	0.943	391	0.947
713	0.942	583	0.974	233	0.994	117	0.943	706	0.947
768	0.942	105	0.975	274	0.994	90	0.948	240	0.948
31	0.943	128	0.975	56	0.993	240	0.948	328	0.948
183	0.943	238	0.976	83	0.993	255	0.948	532	0.948
338	0.943	701	0.976	89	0.993	96	0.952	766	0.948
460	0.943	207	0.977	310	0.993	226	0.953	392	0.949
720	0.943	242	0.977	400	0.993	258	0.953	447	0.949
144	0.944	529	0.977	179	0.992	216	0.954	10	0.950
196	0.944	4	0.978	271	0.992	314	0.954	512	0.950
221	0.944	558	0.978	399	0.992	136	0.955	689	0.951
294	0.944	629	0.978	183	0.991	174	0.956	276	0.952
528	0.944	15	0.979	216	0.991	147	0.957	156	0.953
584	0.944	50	0.979	254	0.991	137	0.961	759	0.953
84	0.945	8	0.980	384	0.991	53	0.968	277	0.955
345	0.945	79	0.980	295	0.989	159	0.968	98	0.957
374	0.945	89	0.980	199	0.987	323	0.968	231	0.957
498	0.945	452	0.980	239	0.987	59	0.969	365	0.957
516	0.945	589	0.980	241	0.987	105	0.969	783	0.957
379	0.946	681	0.980	242	0.987	58	0.970	591	0.958
588	0.946	687	0.980	268	0.987	298	0.970	658	0.959
614	0.946	690	0.980	304	0.987	16	0.971	202	0.961

Site	Prob[$\alpha > \beta$]	Site	Prob[$\alpha > \beta$]	Site	Prob[$\alpha > \beta$]	Site	Prob[$\alpha > \beta$]	Site	Prob[$\alpha > \beta$]
83	0.948	708	0.980	357	0.987	74	0.974	13	0.962
224	0.948	711	0.980	46	0.986	82	0.974	123	0.963
389	0.948	12	0.981	343	0.986	182	0.974	147	0.963
421	0.948	35	0.981	411	0.986	289	0.974	424	0.963
447	0.948	88	0.981	157	0.985	345	0.974	665	0.963
632	0.948	108	0.981	224	0.984	21	0.975	707	0.963
635	0.948	113	0.981	401	0.984	40	0.975	739	0.963
18	0.949	116	0.981	305	0.983	104	0.975	804	0.963
218	0.949	172	0.981	318	0.983	133	0.975	60	0.964
742	0.949	206	0.981	306	0.982	194	0.975	84	0.964
761	0.949	223	0.981	336	0.982	297	0.975	101	0.964
213	0.950	227	0.981	345	0.982	310	0.975	109	0.964
664	0.950	229	0.981	366	0.982	84	0.976	120	0.964
520	0.952	231	0.981	395	0.982	86	0.976	171	0.964
231	0.953	241	0.981	23	0.981	329	0.976	182	0.964
336	0.953	259	0.981	387	0.981	18	0.977	217	0.964
464	0.953	294	0.981	342	0.980	95	0.977	250	0.964
633	0.954	326	0.981	289	0.979	186	0.977	263	0.964
422	0.956	328	0.981	197	0.977	97	0.978	294	0.964
690	0.956	331	0.981	350	0.977	306	0.978	307	0.964
131	0.957	400	0.981	112	0.976	158	0.979	369	0.964
312	0.957	498	0.981	258	0.976	66	0.980	435	0.964
440	0.957	594	0.981	288	0.973	126	0.980	469	0.964
565	0.957	648	0.981	18	0.972	148	0.980	491	0.964
1	0.958	77	0.982	180	0.972	177	0.980	527	0.964
409	0.958	96	0.982	408	0.972	233	0.980	561	0.964
457	0.958	199	0.982	93	0.971	267	0.980	568	0.964
657	0.958	342	0.982	215	0.971	272	0.980	15	0.965
554	0.959	68	0.983	348	0.971	276	0.980	54	0.965
272	0.960	250	0.983	24	0.970	175	0.981	298	0.965
641	0.960	460	0.983	269	0.970	30	0.982	357	0.965

Site	Prob[$\alpha > \beta$]	Site	Prob[$\alpha > \beta$]	Site	Prob[$\alpha > \beta$]	Site	Prob[$\alpha > \beta$]	Site	Prob[$\alpha > \beta$]
453	0.961	193	0.984	2	0.969	131	0.982	405	0.965
703	0.961	321	0.984	53	0.969	197	0.982	592	0.965
98	0.962	85	0.985	154	0.969	61	0.983	765	0.965
367	0.962	93	0.985	201	0.969	144	0.983	40	0.966
396	0.962	99	0.985	204	0.969	211	0.983	72	0.966
398	0.962	132	0.985	208	0.968	94	0.984	86	0.966
403	0.962	239	0.985	361	0.968	163	0.984	104	0.966
450	0.962	337	0.985	405	0.968	280	0.985	163	0.966
629	0.962	354	0.985	209	0.967	320	0.985	296	0.966
97	0.963	406	0.985	52	0.966	238	0.986	321	0.966
220	0.963	433	0.985	328	0.966	279	0.987	453	0.966
125	0.964	70	0.986	19	0.964	109	0.988	502	0.966
170	0.964	129	0.986	153	0.963	111	0.988	574	0.966
525	0.964	287	0.986	218	0.963	19	0.989	747	0.966
48	0.965	368	0.986	356	0.962	215	0.989	22	0.967
50	0.965	417	0.986	129	0.961	195	0.990	228	0.967
79	0.965	427	0.986	21	0.960	204	0.990	364	0.967
87	0.965	482	0.986	323	0.959	316	0.990	587	0.967
216	0.965	631	0.986	131	0.958	129	0.991	693	0.967
290	0.965	673	0.986	44	0.957	132	0.991	734	0.967
324	0.965	712	0.986	48	0.956	146	0.991	818	0.967
368	0.965	78	0.987	62	0.956	223	0.991	682	0.968
419	0.965	373	0.987	317	0.954	274	0.991	775	0.968
448	0.965	418	0.987	234	0.953	299	0.991	789	0.968
452	0.965	548	0.987	170	0.952	45	0.992	259	0.969
461	0.965	684	0.987	280	0.952	99	0.992	278	0.969
553	0.965	69	0.988	360	0.951	287	0.992	315	0.969
667	0.965	670	0.988	16	0.950	331	0.992	431	0.969
775	0.965	25	0.989	108	0.950	337	0.992	436	0.969
109	0.966	248	0.989	396	0.950	83	0.993	790	0.969
544	0.966	371	0.989	386	0.947	68	0.994	103	0.970

Site	Prob[$\alpha > \beta$]	Site	Prob[$\alpha > \beta$]	Site	Prob[$\alpha > \beta$]	Site	Prob[$\alpha > \beta$]	Site	Prob[$\alpha > \beta$]
708	0.966	485	0.989	278	0.945	71	0.994	301	0.970
373	0.967	656	0.989	376	0.943	127	0.994	567	0.970
484	0.967	188	0.990	285	0.942	157	0.994	151	0.972
599	0.967	334	0.990	332	0.942	212	0.994	19	0.973
607	0.967	405	0.990	397	0.941	282	0.994	169	0.973
716	0.967	11	0.991	394	0.940	302	0.994	248	0.973
128	0.968	308	0.991	178	0.939	304	0.994	377	0.973
291	0.968	552	0.991	267	0.939	330	0.994	507	0.973
427	0.968	587	0.991	68	0.938	43	0.995	576	0.973
585	0.968	397	0.992	275	0.937	85	0.995	715	0.973
660	0.968	585	0.992	346	0.935	93	0.995	744	0.973
763	0.968	653	0.992	367	0.935	101	0.995	143	0.974
766	0.968	312	0.993	130	0.934	102	0.995	223	0.974
19	0.969	7	0.994	84	0.933	120	0.995	695	0.974
32	0.969	292	0.994	198	0.930	173	0.995	700	0.974
331	0.969	319	0.994	373	0.929	176	0.995	701	0.974
342	0.969	618	0.994	82	0.926	184	0.995	71	0.975
605	0.969	505	0.995	259	0.926	237	0.995	118	0.975
746	0.969	674	0.995	353	0.925	251	0.995	310	0.975
752	0.969	83	0.996	402	0.925	261	0.995	368	0.975
105	0.970	210	0.996	287	0.924	285	0.995	370	0.975
134	0.970	245	0.996	162	0.923	338	0.995	35	0.976
139	0.970	246	0.996	190	0.922	17	0.996	146	0.976
181	0.970	255	0.996	261	0.922	88	0.996	159	0.976
253	0.970	285	0.996	264	0.922	152	0.996	269	0.976
255	0.970	302	0.996	311	0.920	166	0.996	323	0.976
257	0.970	461	0.996	314	0.920	196	0.996	339	0.976
267	0.970	512	0.996	326	0.920	220	0.996	358	0.976
354	0.970	522	0.996	32	0.918	247	0.996	471	0.976
384	0.970	635	0.996	71	0.918	262	0.996	473	0.976
423	0.970	49	0.997	339	0.918	321	0.996	534	0.976

Site	Prob[$\alpha > \beta$]	Site	Prob[$\alpha > \beta$]	Site	Prob[$\alpha > \beta$]	Site	Prob[$\alpha > \beta$]	Site	Prob[$\alpha > \beta$]
426	0.970	54	0.997	85	0.912	98	0.997	595	0.976
666	0.970	58	0.997	1	0.910	128	0.997	729	0.976
205	0.971	81	0.997	8	0.910	187	0.997	318	0.977
479	0.971	82	0.997	17	0.910	199	0.997	432	0.977
347	0.973	101	0.997	333	0.910	206	0.997	475	0.977
522	0.973	165	0.997	309	0.908	210	0.997	526	0.977
95	0.974	198	0.997	6	0.907	217	0.997	712	0.977
513	0.974	211	0.997	127	0.907	224	0.997	792	0.977
275	0.975	327	0.997	331	0.907	342	0.997	813	0.977
276	0.975	333	0.997	344	0.907	3	0.998	149	0.978
728	0.976	369	0.997	27	0.906	62	0.998	720	0.978
113	0.977	379	0.997	322	0.905	64	0.998	24	0.979
476	0.979	392	0.997	355	0.905	154	0.998	48	0.979
37	0.980	445	0.997	377	0.905	180	0.998	286	0.979
259	0.980	499	0.997	246	0.903	232	0.998	648	0.980
197	0.981	516	0.997	211	0.902	346	0.998	14	0.981
737	0.981	636	0.997	221	0.902	4	0.999	108	0.981
126	0.982	652	0.997	293	0.902	29	0.999	167	0.981
314	0.982	696	0.997	282	0.901	42	0.999	337	0.981
443	0.982	705	0.997	*EGLN2*		46	0.999	464	0.981
468	0.982	41	0.998	187	0.904	54	0.999	681	0.981
556	0.982	55	0.998	65	0.905	55	0.999	91	0.982
580	0.982	62	0.998	365	0.905	57	0.999	252	0.982
624	0.982	97	0.998	97	0.912	67	0.999	406	0.982
123	0.983	112	0.998	69	0.913	69	0.999	520	0.982
141	0.983	118	0.998	370	0.913	77	0.999	533	0.982
159	0.983	156	0.998	265	0.915	80	0.999	559	0.982
164	0.983	177	0.998	256	0.916	87	0.999	605	0.982
361	0.983	181	0.998	173	0.917	103	0.999	714	0.982
431	0.983	194	0.998	45	0.923	107	0.999	50	0.983
462	0.983	230	0.998	184	0.924	122	0.999	244	0.983

Site	Prob[$\alpha > \beta$]	Site	Prob[$\alpha > \beta$]	Site	Prob[$\alpha > \beta$]	Site	Prob[$\alpha > \beta$]	Site	Prob[$\alpha > \beta$]
33	0.984	268	0.998	190	0.926	125	0.999	246	0.983
34	0.984	282	0.998	211	0.928	143	0.999	282	0.983
117	0.984	299	0.998	14	0.930	181	0.999	304	0.983
118	0.984	310	0.998	371	0.930	185	0.999	493	0.983
149	0.984	317	0.998	90	0.934	214	0.999	500	0.983
360	0.984	325	0.998	235	0.935	219	0.999	509	0.983
780	0.984	336	0.998	178	0.937	221	0.999	579	0.983
23	0.985	375	0.998	140	0.938	225	0.999	676	0.983
146	0.985	376	0.998	306	0.938	230	0.999	809	0.983
152	0.985	377	0.998	37	0.940	234	0.999	811	0.983
174	0.985	384	0.998	273	0.940	249	0.999	155	0.984
225	0.985	429	0.998	51	0.941	5	1.000	523	0.984
273	0.985	439	0.998	63	0.941	6	1.000	275	0.985
334	0.985	466	0.998	160	0.941	11	1.000	334	0.985
348	0.985	495	0.998	367	0.941	31	1.000	736	0.985
393	0.985	526	0.998	134	0.942	32	1.000	67	0.986
407	0.985	532	0.998	236	0.942	33	1.000	193	0.986
618	0.985	536	0.998	312	0.942	35	1.000	239	0.986
638	0.985	581	0.998	345	0.942	37	1.000	494	0.986
759	0.985	603	0.998	77	0.944	39	1.000	572	0.986
244	0.986	622	0.998	295	0.945	50	1.000	599	0.986
420	0.986	649	0.998	133	0.949	52	1.000	505	0.987
478	0.986	650	0.998	135	0.950	56	1.000	16	0.988
137	0.987	669	0.998	144	0.951	60	1.000	185	0.988
212	0.987	695	0.998	389	0.951	63	1.000	786	0.988
280	0.987	700	0.998	60	0.952	65	1.000	61	0.989
350	0.987	706	0.998	126	0.952	70	1.000	142	0.989
376	0.987	3	0.999	216	0.953	73	1.000	439	0.989
483	0.987	34	0.999	17	0.954	75	1.000	483	0.989
518	0.987	47	0.999	61	0.955	78	1.000	537	0.989
672	0.987	61	0.999	193	0.955	81	1.000	547	0.989

Site	Prob[$\alpha > \beta$]	Site	Prob[$\alpha > \beta$]	Site	Prob[$\alpha > \beta$]	Site	Prob[$\alpha > \beta$]	Site	Prob[$\alpha > \beta$]
770	0.987	86	0.999	263	0.957	89	1.000	560	0.989
46	0.988	142	0.999	293	0.957	91	1.000	564	0.989
201	0.988	149	0.999	64	0.958	100	1.000	679	0.989
503	0.988	150	0.999	99	0.958	106	1.000	764	0.989
567	0.988	173	0.999	360	0.958	110	1.000	2	0.990
642	0.988	179	0.999	343	0.959	115	1.000	77	0.990
644	0.988	187	0.999	376	0.960	116	1.000	164	0.990
711	0.988	240	0.999	32	0.961	121	1.000	555	0.990
692	0.989	247	0.999	180	0.961	124	1.000	190	0.991
733	0.989	249	0.999	251	0.961	135	1.000	274	0.991
150	0.990	264	0.999	272	0.961	139	1.000	441	0.991
364	0.990	265	0.999	277	0.961	140	1.000	478	0.991
527	0.990	272	0.999	385	0.961	149	1.000	510	0.991
9	0.991	273	0.999	22	0.962	153	1.000	780	0.991
96	0.991	274	0.999	73	0.962	156	1.000	56	0.992
111	0.991	286	0.999	74	0.965	172	1.000	59	0.992
188	0.991	303	0.999	201	0.965	183	1.000	95	0.992
203	0.991	305	0.999	398	0.965	189	1.000	300	0.992
247	0.991	307	0.999	105	0.966	202	1.000	539	0.992
264	0.991	322	0.999	29	0.967	203	1.000	746	0.992
307	0.991	391	0.999	72	0.967	208	1.000	777	0.992
353	0.991	413	0.999	129	0.967	209	1.000	75	0.993
454	0.991	414	0.999	123	0.968	228	1.000	100	0.993
538	0.991	475	0.999	349	0.968	229	1.000	113	0.993
560	0.991	500	0.999	177	0.969	235	1.000	153	0.993
602	0.991	513	0.999	356	0.969	252	1.000	160	0.993
626	0.991	545	0.999	283	0.970	253	1.000	191	0.993
25	0.992	567	0.999	254	0.971	257	1.000	199	0.993
80	0.992	580	0.999	319	0.971	283	1.000	273	0.993
86	0.992	599	0.999	228	0.972	288	1.000	361	0.993
91	0.992	611	0.999	311	0.972	300	1.000	378	0.993

Site	Prob[$\alpha > \beta$]	Site	Prob[$\alpha > \beta$]	Site	Prob[$\alpha > \beta$]	Site	Prob[$\alpha > \beta$]	Site	Prob[$\alpha > \beta$]
151	0.992	638	0.999	330	0.972	301	1.000	401	0.993
161	0.992	643	0.999	6	0.973	305	1.000	428	0.993
166	0.992	651	0.999	200	0.973	312	1.000	437	0.993
173	0.992	5	1.000	94	0.974	318	1.000	462	0.993
198	0.992	6	1.000	154	0.974	*VHL*		466	0.993
211	0.992	14	1.000	179	0.974	154	0.900	490	0.993
238	0.992	16	1.000	78	0.975	171	0.900	492	0.993
366	0.992	17	1.000	107	0.975	174	0.900	769	0.993
377	0.992	19	1.000	150	0.975	101	0.909	773	0.993
401	0.992	21	1.000	172	0.975	149	0.909	6	0.994
404	0.992	22	1.000	55	0.976	1	0.912	25	0.994
456	0.992	23	1.000	16	0.978	107	0.913	66	0.994
702	0.992	24	1.000	110	0.978	49	0.923	148	0.994
736	0.992	28	1.000	169	0.978	29	0.926	215	0.994
754	0.992	31	1.000	221	0.978	102	0.928	226	0.994
767	0.992	36	1.000	113	0.979	88	0.929	253	0.994
769	0.992	40	1.000	227	0.979	117	0.931	267	0.994
56	0.993	42	1.000	280	0.979	211	0.934	305	0.994
72	0.993	45	1.000	294	0.979	207	0.936	311	0.994
93	0.993	46	1.000	382	0.979	13	0.938	351	0.994
147	0.993	48	1.000	210	0.980	136	0.938	383	0.994
158	0.993	52	1.000	35	0.981	203	0.940	449	0.994
236	0.993	53	1.000	76	0.981	123	0.946	461	0.994
287	0.993	57	1.000	353	0.981	130	0.948	525	0.994
297	0.993	59	1.000	181	0.982	178	0.950	546	0.994
300	0.993	63	1.000	203	0.982	215	0.950	767	0.994
308	0.993	67	1.000	241	0.982	42	0.951	805	0.994
313	0.993	71	1.000	304	0.982	54	0.951	18	0.995
380	0.993	72	1.000	46	0.983	98	0.954	31	0.995
392	0.993	73	1.000	326	0.983	216	0.954	42	0.995
400	0.993	74	1.000	381	0.984	27	0.956	44	0.995

Site	Prob[$\alpha > \beta$]	Site	Prob[$\alpha > \beta$]	Site	Prob[$\alpha > \beta$]	Site	Prob[$\alpha > \beta$]	Site	Prob[$\alpha > \beta$]
545	0.993	75	1.000	24	0.985	85	0.956	92	0.995
671	0.993	76	1.000	132	0.985	151	0.956	94	0.995
760	0.993	80	1.000	341	0.985	116	0.959	107	0.995
764	0.993	84	1.000	41	0.986	201	0.959	127	0.995
62	0.994	87	1.000	231	0.987	14	0.965	158	0.995
65	0.994	90	1.000	262	0.987	20	0.965	170	0.995
140	0.994	92	1.000	274	0.987	106	0.965	192	0.995
169	0.994	94	1.000	285	0.987	193	0.966	209	0.995
337	0.994	95	1.000	337	0.987	190	0.967	212	0.995
363	0.994	98	1.000	369	0.987	64	0.968	233	0.995
509	0.994	100	1.000	401	0.987	186	0.969	249	0.995
674	0.994	103	1.000	20	0.988	90	0.970	251	0.995
682	0.994	106	1.000	146	0.988	38	0.972	262	0.995
762	0.994	109	1.000	161	0.988	119	0.972	271	0.995
779	0.994	110	1.000	286	0.988	162	0.975	335	0.995
785	0.994	111	1.000	317	0.988	56	0.977	382	0.995
103	0.995	117	1.000	388	0.988	63	0.979	442	0.995
120	0.995	119	1.000	12	0.989	110	0.982	477	0.995
142	0.995	120	1.000	18	0.989	140	0.982	519	0.995
381	0.995	122	1.000	282	0.989	165	0.982	535	0.995
430	0.995	123	1.000	125	0.990	80	0.983	578	0.995
623	0.995	124	1.000	36	0.991	170	0.984	584	0.995
133	0.996	125	1.000	47	0.991	39	0.985	702	0.995
195	0.996	126	1.000	112	0.991	83	0.985	718	0.995
277	0.996	127	1.000	124	0.991	26	0.986	37	0.996
278	0.996	130	1.000	153	0.991	150	0.988	93	0.996
292	0.996	131	1.000	199	0.991	15	0.989	97	0.996
306	0.996	133	1.000	229	0.991	93	0.989	106	0.996
309	0.996	134	1.000	325	0.991	45	0.990	229	0.996
320	0.996	135	1.000	327	0.991	46	0.991	256	0.996
327	0.996	137	1.000	331	0.991	164	0.991	280	0.996

Site	Prob[$\alpha > \beta$]	Site	Prob[$\alpha > \beta$]	Site	Prob[$\alpha > \beta$]	Site	Prob[$\alpha > \beta$]	Site	Prob[$\alpha > \beta$]
382	0.996	139	1.000	346	0.991	97	0.992	283	0.996
413	0.996	140	1.000	156	0.992	144	0.992	291	0.996
428	0.996	143	1.000	309	0.992	204	0.992	413	0.996
437	0.996	145	1.000	395	0.992	219	0.992	430	0.996
441	0.996	146	1.000	80	0.993	158	0.993	463	0.996
574	0.996	147	1.000	104	0.993	159	0.993	556	0.996
619	0.996	148	1.000	168	0.993	205	0.993	675	0.996
627	0.996	151	1.000	213	0.993	35	0.994	750	0.996
675	0.996	152	1.000	276	0.993	67	0.994	774	0.996
686	0.996	153	1.000	281	0.993	68	0.995	21	0.997
698	0.996	154	1.000	289	0.993	78	0.995	51	0.997
719	0.996	155	1.000	350	0.993	91	0.995	68	0.997
776	0.996	157	1.000	89	0.994	105	0.995	85	0.997
145	0.997	158	1.000	111	0.994	169	0.995	89	0.997
190	0.997	159	1.000	115	0.994	71	0.996	139	0.997
206	0.997	160	1.000	170	0.994	115	0.996	141	0.997
239	0.997	161	1.000	197	0.994	127	0.996	172	0.997
248	0.997	162	1.000	219	0.994	139	0.996	173	0.997
282	0.997	164	1.000	222	0.994	33	0.997	177	0.997
304	0.997	166	1.000	54	0.995	47	0.997	210	0.997
321	0.997	167	1.000	171	0.995	111	0.997	219	0.997
485	0.997	168	1.000	217	0.995	118	0.997	221	0.997
492	0.997	169	1.000	310	0.995	146	0.997	222	0.997
507	0.997	170	1.000	320	0.995	187	0.997	224	0.997
511	0.997	174	1.000	348	0.995	11	0.998	242	0.997
532	0.997	176	1.000	384	0.995	72	0.998	295	0.997
552	0.997	180	1.000	23	0.996	129	0.998	327	0.997
579	0.997	182	1.000	86	0.996	155	0.998	387	0.997
634	0.997	183	1.000	207	0.996	188	0.998	476	0.997
724	0.997	184	1.000	259	0.996	31	0.999	489	0.997
731	0.997	185	1.000	260	0.996	76	0.999	499	0.997

Site	Prob[$\alpha > \beta$]	Site	Prob[$\alpha > \beta$]	Site	Prob[$\alpha > \beta$]	Site	Prob[$\alpha > \beta$]	Site	Prob[$\alpha > \beta$]
734	0.997	186	1.000	321	0.996	77	0.999	515	0.997
8	0.998	189	1.000	338	0.996	95	0.999	562	0.997
55	0.998	190	1.000	392	0.996	124	0.999	583	0.997
61	0.998	191	1.000	205	0.997	126	0.999	697	0.997
69	0.998	196	1.000	252	0.997	128	0.999	810	0.997
70	0.998	197	1.000	290	0.997	133	0.999	33	0.998
88	0.998	201	1.000	303	0.997	143	0.999	87	0.998
92	0.998	202	1.000	335	0.997	152	0.999	122	0.998
168	0.998	203	1.000	42	0.998	177	0.999	181	0.998
182	0.998	204	1.000	50	0.998	189	0.999	196	0.998
193	0.998	205	1.000	100	0.998	5	1.000	243	0.998
210	0.998	208	1.000	114	0.998	43	1.000	341	0.998
223	0.998	209	1.000	162	0.998	61	1.000	581	0.998
245	0.998	212	1.000	188	0.998	65	1.000	656	0.998
260	0.998	215	1.000	214	0.998	86	1.000	802	0.998
261	0.998	217	1.000	244	0.998	99	1.000	17	0.999
288	0.998	218	1.000	287	0.998	103	1.000	23	0.999
326	0.998	220	1.000	297	0.998	120	1.000	26	0.999
343	0.998	221	1.000	299	0.998	121	1.000	27	0.999
356	0.998	222	1.000	323	0.998	122	1.000	29	0.999
362	0.998	225	1.000	375	0.998	125	1.000	30	0.999
399	0.998	228	1.000	393	0.998	132	1.000	38	0.999
458	0.998	232	1.000	400	0.998	134	1.000	57	0.999
466	0.998	233	1.000	10	0.999	135	1.000	70	0.999
475	0.998	234	1.000	33	0.999	138	1.000	73	0.999
497	0.998	235	1.000	43	0.999	153	1.000	76	0.999
502	0.998	236	1.000	49	0.999	156	1.000	78	0.999
562	0.998	251	1.000	68	0.999	157	1.000	90	0.999
648	0.998	253	1.000	83	0.999	160	1.000	111	0.999
723	0.998	256	1.000	85	0.999	161	1.000	136	0.999
744	0.998	260	1.000	92	0.999	163	1.000	162	0.999

Site	Prob[$\alpha > \beta$]	Site	Prob[$\alpha > \beta$]	Site	Prob[$\alpha > \beta$]	Site	Prob[$\alpha > \beta$]	Site	Prob[$\alpha > \beta$]
758	0.998	261	1.000	116	0.999	167	1.000	175	0.999
782	0.998	262	1.000	142	0.999	172	1.000	176	0.999
29	0.999	266	1.000	145	0.999	175	1.000	180	0.999
42	0.999	267	1.000	157	0.999	176	1.000	187	0.999
44	0.999	269	1.000	167	0.999	180	1.000	194	0.999
82	0.999	270	1.000	238	0.999	182	1.000	195	0.999
101	0.999	271	1.000	239	0.999	183	1.000	266	0.999
106	0.999	275	1.000	250	0.999	184	1.000	268	0.999
107	0.999	276	1.000	296	0.999	185	1.000	272	0.999
116	0.999	277	1.000	298	0.999	192	1.000	281	0.999
124	0.999	278	1.000	339	0.999	196	1.000	285	0.999
129	0.999	280	1.000	340	0.999	200	1.000	290	0.999
148	0.999	281	1.000	347	0.999	202	1.000	292	0.999
177	0.999	283	1.000	373	0.999	209	1.000	303	0.999
179	0.999	284	1.000	380	0.999	*VEGFA*		306	0.999
180	0.999	288	1.000	391	0.999	44	1.000	312	0.999
184	0.999	290	1.000	399	0.999	48	1.000	322	0.999
189	0.999	291	1.000	7	1.000	53	1.000	324	0.999
199	0.999	295	1.000	44	1.000	54	1.000	325	0.999
202	0.999	296	1.000	56	1.000	65	1.000	326	0.999
219	0.999	297	1.000	57	1.000	68	1.000	332	0.999
226	0.999	298	1.000	58	1.000	74	1.000	340	0.999
227	0.999	300	1.000	67	1.000	81	1.000	374	0.999
235	0.999	301	1.000	79	1.000	82	1.000	389	0.999
237	0.999	304	1.000	84	1.000	86	1.000	390	0.999
240	0.999	309	1.000	88	1.000	87	1.000	393	0.999
246	0.999	311	1.000	93	1.000	88	1.000	394	0.999
252	0.999	313	1.000	95	1.000	89	1.000	399	0.999
263	0.999	314	1.000	96	1.000	93	1.000	400	0.999
265	0.999	315	1.000	106	1.000	96	1.000	411	0.999
266	0.999	316	1.000	109	1.000	124	1.000	417	0.999

Site	Prob[$\alpha > \beta$]	Site	Prob[$\alpha > \beta$]	Site	Prob[$\alpha > \beta$]	Site	Prob[$\alpha > \beta$]	Site	Prob[$\alpha > \beta$]
269	0.999	323	1.000	118	1.000	212	1.000	423	0.999
270	0.999	324	1.000	120	1.000	5	0.999	434	0.999
310	0.999	329	1.000	127	1.000	9	0.999	452	0.999
322	0.999	332	1.000	131	1.000	12	0.999	467	0.999
328	0.999	335	1.000	175	1.000	27	0.965	468	0.999
335	0.999	338	1.000	176	1.000	85	0.999	470	0.999
346	0.999	339	1.000	183	1.000	105	0.999	479	0.999
351	0.999	340	1.000	186	1.000	120	0.999	485	0.999
375	0.999	341	1.000	191	1.000	147	0.999	540	0.999
386	0.999	343	1.000	192	1.000	78	0.998	543	0.999
390	0.999	344	1.000	194	1.000	208	0.998	545	0.999
397	0.999	345	1.000	195	1.000	16	0.997	548	0.999
405	0.999	348	1.000	196	1.000	181	0.997	549	0.999
412	0.999	349	1.000	202	1.000	91	0.996	570	0.999
445	0.999	351	1.000	204	1.000	152	0.995	631	0.999
449	0.999	352	1.000	209	1.000	193	0.995	639	0.999
469	0.999	353	1.000	215	1.000	117	0.994	662	0.999
472	0.999	356	1.000	218	1.000	158	0.993	666	0.999
486	0.999	357	1.000	223	1.000	28	0.992	683	0.999
489	0.999	358	1.000	225	1.000	24	0.991	703	0.999
504	0.999	361	1.000	226	1.000	71	0.990	716	0.999
539	0.999	362	1.000	230	1.000	108	0.990	717	0.999
596	0.999	364	1.000	234	1.000	13	0.985	722	0.999
606	0.999	366	1.000	237	1.000	72	0.982	740	0.999
612	0.999	367	1.000	240	1.000	111	0.981	748	0.999
621	0.999	372	1.000	242	1.000	42	0.979	752	0.999
662	0.999	378	1.000	247	1.000	66	0.976	779	0.999
676	0.999	381	1.000	248	1.000	114	0.976	807	0.999
677	0.999	382	1.000	253	1.000	64	0.974	812	0.999
685	0.999	383	1.000	255	1.000	100	0.970	20	1.000
696	0.999	385	1.000	257	1.000	213	0.970	32	1.000

Site	Prob[$\alpha > \beta$]	Site	Prob[$\alpha > \beta$]	Site	Prob[$\alpha > \beta$]	Site	Prob[$\alpha > \beta$]	Site	Prob[$\alpha > \beta$]
710	0.999	386	1.000	258	1.000	8	0.967	34	1.000
726	0.999	387	1.000	261	1.000	151	0.966	36	1.000
732	0.999	390	1.000	264	1.000	58	0.965	39	1.000
748	0.999	393	1.000	266	1.000	75	0.962	45	1.000
14	1.000	395	1.000	268	1.000	107	0.962	46	1.000
17	1.000	396	1.000	271	1.000	76	0.958	47	1.000
27	1.000	398	1.000	279	1.000	116	0.957	49	1.000
39	1.000	401	1.000	284	1.000	122	0.957	52	1.000
43	1.000	402	1.000	288	1.000	209	0.950	62	1.000
64	1.000	403	1.000	291	1.000	178	0.949	63	1.000
71	1.000	404	1.000	292	1.000	145	0.948	65	1.000
73	1.000	408	1.000	313	1.000	6	0.946	96	1.000
89	1.000	412	1.000	318	1.000	26	0.942	102	1.000
94	1.000	419	1.000	324	1.000	45	0.939	115	1.000
99	1.000	420	1.000	328	1.000	61	0.932	121	1.000
100	1.000	422	1.000	329	1.000	115	0.931	128	1.000
102	1.000	423	1.000	334	1.000	206	0.930	132	1.000
112	1.000	424	1.000	336	1.000	19	0.926	138	1.000
114	1.000	425	1.000	342	1.000	186	0.925	157	1.000
119	1.000	426	1.000	351	1.000	37	0.923	165	1.000
135	1.000	428	1.000	352	1.000	169	0.923	168	1.000
136	1.000	430	1.000	355	1.000	55	0.919	178	1.000
138	1.000	437	1.000	357	1.000	67	0.917	179	1.000
143	1.000	440	1.000	362	1.000	174	0.915	184	1.000
154	1.000	441	1.000	374	1.000	63	0.913	186	1.000
155	1.000	443	1.000	390	1.000	183	0.912	188	1.000
156	1.000	446	1.000	394	1.000	148	0.908	197	1.000
157	1.000	447	1.000	397	1.000	102	0.904	200	1.000
163	1.000	448	1.000	*EGLN3*		*HIF3A*		201	1.000
185	1.000	449	1.000	23	0.900	48	0.900	203	1.000
187	1.000	450	1.000	44	0.900	53	0.900	207	1.000

Site	Prob[$\alpha > \beta$]	Site	Prob[$\alpha > \beta$]	Site	Prob[$\alpha > \beta$]	Site	Prob[$\alpha > \beta$]	Site	Prob[$\alpha > \beta$]
191	1.000	451	1.000	80	0.903	218	0.900	211	1.000
192	1.000	453	1.000	156	0.904	287	0.901	216	1.000
200	1.000	454	1.000	189	0.904	577	0.901	218	1.000
207	1.000	455	1.000	211	0.904	106	0.903	230	1.000
208	1.000	456	1.000	239	0.905	141	0.906	238	1.000
209	1.000	457	1.000	83	0.907	217	0.907	241	1.000
214	1.000	458	1.000	27	0.908	614	0.907	245	1.000
217	1.000	459	1.000	131	0.908	81	0.908	247	1.000
222	1.000	462	1.000	110	0.909	269	0.908	254	1.000
228	1.000	464	1.000	59	0.912	485	0.908	260	1.000
230	1.000	465	1.000	236	0.921	604	0.908	261	1.000
232	1.000	469	1.000	26	0.923	26	0.914	264	1.000
234	1.000	470	1.000	102	0.924	17	0.915	265	1.000
249	1.000	471	1.000	20	0.931	20	0.915	279	1.000
254	1.000	473	1.000	221	0.931	22	0.915	284	1.000
256	1.000	474	1.000	1	0.932	24	0.915	293	1.000
258	1.000	476	1.000	7	0.932	40	0.915	299	1.000
262	1.000	478	1.000	128	0.932	43	0.915	313	1.000
281	1.000	479	1.000	144	0.932	54	0.915	314	1.000
286	1.000	480	1.000	90	0.934	205	0.915	329	1.000
299	1.000	483	1.000	121	0.934	225	0.915	331	1.000
302	1.000	484	1.000	208	0.934	239	0.915	333	1.000
303	1.000	487	1.000	82	0.935	334	0.915	336	1.000
305	1.000	489	1.000	222	0.939	596	0.915	338	1.000
311	1.000	490	1.000	160	0.942	602	0.916	342	1.000
316	1.000	491	1.000	69	0.943	33	0.917	363	1.000
332	1.000	492	1.000	207	0.943	77	0.917	384	1.000
349	1.000	493	1.000	91	0.946	101	0.917	385	1.000
358	1.000	494	1.000	180	0.946	589	0.917	386	1.000
359	1.000	496	1.000	30	0.949	94	0.918	388	1.000
365	1.000	497	1.000	229	0.949	482	0.919	403	1.000

Site	Prob[$\alpha > \beta$]	Site	Prob[$\alpha > \beta$]	Site	Prob[$\alpha > \beta$]	Site	Prob[$\alpha > \beta$]	Site	Prob[$\alpha > \beta$]
370	1.000	501	1.000	33	0.950	261	0.920	404	1.000
371	1.000	502	1.000	137	0.954	321	0.920	409	1.000
378	1.000	504	1.000	138	0.954	121	0.921	426	1.000
383	1.000	507	1.000	25	0.958	469	0.921	429	1.000
387	1.000	508	1.000	88	0.960	196	0.922	433	1.000
388	1.000	514	1.000	93	0.960	298	0.924	487	1.000
391	1.000	515	1.000	182	0.960	463	0.924	495	1.000
394	1.000	517	1.000	11	0.961	120	0.925	506	1.000
410	1.000	524	1.000	28	0.961	601	0.926	508	1.000
411	1.000	525	1.000	77	0.961	8	0.928	511	1.000
415	1.000	527	1.000	34	0.962	179	0.928	513	1.000
417	1.000	528	1.000	73	0.962	70	0.929	524	1.000
429	1.000	530	1.000	95	0.964	443	0.929	536	1.000
435	1.000	531	1.000	12	0.966	649	0.929	544	1.000
465	1.000	533	1.000	37	0.967	657	0.929	551	1.000
467	1.000	534	1.000	214	0.969	333	0.932	552	1.000
470	1.000	535	1.000	231	0.971	359	0.933	563	1.000
481	1.000	540	1.000	10	0.973	136	0.934	565	1.000
505	1.000	542	1.000	210	0.973	257	0.935	575	1.000
506	1.000	543	1.000	132	0.974	474	0.936	577	1.000
510	1.000	544	1.000	87	0.975	23	0.937	580	1.000
519	1.000	546	1.000	232	0.975	500	0.937	582	1.000
536	1.000	549	1.000	167	0.979	436	0.938	635	1.000
541	1.000	553	1.000	76	0.980	439	0.938	663	1.000
543	1.000	554	1.000	177	0.980	362	0.939	684	1.000
549	1.000	555	1.000	184	0.980	302	0.944	686	1.000
555	1.000	556	1.000	84	0.981	130	0.946	723	1.000
557	1.000	562	1.000	97	0.981	407	0.950	724	1.000
620	1.000	563	1.000	71	0.982	370	0.953	732	1.000
628	1.000	564	1.000	166	0.982	437	0.953	751	1.000
631	1.000	565	1.000	196	0.982	580	0.953	772	1.000

Site	Prob[$\alpha > \beta$]	Site	Prob[$\alpha > \beta$]	Site	Prob[$\alpha > \beta$]	Site	Prob[$\alpha > \beta$]	Site	Prob[$\alpha > \beta$]
643	1.000	566	1.000	197	0.982	477	0.954	776	1.000
645	1.000	570	1.000	63	0.984	537	0.954	795	1.000
665	1.000	571	1.000	205	0.984	140	0.955	800	1.000
681	1.000	573	1.000	230	0.984	112	0.956	814	1.000
688	1.000	575	1.000	66	0.985	461	0.956		
689	1.000	576	1.000	118	0.985	639	0.956		
695	1.000	577	1.000	133	0.986	295	0.959		
705	1.000	578	1.000	163	0.986	450	0.959		
715	1.000	579	1.000	147	0.987	444	0.961		
738	1.000	582	1.000	108	0.988	575	0.962		
739	1.000	588	1.000	183	0.988	50	0.963		
743	1.000	593	1.000	19	0.989	422	0.963		
745	1.000	595	1.000	99	0.989	533	0.963		
747	1.000	598	1.000	101	0.989	34	0.964		
757	1.000	600	1.000	127	0.989	358	0.964		
765	1.000	601	1.000	14	0.990	478	0.964		
781	1.000	602	1.000	60	0.990	659	0.964		
		604	1.000	94	0.990	109	0.965		
		606	1.000	174	0.991	396	0.965		
		607	1.000	49	0.992	591	0.965		
		608	1.000	105	0.992	606	0.965		
		610	1.000	107	0.992	184	0.966		
		614	1.000	117	0.992	307	0.966		
		615	1.000	124	0.993	353	0.966		
		616	1.000	148	0.993	29	0.967		
		619	1.000	29	0.994	67	0.967		
		620	1.000	36	0.994	110	0.967		
		623	1.000	85	0.994	178	0.967		
		624	1.000	89	0.994	255	0.967		
		625	1.000	143	0.994	535	0.967		
		626	1.000	153	0.994	565	0.967		

Site	Prob[$\alpha > \beta$]	Site	Prob[$\alpha > \beta$]	Site	Prob[$\alpha > \beta$]	Site	Prob[$\alpha > \beta$]	Site	Prob[$\alpha > \beta$]
		627	1.000	115	0.995	19	0.968		
		632	1.000	120	0.995	176	0.968		
		639	1.000	151	0.995	232	0.968		
		641	1.000	213	0.995	312	0.968		
		644	1.000	15	0.996	465	0.968		
		654	1.000	169	0.996	105	0.969		
		655	1.000	170	0.996	304	0.969		
		658	1.000	195	0.996	559	0.969		
		661	1.000	200	0.996	39	0.970		
		664	1.000	217	0.996	51	0.970		
		665	1.000	18	0.997	56	0.970		
		666	1.000	54	0.997	61	0.970		
		675	1.000	61	0.997	85	0.970		
		678	1.000	109	0.997	92	0.970		
		688	1.000	113	0.997	111	0.970		
		689	1.000	24	0.998	153	0.970		
		691	1.000	32	0.998	168	0.970		
		692	1.000	57	0.998	447	0.970		
		693	1.000	86	0.998	494	0.970		
		694	1.000	129	0.998	306	0.971		
		697	1.000	139	0.998	96	0.972		
		698	1.000	150	0.998	118	0.972		
		702	1.000	194	0.998	223	0.972		
		703	1.000	224	0.998	475	0.972		
		704	1.000	3	0.999	235	0.973		
		707	1.000	22	0.999	529	0.973		
		709	1.000	38	0.999	654	0.973		
		713	1.000	43	0.999	25	0.974		
		714	1.000	75	0.999	342	0.974		
		715	1.000	96	0.999	603	0.974		
		716	1.000	103	0.999	212	0.975		

Site	Prob[$\alpha > \beta$]	Site	Prob[$\alpha > \beta$]	Site	Prob[$\alpha > \beta$]	Site	Prob[$\alpha > \beta$]	Site	Prob[$\alpha > \beta$]
				125	0.999	456	0.975		
				126	0.999	531	0.975		
				171	0.999	157	0.977		
				190	0.999	335	0.977		
				233	0.999	457	0.978		
				238	0.999	543	0.978		
				2	1.000	49	0.979		
				13	1.000	113	0.979		
				21	1.000	160	0.979		
				35	1.000	192	0.979		
				40	1.000	241	0.979		
				42	1.000	481	0.979		
				46	1.000	487	0.979		
				47	1.000	488	0.979		
				62	1.000	90	0.980		
				64	1.000	98	0.980		
				65	1.000	291	0.980		
				67	1.000	428	0.980		
				74	1.000	493	0.981		
				100	1.000	28	0.982		
				106	1.000	290	0.982		
				116	1.000	377	0.982		
				130	1.000	479	0.983		
				134	1.000	12	0.984		
				135	1.000	59	0.984		
				136	1.000	270	0.984		
				140	1.000	411	0.984		
				141	1.000	100	0.985		
				142	1.000	211	0.985		
				145	1.000	240	0.985		
				155	1.000	468	0.985		

Site	Prob[$\alpha > \beta$]	Site	Prob[$\alpha > \beta$]	Site	Prob[$\alpha > \beta$]	Site	Prob[$\alpha > \beta$]	Site	Prob[$\alpha > \beta$]
				157	1.000	491	0.985		
				161	1.000	545	0.985		
				162	1.000	648	0.985		
				165	1.000	5	0.986		
				172	1.000	18	0.986		
				173	1.000	76	0.986		
				176	1.000	95	0.986		
				179	1.000	108	0.986		
				185	1.000	224	0.986		
				186	1.000	343	0.986		
				191	1.000	345	0.986		
				192	1.000	384	0.986		
				193	1.000	509	0.986		
				198	1.000	552	0.986		
				201	1.000	579	0.986		
				202	1.000	214	0.987		
				203	1.000	273	0.987		
				206	1.000	301	0.987		
				215	1.000	303	0.987		
				218	1.000	480	0.987		
				219	1.000	502	0.987		
						627	0.987		
						635	0.987		
						27	0.988		
						55	0.988		
						199	0.988		
						215	0.988		
						245	0.988		
						286	0.988		
						324	0.988		

Site	Prob[$\alpha > \beta$]	Site	Prob[$\alpha > \beta$]	Site	Prob[$\alpha > \beta$]	Site	Prob[$\alpha > \beta$]	Site	Prob[$\alpha > \beta$]
						414	0.988		
						435	0.988		
						131	0.989		
						169	0.989		
						337	0.989		
						418	0.989		
						578	0.989		
						89	0.990		
						116	0.990		
						127	0.990		
						151	0.990		
						238	0.990		
						263	0.990		
						266	0.990		
						281	0.990		
						289	0.990		
						297	0.990		
						315	0.990		
						403	0.990		
						80	0.991		
						147	0.991		
						161	0.991		
						226	0.991		
						229	0.991		
						233	0.991		
						294	0.991		
						492	0.991		
						534	0.991		
						562	0.991		
						588	0.991		
						10	0.992		

Site	Prob[$\alpha > \beta$]	Site	Prob[$\alpha > \beta$]	Site	Prob[$\alpha > \beta$]	Site	Prob[$\alpha > \beta$]	Site	Prob[$\alpha > \beta$]
						83	0.992		
						158	0.992		
						180	0.992		
						198	0.992		
						204	0.992		
						236	0.992		
						336	0.992		
						383	0.992		
						416	0.992		
						449	0.992		
						519	0.992		
						582	0.992		
						13	0.993		
						91	0.993		
						220	0.993		
						230	0.993		
						405	0.993		
						459	0.993		
						466	0.993		
						570	0.993		
						573	0.993		
						47	0.994		
						78	0.994		
						99	0.994		
						191	0.994		
						308	0.994		
						319	0.994		
						354	0.994		
						511	0.994		
						568	0.994		
						104	0.995		

Site	Prob[$\alpha > \beta$]	Site	Prob[$\alpha > \beta$]	Site	Prob[$\alpha > \beta$]	Site	Prob[$\alpha > \beta$]	Site	Prob[$\alpha > \beta$]
						144	0.995		
						149	0.995		
						506	0.995		
						532	0.995		
						563	0.995		
						574	0.995		
						6	0.996		
						42	0.996		
						185	0.996		
						209	0.996		
						311	0.996		
						322	0.996		
						323	0.996		
						355	0.996		
						438	0.996		
						572	0.996		
						7	0.997		
						74	0.997		
						119	0.997		
						123	0.997		
						129	0.997		
						202	0.997		
						213	0.997		
						274	0.997		
						305	0.997		
						326	0.997		
						346	0.997		
						423	0.997		
						483	0.997		
						486	0.997		
						538	0.997		

Site	Prob[$\alpha > \beta$]	Site	Prob[$\alpha > \beta$]	Site	Prob[$\alpha > \beta$]	Site	Prob[$\alpha > \beta$]	Site	Prob[$\alpha > \beta$]
						539	0.997		
						553	0.997		
						571	0.997		
						16	0.998		
						45	0.998		
						52	0.998		
						63	0.998		
						115	0.998		
						132	0.998		
						143	0.998		
						150	0.998		
						162	0.998		
						170	0.998		
						172	0.998		
						177	0.998		
						181	0.998		
						208	0.998		
						228	0.998		
						244	0.998		
						283	0.998		
						284	0.998		
						285	0.998		
						288	0.998		
						317	0.998		
						318	0.998		
						331	0.998		
						332	0.998		
						351	0.998		
						404	0.998		
						424	0.998		
						473	0.998		

Site	Prob[$\alpha > \beta$]	Site	Prob[$\alpha > \beta$]	Site	Prob[$\alpha > \beta$]	Site	Prob[$\alpha > \beta$]	Site	Prob[$\alpha > \beta$]
						484	0.998		
						489	0.998		
						516	0.998		
						518	0.998		
						528	0.998		
						540	0.998		
						546	0.998		
						592	0.998		
						597	0.998		
						605	0.998		
						9	0.999		
						11	0.999		
						14	0.999		
						37	0.999		
						75	0.999		
						87	0.999		
						126	0.999		
						134	0.999		
						154	0.999		
						159	0.999		
						183	0.999		
						186	0.999		
						190	0.999		
						197	0.999		
						216	0.999		
						219	0.999		
						243	0.999		
						251	0.999		
						264	0.999		
						292	0.999		
						300	0.999		

Site	Prob[$\alpha > \beta$]	Site	Prob[$\alpha > \beta$]	Site	Prob[$\alpha > \beta$]	Site	Prob[$\alpha > \beta$]	Site	Prob[$\alpha > \beta$]
						313	0.999		
						330	0.999		
						348	0.999		
						381	0.999		
						382	0.999		
						392	0.999		
						409	0.999		
						415	0.999		
						514	0.999		
						524	0.999		
						536	0.999		
						583	0.999		
						613	0.999		
						617	0.999		
						643	0.999		
						2	1.000		
						4	1.000		
						15	1.000		
						30	1.000		
						32	1.000		
						35	1.000		
						36	1.000		
						38	1.000		
						41	1.000		
						46	1.000		
						57	1.000		
						62	1.000		
						65	1.000		
						88	1.000		
						93	1.000		
						102	1.000		

Site	Prob[$\alpha > \beta$]	Site	Prob[$\alpha > \beta$]	Site	Prob[$\alpha > \beta$]	Site	Prob[$\alpha > \beta$]	Site	Prob[$\alpha > \beta$]
						103	1.000		
						107	1.000		
						114	1.000		
						117	1.000		
						122	1.000		
						124	1.000		
						128	1.000		
						133	1.000		
						135	1.000		
						138	1.000		
						139	1.000		
						152	1.000		
						156	1.000		
						163	1.000		
						167	1.000		
						174	1.000		
						175	1.000		
						188	1.000		
						193	1.000		
						200	1.000		
						206	1.000		
						207	1.000		
						210	1.000		
						221	1.000		
						227	1.000		
						231	1.000		
						242	1.000		
						246	1.000		
						247	1.000		
						250	1.000		
						252	1.000		

Site	Prob[$\alpha > \beta$]	Site	Prob[$\alpha > \beta$]	Site	Prob[$\alpha > \beta$]	Site	Prob[$\alpha > \beta$]	Site	Prob[$\alpha > \beta$]
						253	1.000		
						254	1.000		
						256	1.000		
						258	1.000		
						260	1.000		
						262	1.000		
						265	1.000		
						267	1.000		
						271	1.000		
						272	1.000		
						275	1.000		
						276	1.000		
						277	1.000		
						278	1.000		
						293	1.000		
						296	1.000		
						310	1.000		
						316	1.000		
						320	1.000		
						325	1.000		
						328	1.000		
						339	1.000		
						344	1.000		
						347	1.000		
						365	1.000		
						374	1.000		
						386	1.000		
						387	1.000		
						390	1.000		
						391	1.000		
						395	1.000		

Site	Prob[$\alpha > \beta$]	Site	Prob[$\alpha > \beta$]	Site	Prob[$\alpha > \beta$]	Site	Prob[$\alpha > \beta$]	Site	Prob[$\alpha > \beta$]
						398	1.000		
						399	1.000		
						400	1.000		
						402	1.000		
						406	1.000		
						412	1.000		
						420	1.000		
						426	1.000		
						434	1.000		
						441	1.000		
						490	1.000		
						497	1.000		
						513	1.000		
						515	1.000		
						520	1.000		
						523	1.000		
						584	1.000		
						585	1.000		
						586	1.000		
						595	1.000		
						599	1.000		
						634	1.000		

Prob [$\alpha>\beta$]: posterior probability of negative selection at a site.
