# Supplementary material for: Adaptive strategies of aquatic mammals: Exploring the role of the HIF pathway and hypoxia tolerance
Source: Genet Mol Biol. 2024 Jan 19;46(3 Suppl 1):e20230140. doi: 10.1590/1678-4685-GMB-2023-0140 (PMC10802827; doi:10.1590/1678-4685-GMB-2023-0140)
Supplement: Table S10 - [file 1415-4757-GMB-46-03-s1-e20230140-s10.pdf]

Genetics and Molecular Biology
https://doi.org/10.1590/1678-4685-GMB-2023-0140

Supplementary Material to "Adaptive strategies of aquatic mammals: Exploring the role of the HIF pathway and hypoxia tolerance"

Table S10 - Codeml nested branch-level model.

Model	lnL	gl	LRT		p-value	Terrestrial mammals ω	ω			
							Shallow-divers		Deep-divers	
							Ancestral	Descendant	Ancestral	Descendant
				ARNT						
				Cetacea						
1ω	-53375.76281	116				0.00017	0.00017			
2ω	-51964.26991	117	2ω vs. 1ω	2822.98581	0	0.00012	0.0001			
3ω	-46102.18521	118	3ω vs. 2ω	11724.16939	0	0.00015	0.0001		0.0001	
5ω	-45327.32658	120	5ω vs. 2ω	1549.71726	0	0.00014	0.0001	0.0001	0.0001	0.0001
				Pinnipeda						
1ω	-36919.66885	84				0.00026				
2ω	-34938.06976	85	2ω vs. 1ω	3963.19818	0	0.00018	0.0001			
3ω	-41170.68038	86	3ω vs. 2ω	-12465.22123	1	0.0001	0.04824		0.0001	
5ω	-61039.46154	88	5ω vs. 2ω	-39737.56232	1	0.0001	0.32911	0.22149	0.40497	0.18671

Model	lnL	gl	LRT		p-value	Terrestrial mammals ω	ω			
							Shallow-divers		Deep-divers	
							Ancestral	Descendant	Ancestral	Descendant
ARNT2										
Cetacea										
1ω	-37502.44941	114				0.0001	0.0001			
2ω	-28452.78358	115	2ω vs. 1ω	18099.33166	0	0.0001	0.0001			
3ω	-29280.19136	116	3ω vs. 2ω	-1654.81556	1	0.0001	0.0001		0.0001	
5ω	-33173.0808	118	5ω vs. 2ω	-7785.77887	1	0.0001	0.0001	0.0001	0.0001	0.0001
Pinnipeda										
1ω	-30420.04097	84				0.0001	0.0001			
2ω	-25369.12442	85	2ω vs. 1ω	10101.83310	0	0.0001	0.00011			
3ω	-23456.25239	86	3ω vs. 2ω	3825.74405	0	0.0001	0.0001		0.0188	
5ω	-25952.19338	88	5ω vs. 2ω	-4991.88198	1	0.0001	0.0001	0.0001	0.00016	0.00014
EGLN1										
Cetacea										
1ω	-21183.80407	112				0.00016	0.00016			
2ω	-18274.37601	113	2ω vs. 1ω	5818.85612	0	0.00014	0.0001			
3ω	-19018.28267	114	3ω vs. 2ω	-1487.81332	1	0.00014	0.0001		0.0001	
5ω	-34730.05067	116	5ω vs. 2ω	-31423.53600	1	0.0001	0.377	0.0001	0.25238	0.03406
Pinnipeda										
1ω	-17490.71486	80				0.00017	0.00017			
2ω	-15809.29783	81	2ω vs. 1ω	3362.83408	0	0.00021	0.0001			
3ω	-15806.76303	82	3ω vs. 2ω	5.06959	0.024	0.00021	0.0001		0.0001	
5ω	-15441.92043	84	5ω vs. 2ω	729.68521	0	0.00021	0.0001	0.0001	0.0001	0.0001
EGLN2										

Model	lnL	gl	LRT		p-value	Terrestrial mammals ω	ω			
							Shallow-divers		Deep-divers	
							Ancestral	Descendant	Ancestral	Descendant
Cetacea										
1ω	-27949.12198	118				0.00026	0.00026			
2ω	-27264.97104	119	2ω vs. 1ω	1368.30188	0	0.0001	0.0001			
3ω	-37889.30512	120	3ω vs. 2ω	-21248.66816	1	0.00023	0.0001		0.13261	
5ω	-26408.53222	122	5ω vs. 2ω	22961.54581	0	0.00029	0.0001	0.00025	0.0001	0.0001
Pinnipeda										
1ω	-21016.99147	88				0.00027	0.00027			
2ω	-20023.85858	89	2ω vs. 1ω	1986.26577	0	0.0003	0.0001			
3ω	-33060.50234	90	3ω vs. 2ω	-26073.28752	0	0.0001	0.21953		0.18494	
5ω	-27567.02917	93	5ω vs. 2ω	10986.94634	1	0.0001	0.47962	0.12338	0.36743	0.0001
EGLN3										
Cetacea										
1ω	-11823.20249	116				0.0001	0.0001			
2ω	-10957.58829	117	2ω vs. 1ω	1731.22840	0	0.0001	0.0001			
3ω	-8380.729419	118	3ω vs. 2ω	5153.71774	0	0.0001	0.03248		0.000102545	
5ω	-7809.163664	120	5ω vs. 2ω	1143.13151	0	0.0001	0.0001	0.06984	0.0001	0.05301
Pinnipeda										
1ω	-10931.96992	90				0.0001	0.0001			
2ω	-7395.629567	91	2ω vs. 1ω	7072.68071	0	0.00021	0.01691			
3ω	-7890.74967	92	3ω vs. 2ω	-990.24021	1	0.0001	0.0001		0.0548	
5ω	-9569.675787	94	5ω vs. 2ω	-3357.85223	1	0.0001	0.0001	0.0001	0.00011	0.0001
EPAS1										
Cetacea										

							ω			
**Model**	**lnL**	**gl**	**LRT**		**p-value**	**Terrestrial mammals ω**	**Shallow-divers**		**Deep-divers**	
							**Ancestral**	**Descendant**	**Ancestral**	**Descendant**
1ω	-55796.58414	118				0.0003	0.0003			
2ω	-104237.1577	119	2ω vs. 1ω	-96881.14720	1	0.0001	0.06347			
3ω	-52824.25669	120	3ω vs. 2ω	102825.80208	0	0.00016	0.0001		0.0001	
5ω	-98020.41548		5ω vs. 2ω	-90392.31757	1	0.0001	0.57554	0.72097	0.55573	0.0001
Pinnipeda										
1ω	-45272.11057	88				0.00029	0.00029			
2ω	-71636.18933	89	2ω vs. 1ω	-52728.15753	1	0.0001	0.19126			
3ω	-70614.51066	90	3ω vs. 2ω	2043.35735	0	0.0001	0.13071		0.37657	
5ω	-42860.12371	92	5ω vs. 2ω	55508.77390	0	0.00025	0.00029	0.0001	0.0054	0.0001
HIF1A										
Cetacea										
1ω	-51849.72	116				0.00015	0.00015			
2ω	-53784.42656	117	2ω vs. 1ω	-3869.41312	1	0.0001	0.0001			
3ω	-100151.3228	118	3ω vs. 2ω	-92733.79252	1	0.00023	0.37966		0.25663	
5ω	-97758.39552	120	5ω vs. 2ω	4785.85460	0	0.00023	0.26884	0.5028	0.27794	0.09102
Pinnipeda										
1ω	-40968.65063	88				0.00018	0.00018			
2ω	-37493.55981	89	2ω vs. 1ω	6950.18165	0	0.00022	0.00546			
3ω	-34430.87932	90	3ω vs. 2ω	6125.36097	0	0.00022	0.0001		0.0001	
5ω	-32647.7158	92	5ω vs. 2ω	3566.32705	0	0.00012	0.00072	0.0001	0.00135	0.0001
HIF1AN										
Cetacea										
1ω	-21786.36857	116				0.0001	0.0001			

Model	lnL	gl	LRT		p-value	Terrestrial mammals ω	ω			
							Shallow-divers		Deep-divers	
							Ancestral	Descendant	Ancestral	Descendant
2ω	-17179.35771	117	2ω vs. 1ω	9214.02174	0	0.0001	0.0001			
3ω	-13815.03912	118	3ω vs. 2ω	6728.63717	0	0.0001	0.0001		0.00011	
5ω	-15739.43541	120	5ω vs. 2ω	-3848.79258	1	0.0001	0.0001	0.0001	0.00012	0.0001
Pinnipeda										
1ω	-17291.02334	88				0.0001	0.0001			
2ω	-10356.93988	89	2ω vs. 1ω	13868.16693	0	0.00015	0.00615			
3ω	-10752.8836	90	3ω vs. 2ω	-791.88744	1	0.00011	0.08106		0.22489	
5ω	-10760.16125	92	5ω vs. 2ω	-14.55532	1	0.0001	1.91572	0.05237	0.0001	4.34542
HIF3A										
Cetacea										
1ω	-39452.27681	108				0.00026	0.00026			
2ω	-35515.01434	109	2ω vs. 1ω	7874.52494	0	0.00035	0.0001			
3ω	-62770.13321	110	3ω vs. 2ω	-54510.23775	1	0.0001	0.39291		0.0001	
5ω	-36004.18624	112	5ω vs. 2ω	53531.89394	0	0.00033	0.0001	0.0001	0.0001	0.0001
Pinnipeda										
1ω	-34574.89803	82				0.00034	0.00034			
2ω	-46056.29297	83	2ω vs. 1ω	-22962.78987	1	0.0001	0.04553			
3ω	-31973.45322	84	3ω vs. 2ω	28165.67949	0	0.00044	0.0001		0.00011	
5ω	-43265.24294	86	5ω vs. 2ω	-22583.57943	0	0.0001	0.46016	0.10449	0.37359	0.0001
VEGFA										
Cetacea										
1ω	-12389.23226	108				0.00016	0.00016			
2ω	-9354.548853	109	2ω vs. 1ω	6069.36681	0	0.0001	0.0001			

Model	lnL	gl	LRT		p-value	Terrestrial mammals ω	ω			
							**Shallow-divers**		**Deep-divers**	
							**Ancestral**	**Descendant**	**Ancestral**	**Descendant**
3ω	-9346.325678	110	3ω vs. 2ω	16.44635	0	0.0001	0.0001		0.0001	
5ω	-9514.620812	112	5ω vs. 2ω	-336.59027	1	0.00011	0.0001	0.0001	0.0001	0.0001
Pinnipeda										
1ω	-7037.60925	76				0.00011	0.00011			
2ω	-7172.312058	77	2ω vs. 1ω	-269.40562	1	0.00011	0.0001			
3ω	-7068.219904	78	3ω vs. 2ω	208.18431	0	0.00012	0.0001		0.0001	
5ω	-7327.332158	80	5ω vs. 2ω	-518.22451	1	0.0001	0.0001	0.0001	0.0001	0.0001
VHL										
Cetacea										
1ω	-11082.53938	110				0.00028	0.00028			
2ω	-9135.507165	111	2ω vs. 1ω	3894.06442	1	0.00025	0.0001			
3ω	-9764.501525	112	3ω vs. 2ω	-1257.98872	0	0.00025	0.0001		0.0001	
5ω	-9416.759381	114	5ω vs. 2ω	695.48429	1	0.00025	0.00015	0.0001	0.0001	0.0001
Pinnipeda										
1ω	-7733.041293	82				0.00025	0.00025			
2ω	-7618.522656	83	2ω vs. 1ω	229.03727	0	0.00027	0.0001			
3ω	-7623.682993	84	3ω vs. 2ω	-10.32067	1	0.00026	0.0001		0.0001	
5ω	-7878.81416	86	5ω vs. 2ω	-510.26233	1	0.00026	0.0001	0.0001	0.0001	0.0001
