# Supplementary material for: Adaptive strategies of aquatic mammals: Exploring the role of the HIF pathway and hypoxia tolerance
Source: Genet Mol Biol. 2024 Jan 19;46(3 Suppl 1):e20230140. doi: 10.1590/1678-4685-GMB-2023-0140 (PMC10802827; doi:10.1590/1678-4685-GMB-2023-0140)
Supplement: Figure S3 - [file 1415-4757-GMB-46-03-s1-e20230140-s13.pdf]

*Genetics and Molecular Biology*
https://doi.org/10.1590/1678-4685-GMB-2023-0140

# Supplementary Material to "Adaptive strategies of aquatic mammals: Exploring the role of the HIF pathway and hypoxia tolerance"

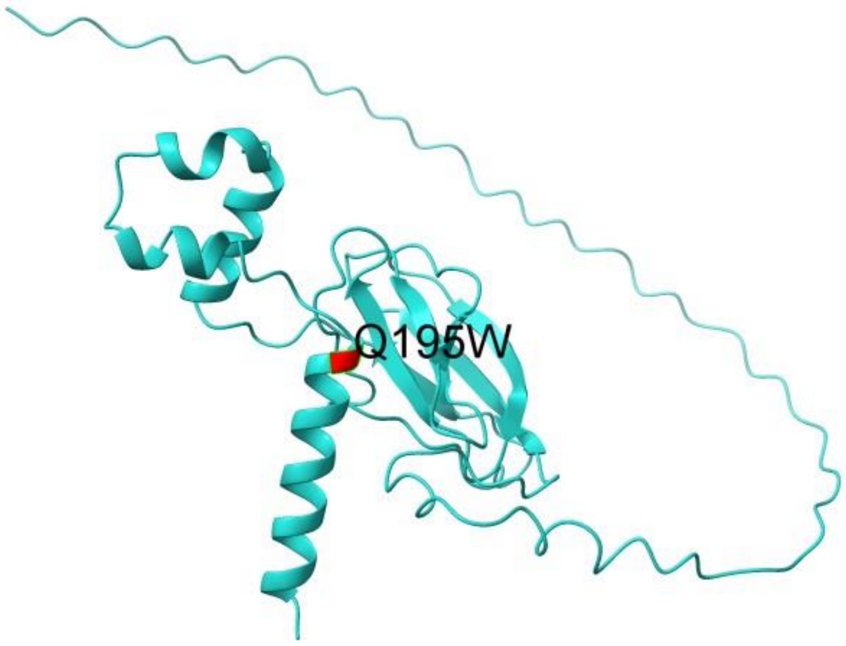


**Figure S3 –** Molecular visualization of mutation (site 195) in the AlphaFold three-dimensional model of VHL.
